# Complete Genome Sequence of *Thermus aquaticus* Y51MC23

**DOI:** 10.1371/journal.pone.0138674

**Published:** 2015-10-14

**Authors:** Phillip J. Brumm, Scott Monsma, Brendan Keough, Svetlana Jasinovica, Erin Ferguson, Thomas Schoenfeld, Michael Lodes, David A. Mead

**Affiliations:** 1 C5-6 Technologies LLC, Fitchburg, Wisconsin, United States of America; 2 Lucigen Corporation, Middleton, Wisconsin, United States of America; Osaka University, JAPAN

## Abstract

*Thermus aquaticus* Y51MC23 was isolated from a boiling spring in the Lower Geyser Basin of Yellowstone National Park. Remarkably, this *T*. *aquaticus* strain is able to grow anaerobically and produces multiple morphological forms. Y51MC23 is a Gram-negative, rod-shaped organism that grows well between 50°C and 80°C with maximum growth rate at 65°C to 70°C. Growth studies suggest that Y51MC23 primarily scavenges protein from the environment, supported by the high number of secreted and intracellular proteases and peptidases as well as transporter systems for amino acids and peptides. The genome was assembled *de novo* using a 350 bp fragment library (paired end sequencing) and an 8 kb long span mate pair library. A closed and finished genome was obtained consisting of a single chromosome of 2.15 Mb and four plasmids of 11, 14, 70, and 79 kb. Unlike other *Thermus* species, functions usually found on megaplasmids were identified on the chromosome. The Y51MC23 genome contains two full and two partial prophage as well as numerous CRISPR loci. The high identity and synteny between Y51MC23 prophage 2 and that of *Thermus* sp. 2.9 is interesting, given the 8,800 km separation of the two hot springs from which they were isolated. The anaerobic lifestyle of Y51MC23 is complex, with multiple morphologies present in cultures. The use of fluorescence microscopy reveals new details about these unusual morphological features, including the presence of multiple types of large and small spheres, often forming a confluent layer of spheres. Many of the spheres appear to be formed not from cell envelope or outer membrane components as previously believed, but from a remodeled peptidoglycan cell wall. These complex morphological forms may serve multiple functions in the survival of the organism, including food and nucleic acid storage as well as colony attachment and organization.

## Introduction

*Thermus aquaticus* YT-1 holds a special place in the history of microbiology. The thermophile was first isolated and cultured from a hot spring in Yellowstone National Park in 1969 [1]. The discovery of life at high temperatures was controversial at that time, but later shown to be quite prevalent as demonstrated by the isolation of *Thermus* strains from hot water heaters and other sources [2]. The subsequent discovery and characterization of *Thermus aquaticus* DNA polymerase resulted in the development of amplification and sequencing tools that have revolutionized nearly every field of biology and medicine [3].

*Thermus* species that have been isolated from hot springs around the globe include *T*. *brockianus*, *T*. *thermophilus* [3], *T*. *oshimai* [4], *T*. *caliditerrae* [5], *T*. *arciformis* [6], *T*. *islandicus* [7] *T*. *igniterrae* [8] and *T*. *antranikianii* [8]. Not all *Thermus* species have been found in hot springs. *T*. *composti* was isolated from an oyster mushroom compost [9] and *T*. *scotoductus* from a South African gold mine [10]. A number of isolates initially classified as *Thermus* species have been reclassified as *Meiothermus* species [11], based on phylogenetic and physiological differences such as lower optimum growth temperatures.

A unique feature of *Thermus aquaticus* YT-1 is its unusual cellular morphology. Shortly after the initial *T*. *aquaticus* report, Brock described the presence of “rotund bodies”, which appeared to be an association of multiple cells connected by a combined outer envelope as visualized by electron microscopy [12]. Little is known about the prevalence or function of these rotund bodies; however, the only other organism reported to form them is *Meiothermus ruber* (formerly *Thermus ruber*) [13–15]. Because of the low levels of rotund bodies observed in cultures, it is unclear if their production is limited to *T*. *aquaticus* and a few other related species, or if it is a trait shared by all *Thermus* species. Micrographs demonstrating the remarkable morphological diversity of *Thermus aquaticus* Y51MC23 are presented here.

There were 28 *Thermus* genome projects as of April 2015 (Genomes online database, https://gold.jgi-psf.org/index), 23 of which have retrievable sequence data. Complete genome sequences have been reported for *T*. *thermophilus* HB8 [16], *T*. *thermophilus* HB27[17], *T*. *scotoductus* SA-01 [10], *T*. *oshimai* JL-2 and *T*. *thermophilus* JL-18 [18, 19]. Most *Thermus* genomes have been left unfinished in permanent draft status (17/23), including *Thermus* sp. strain RL [20], *Thermus* sp. strain CCB_US3_UF1 [21], *Thermus* sp. 2.9 [22], *T*. *thermophilus* ATCC 33923 [23], and *T*. *aquaticus* Y51MC23 (http://www.ncbi.nlm.nih.gov/nuccore/218297404). The ability to close and finish microbial genomes is constrained by the lack of tools for connecting short read sequence data across long repeats. This paper describes the complete genome sequence for *Thermus aquaticus* Y51MC23 and its four plasmids, the first for this species, which was closed and finished using a new mate pair library construction tool. This complete genome is a valuable reference for the comparative analysis of the organization and structure of *Thermus* genomes.

## Methods

### Isolation, Growth Conditions and DNA Isolation

*Thermus aquaticus* Y51MC23 (Y51MC23) was isolated from a sample of hot spring water by enrichment and plating on YTP-2 agar at 70°C [24] and subsequently maintained on YTP-2 agar plates. The sample was collected under Scientific Research and Collecting Permit YELL-2001-SCI-0221 issued by the US Department of the Interior National Park Service, Yellowstone National Park. The culture is available from the ATCC™. For preparation of genomic DNA, cultures of Y51MC23 were grown from a single colony in 1000 ml YTP-2 medium in a 2000 ml Erlenmeyer flask at 70°C, 200 rpm for 18 hours. Cells were collected by centrifugation at 4°C and stored frozen until used for DNA preparation. The cell concentrate was lysed using a combination of SDS and proteinase K, and genomic DNA was isolated using a phenol/chloroform extraction method [25].

Aerobic growth was performed using either 50 ml YTP-2 media in a 250 ml flask, or 1000 ml YTP-2 media in a 2000 ml flask, at 70°C, 200 rpm, with silicone foam closure. Anaerobic growth was performed using YTP-2 medium with nitrate omitted. Cultures were grown in either 50 ml of media in 50 ml conical, screw cap tube or 1000 ml media in 1000 ml screw cap bottle, at 70°C with no agitation.

### Microscopy

Culture samples were treated using either a 5 μM solution of SYTO® 9 fluorescent stain, Live-Dead® Stain (SYTO® 9 and propridium bromide), or ViaGram™ Red^+^ Bacterial Gram Stain and Viability Kit (SYTOX® Green nucleic acid stain, 4,6-diamidino-2-phenylindole and Texas Red®-X dye–labeled wheat germ agglutinin) in sterile water (Life Technologies). Dark field fluorescence microscopy was performed using a Nikon Eclipse TE2000-S epifluorescence microscope at 20× or 2000× magnification using a high-pressure Hg light source.

### Genome Sequencing and Assembly

A permanent draft genome of *Thermus aquaticus* Y51MC23 containing 22 contigs was deposited at NCBI in 2008 (http://www.ncbi.nlm.nih.gov/nuccore/218297404) by the Joint Genome Institute (JGI) (Walnut Creek, CA). The draft genome was sequenced using a combination of Illumina fragment libraries and Sanger chemistry. The same culture isolate used for that effort was re-sequenced using the methods described below. Both fragment and 8 kb mate pair libraries were constructed for sequencing using NxSeq® DNA Sample Prep and Long Mate Pair Library Kits (Lucigen, Middleton, WI), respectively. Fragment libraries were constructed by shearing genomic DNA to approximately 300–400 with a Covaris LE220 Focused-Ultrasonicator (Covaris, Woburn, MA), end repairing, A-tailing and ligating Illumina compatible adaptors. The adapted DNA was then size selected with AMPure XP beads (Beckman Coulter, Brea CA). An eight kb mate pair library was constructed by shearing genomic DNA with a g-TUBE (Covaris), followed by end repair, A-tailing and ligation of adaptors. Adapted DNA was then size-selected with AMPure XP beads, ligated to a coupler, exonuclease treated, digested with restriction endonucleases and purified prior to circularization with a junction adaptor and PCR amplification. Libraries were sequenced on a MiSeq using Reagent Kit v2 (Illumina, San Diego, CA). Genomic sequence assembly and analysis were carried out using CLC Genomics Workbench v7 (Qiagen, Boston, MA).

DNA extracts were loaded onto a 1% Pulsed Field Certified (BioRad) agarose gel along with LR PFG markers (NEB) and run on a CHEF MAPPER XA system (BioRad) under the following conditions calculated by the MAPPER AutoAlgorithm function for 1–200 kb separation: 0.06–17.33 second linear ramping over 7.53 h run time at 6 V/cm in 1X TAE at 12°C with a 120° reorientation angle. The gel was post-stained with EtBr, destained, and visualized on a GelDoc XR+ system (BioRad).

### PCR Validation of JGI Draft and Lucigen Finished Assemblies of Y51MC23

Both the JGI draft and the Lucigen finished genome assemblies were verified via PCR. Primer pairs were designed from high coverage areas of the sequence assemblies to amplify across regions of ambiguity or low coverage (S1 Table); in nearly all cases the expected amplicon size was observed, except for several regions of possible collapsed repeats. The same general method was applied to verify the order and orientation of the JGI contigs and to validate and strengthen assembly in regions of low sequence coverage for the chromosome and the plasmids in the Lucigen contigs (S1 Fig). All PCR validations were optimized using Taq98 DNA polymerase (Lucigen) or KAPA 2G Robust polymerase (Kapa Biosystems). The amplicons were purified by SPRI beads (Beckman), and used as template for Sanger sequencing for comparison to and inclusion in the existing assemblies.

### Genome Annotation

The assembled Y51MC23 genome was annotated using Rapid Annotations using Subsystems Technology (RAST) [26, 27]. Manual annotation and curation of the genome was also performed. Corresponding genes in the IMG database [28] were identified by BLASTp [29] analysis using protein sequences generated in RAST. Signal peptides were determined using SignalP 4.0 [30]. Peptidases were predicted with MEROPS [31, 32] and confirmed by BLASTp [29] analysis of the UniProt database [33, 34]. Metabolic reconstructions were performed using PRIAM [35] and SEED [27] metabolic pathway construction software. Genomic islands were identified using Island Viewer [36, 37] software and the closed Y51MC23 chromosome. For assembly comparison purposes, the permanent draft genome for *T*. *aquaticus* containing 22 contigs (NZ_ABVK02000001 through NZ_ABVK020000022) was assembled into a single contig and annotated using RAST. Likewise, the chromosome of *Thermus scotoductus* SA-01, ATCC 700910 (NC_014974.1) was re-annotated using RAST. Prophage sequences were identified using the program Prophage Finder [38].

The evolutionary history of Y51MC23 was inferred by using the maximum likelihood method based on the Tamura-Nei model [39] on 16S rDNA sequences. Evolutionary analyses were conducted in MEGA5 [40]. Initial tree(s) for the heuristic search were obtained automatically by applying Neighbor-Join and BioNJ algorithms to a matrix of pairwise distances estimated using the Maximum Composite Likelihood (MCL) approach, and then selecting the topology with superior log likelihood value. The tree is drawn to scale, with branch lengths measured in the number of substitutions per site. The analysis involved 18 nucleotide sequences. All positions containing gaps and missing data were eliminated. There were a total of 1184 positions in the final dataset. Accession numbers for 16S rRNA gene sequences are: *T*. *antranikianii* ATCC12462 ^T^, Y18411; *T*. *aquaticus* YT-1^T^, L09663; *T*. *arciformis* strain TH92^T^, EU247889; *T*. *brockianus* ^T^, Z15062; *T*. *caliditerrae* YIM 77925^T^, KC852874; *T*. *igniterrae* ATCC 700962^T^, Y18406; *T*. *oshimai* strain SPS17^T^, Y18416; *M*. *ruber* DSM 1279^T^, Z15059; *T*. *scotoductus* SA-01, EU330195.1; *T*. *scotoductus* ATCC 51532^T^, AF032127; *T*. *scotoductus* K12, NZ_JQLJ01000001.1; *T*. *tengchongensis* YIM 77924^T^, JX112365; *T*. *thermophilus* 33923^T^, X07998; *T*. *thermophilus* HB27, NR_074423.1; *T*. *thermophilus* JL-18, NC_017587.1; and *Thermus* sp. CCB_US3_UF1, CP003126.1.

## Results

*Thermus aquaticus* Y51MC23 is one of a number of novel thermophilic species isolated from 88°C water in the northern outflow channel of Bath hot spring in the Lower Geyser Basin of Yellowstone National Park [41]. The general features of the organism are summarized in Table 1. The temperature of Bath hot spring is 93.5°C at the source, which is the boiling point at the spring's elevation. The pH of the spring is 8.9 with SiO_2_ (244.8 mg/l) and Cl^-^ (297.1 mg/l) the dominant dissolved minerals [42]. Y51MC23 is a gram-negative, rod-shaped organism that grows well between 50°C and 80°C with maximum growth rate at 65°C to 70°C (optimum pH for growth is 7.6). The organism forms yellow colonies on YTP-2 agar, and cells isolated from liquid cultures are yellow. Colonies are catalase positive, forming bubbles when tested with aqueous hydrogen peroxide. Like most *Thermus* species, Y51MC23 grows well on media containing low concentrations of salts (2 to 4 g/l), yeast extract and protein hydrolysates such as tryptone or peptone and it does not grow in standard laboratory media such as Luria broth. Unlike the type strain *T*. *aquaticus* YT-1 [1], Y51MC23 is unable to grow on a minimal medium containing salts and glucose. Y51MC23 is able to grow under both aerobic and anaerobic conditions, in sharp contrast to YT-1, which was reported to be an obligate aerobe [1]. Cellular morphology in liquid culture is complicated and will be discussed in a later section.

**Table 1:** Features of *Thermus aquaticus* Y51MC23.

Property	Term
Strain	*Thermus aquaticus* Y51MC23
Gram stain	Negative
Cell shape	Rod
Motility	Non-motile
Temperature range	50°C to 80°C
Optimum temperature	70°C
Carbon source	Amino acids and proteins
Energy source	Chemoorganotroph
Terminal electron acceptor	O_2_
Habitat	Hot spring
Oxygen	Facultative anaerobe
Biotic relationship	Free living
Pathogenicity	Non-pathogenic
Geographic location	Wyoming, USA
Latitude	44.5376262
Longitude	-110.690383
Altitude	2195 m

**Fig 1:**
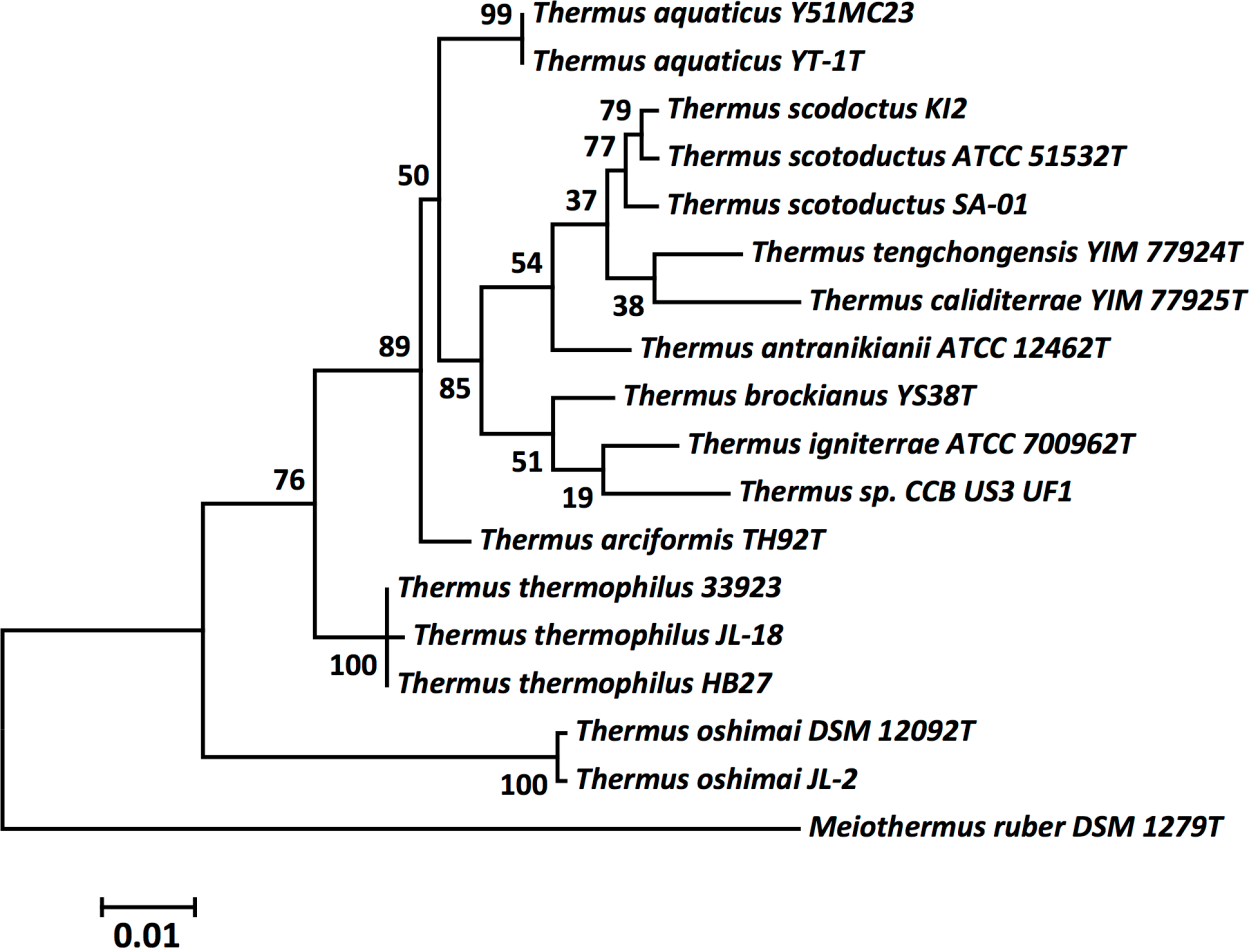
Molecular phylogenetic analysis of *Thermus* species by maximum likelihood method using 16S rRNA gene sequences. The tree with the highest log likelihood (-3496.7463) is shown. The percentage of trees in which the associated taxa clustered together is shown next to the branches.

A phylogenetic tree was constructed to determine the relationship between Y51MC23, Thermus aquaticus YT-1, and other members (including newly-discovered strains) of the *Thermus* family. The phylogeny of Y51MC23 was determined using its 16S rRNA gene sequence, as well as those of the type strains of all validly described *Thermus* sp. The 16S rRNA gene sequences were aligned using MUSCLE [43], pairwise distances were estimated using the Maximum Composite Likelihood (MCL) approach, and initial trees for heuristic search were obtained automatically by applying the Neighbour-Joining method in MEGA 5 [40]. The alignment and heuristic trees were then used to infer the phylogeny using the Maximum Likelihood method based on Tamura-Nei [39]. The phylogenetic tree identifies Y51MC23 as a *T*. *aquaticus* strain (Fig 1), in a separate clade from *T*. *scotoductus*, *T*. *thermophilus* and *T*. *oshimai*. The 16S rRNA gene results are also consistent with the analysis of 1,441 orthologous proteins from the currently sequenced *Thermus* genomes [44].

### Closing and Finishing the *Thermus aquaticus* Y51MC23 genome

*Thermus aquaticus* Y51MC23 was re-sequenced by Illumina chemistry using a 300–400 bp ‘fragment” library and an 8 kb large insert mate pair library, as described in the methods section. Using the fragment library sequence data only, the genome assembled into 82 contigs >1 kb (82 gaps with 164 termini, data not shown). Bioinformatic analysis tabulates 44 repetitive elements > 500 bp in length (discussed in detail later), but only two of them were located internally to the contig termini. These repetitive sequences confound *de novo* assembly programs, causing 84 of 164 contig terminations (51%) in the fragment assembly of *T*. *aquaticus* (data not shown). The failure to extend and join contigs during *de novo* assembly from fragment sequencing reads is well known [45]. Information that extends beyond the repeat length is required to join contigs in the correct orientation and order. An 8 kb “jumping library”, or mate pair library was constructed to span these repeats as described in the methods section. A new genome assembly was computed using the fragment plus 8 kb mate pair library, scaffolding the 82 contigs into a single chromosome assembly and four plasmids. To ensure the accuracy of the closed assembly and to finish several gaps caused by problematic GC rich stretches, eighteen pairs of PCR primers were designed and used to cross areas of uncertainty (S1 Table). The gaps were successfully amplified (S1 Fig) and the amplicons were sequenced by Sanger chemistry to finish the complete genome presented here.

Additional manual curation of the draft genome (http://www.ncbi.nlm.nih.gov/nuccore/218297404) was performed to systematically compare differences within the finished chromosome and its four attendant plasmids described in this work and the draft assembly (S2 Table, where draft genome genes are identified by their UniProt designations of TaqDRAFT_3002 through TaqDRAFT_5541). The manual curation revealed 35 additional annotated genes not present in the draft genome. While most of the newly identified genes are single genes, an insert of six genes (genes 883 through 888) was found in the chromosome. The comparison of the two assemblies also identified 42 genes identified in the IMG (JGI) annotation, but missed by the RAST annotation. The identities of these 42 genes were confirmed and the genes were added to the annotation. The original sequencing effort from 2008 introduced mistakes that caused 27 frameshift errors, resulting in single genes being split into two separate protein fragments. Correction of these frameshift errors removed 27 genes from the assembly. Elimination of partial and mis-assembled genes at the ends of the twenty-two contigs in the draft assembly removed 17 additional genes, and elimination of questionable small open reading frames removed another 20 genes. Finally, the DNA sequences of 12 genes in the draft genome were not present in the final assembly, reducing the number of actual genes in the draft assembly to 2309 in the final assembly. The complete alignment of the final assembly versus the draft genome is shown in S2 Table.

All contigs from the draft assembly were incorporated intact into either the chromosome or one of the four plasmids. A synteny plot was constructed to examine the location of the genes from the draft assembly within the closed chromosome (data not shown). The presence of long stretches of homology in the synteny plot shows that the genes within the contigs of the draft genome were assembled in the correct order, but the contigs themselves could not be assembled. The partial and mis-assembled genes at the ends of the twenty-two contigs prevented recognition of the correct order of these contigs and their assembly in the draft genome from 2008. The use of a mate pair library greater than 5 kb was required to accurately close the genome (data not shown).

**Fig 2:**
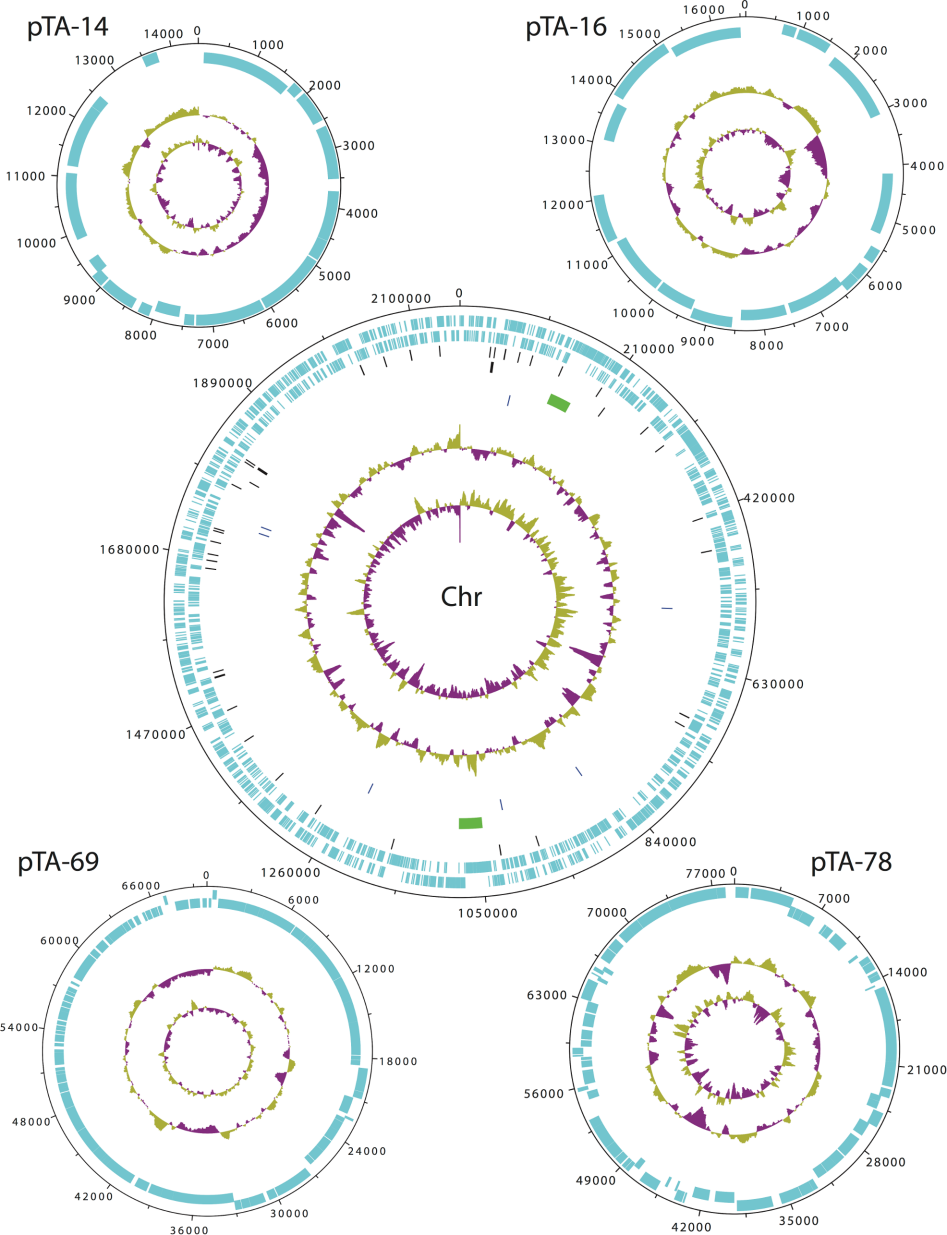
Features of the *Thermus aquaticus* Y51MC23 chromosome and plasmids. Tracks from outside to inside: CDs forward strand, CDs reverse strand, tRNA genes, rRNA genes, prophage, CRISPRs, GC plot, and GC skew. Prepared using DNA Plotter software [46].

**Fig 3:**
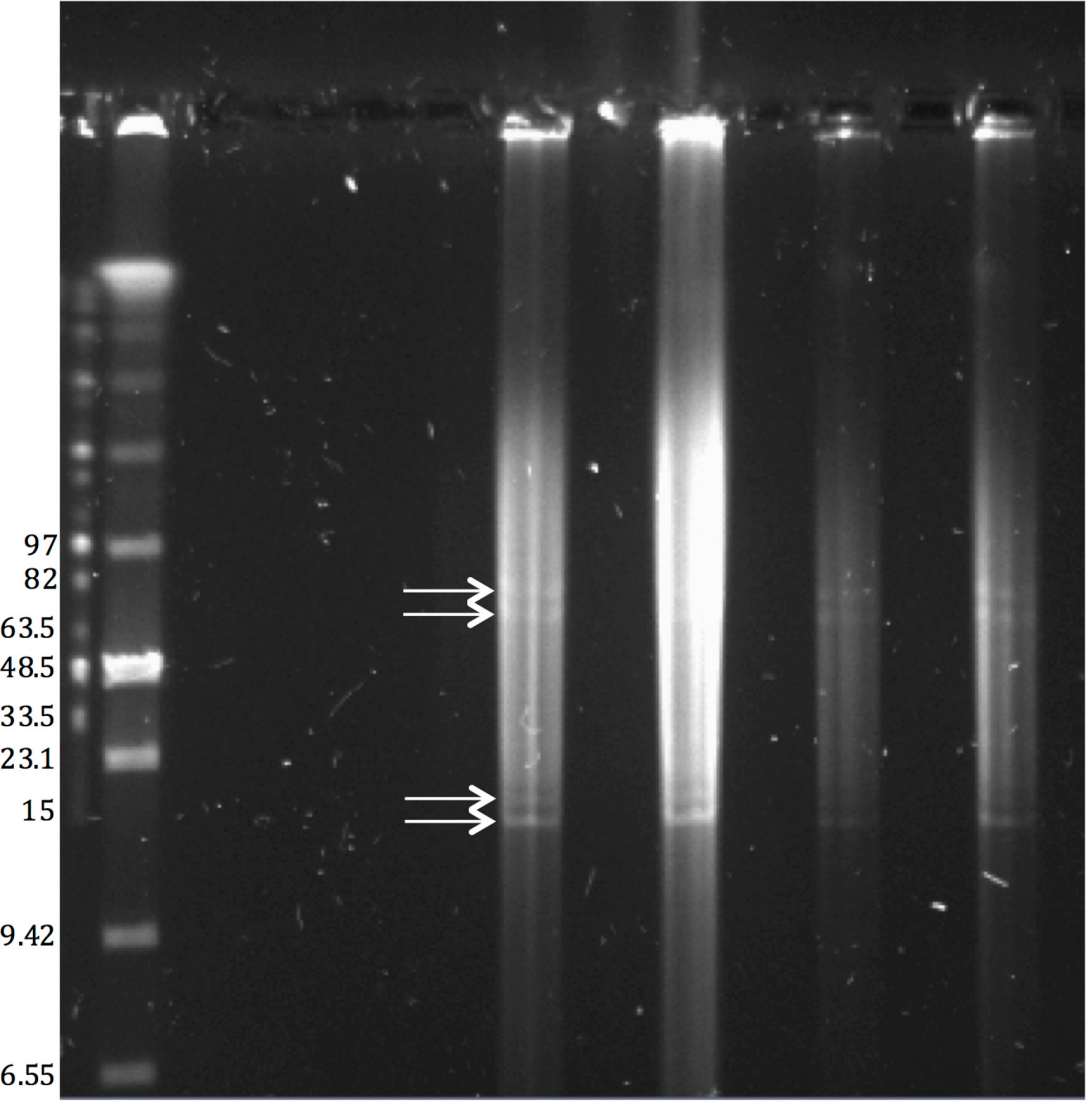
Identification of four *Thermus aquaticus* Y51MC23 plasmids by PFGE. Genomic DNA extracts showed a distinct pulsed field gel electrophoresis banding pattern whose sizes closely match 4 contigs with specific coverage depths that do not map to the primary chromosomal contig. The sizes of these bands (arrows) match actual contig lengths from the finished genome assembly (14.4, 16.6, 69.9, and 78.7 kb respectively), suggesting that the material in the bands is of linear form. The native form of these putative plasmids is presumed to be circular.

The annotation results of the final, closed genome assembly described here are shown in Table 2 and the chromosome plus four plasmids map [46] is shown in Fig 2. The closed genome contains an additional 448 DNA bases not found in the draft sequence analysis. The number of contigs has been reduced from twenty-two to five, consisting of one chromosome and four plasmids (Table 2). The presence of the four plasmids was confirmed by PFGE (Fig 3). The 5S and 23S rRNA genes occur in two clusters that are 382 kb apart, and the two 16S genes are separated from these clusters by an additional 172kb and 19kb. This rRNA content differs from the draft assembly, in which three partial 23S genes were called (all at the ends of contigs), plus two complete 5S genes and one complete 16S gene

**Table 2:** *Thermus aquaticus* Y51MC23 genome summary.

	Chromosome	pTA14	pTA16	pTA69	pTA78
**GenBank Accession**	CP010822	CP010823	CP0108224	CP010825	CP010826
**DNA, total bases**	2158963	14448	16597	69906	78727
**Contigs**	1	1	1	1	1
**G+C**	68.0%	64.0%	67.2%	69.1%	66.3%
**Protein coding genes**	2309	21	20	92	78
**tRNA genes**	50				
**5S rRNA genes**	2				
**16S rRNA genes**	2				
**23S rRNA genes**	2				

### Comparison of Y51MC23 to Other Thermus Species

The RAST annotations of Y51MC23 (**Taq**), *T*. *scotoductus* SA-01 (**Tsc**), *T*. *thermophilus* HB8 (**Tth HB8**) and *T*. *thermophilus* HB27 (**Tth HB27**) were used to prevent differences in annotation software from influencing genome comparisons. The summary of the annotations (Table 3) shows that the four organisms have similar genome sizes and similar numbers of genes within the individual subsystems. (The results from the RAST annotations may not agree with published annotation results obtained using other annotation software.)

**Table 3:** Comparison of RAST annotations of *Thermus* chromosomes.

Subsystem Feature Count	Taq	Tsc	Tth HB8	Tth HB27
Total coding sequences	2309	2422	2240	2233
Cofactors, vitamins, prosthetic groups, pigments	226	188	243	243
Cell wall and capsule	56	52	50	60
Virulence, disease and defense	35	35	43	39
Potassium metabolism	7	6	17	17
Miscellaneous	29	32	32	32
Phages, prophages, transposable elements, plasmids	6	1	0	0
Membrane transport	75	94	74	70
Iron acquisition and metabolism	4	3	11	12
RNA metabolism	109	116	123	146
Nucleosides and nucleotides	77	83	86	82
Protein metabolism	227	225	213	212
Cell division and cell cycle	35	32	33	35
Motility and chemotaxis	2	2	1	2
Regulation and cell signaling	15	14	24	20
Secondary metabolism	5	5	5	5
DNA metabolism	82	77	93	63
Fatty acids, lipids, and isoprenoids	91	89	98	82
Nitrogen metabolism	5	18	4	0
Dormancy and sporulation	2	2	2	2
Respiration	79	102	89	82
Stress response	38	41	38	36
Metabolism of aromatic compounds	7	13	17	3
Amino acids and derivatives	281	288	273	258
Sulfur metabolism	14	18	19	16
Phosphorus metabolism	24	31	26	29
Carbohydrates	210	231	245	202

Abbreviations: Taq, *T*. *aquaticus* Y51MC23; Tsc, *T*. *scotoductus* SA-01; Tth HB8, *T*. *thermophilus* HB8; Tth HB27, *T*. *thermophilus* HB27.

An in-depth comparison was carried out between the genomes of Y51MC23 and *T*. *scotoductus* SA-01, the closest sequenced relative of Y51MC23. Ninety predicted proteins with >95% identity in each species were identified. As expected, most of these ninety predicted proteins are highly conserved, including 31 predicted ribosomal and translation proteins as well as proteins predicted to be involved in electron transport and ATP generation. Other highly conserved predicted proteins are heat and cold shock proteins, a predicted amino acid transporter cluster, a predicted catalase and an unusual SpoVS-related protein. Y51MC23 possesses 369 annotated proteins not present in *T*. *scotoductus* SA-01. These unique (<10% identity) proteins of Y51MC23 have a variety of predicted functions. Among the 369 genes, there are two large inserts of predicted prophage genes and multiple predicted phage defense CRISPR protein clusters. A pathway for production of phytoene-based pigment is predicted in Y51MC23, but not SA-01, in agreement with the published report on SA-01 [10]. A pathway for production of corrinoid-type molecules is also predicted in Y51MC23, but not SA-01. Finally, over 280 hypothetical or putative proteins with unknown functions are predicted t in Y51MC23, but not SA-01. The proteins unique to *T*. *scotoductus* SA-01 are associated with genomic islands that are predicted to confer nitrate reduction, aromatic degradation, and metal utilization abilities [10].

**Fig 4:**
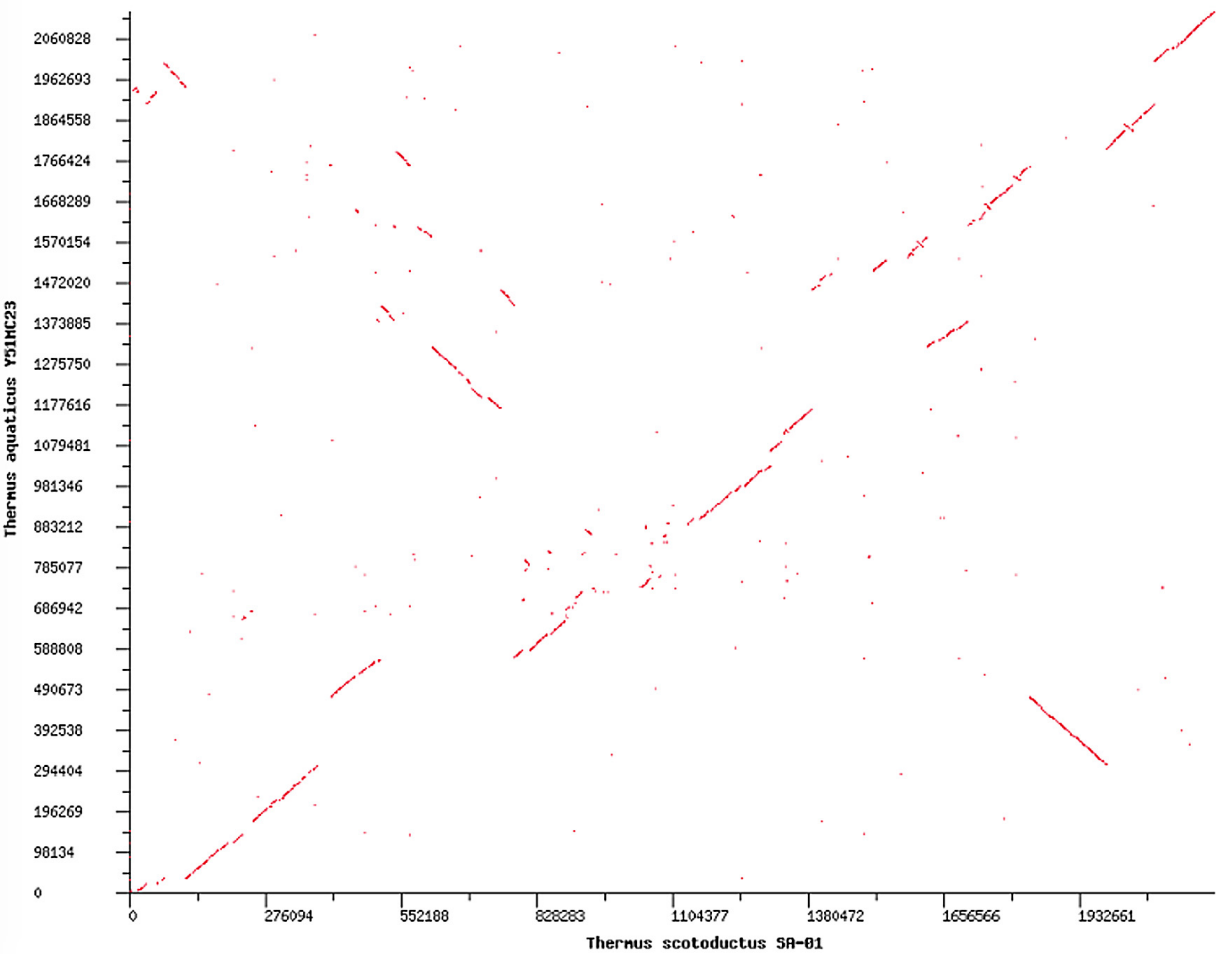
Synteny plot of the closed and finished *T*. *aquaticus* Y51MC23 genome versus *T*. *scotoductus* SA-01.

The genome of *T*. *scotoductus* SA-01 has been labeled hyperplastic [10] because of large differences seen when compared to the genomes of *T*. *thermophilus* HB8 and HB27. Sequence-based comparison of Y51MC23 to *T*. *scotoductus* SA-01, the phylogenetically-closest sequenced species, revealed a different picture. The synteny plot comparison of these two species shows a high degree of synteny throughout the genomes (Fig 4) suggesting that regions of relative stability exists between certain *Thermus* species. Sequence-based comparison of Y51MC23 to *T*. *thermophilus* strains shows fewer and smaller regions of synteny, as expected from the greater distance on the phylogenetic tree (Fig 1).

Genomic islands have been implicated in rearrangements [44] and acquisition of new metabolic capabilities [10] in *Thermus* species. Analysis of the Y51MC23 genome using sequence composition-based approaches, such as SIGI-HMM and IslandPath-DIMOB, and the comparative genomics approach IslandPick [36] showed only two small potential genomic islands (data not shown). The first potential island (7473 bp) contains eight genes (1072 through 1079), and the second potential island (7678 bp), six genes (2195 through 2200). Neither island codes for an identifiable metabolic pathway. BLASTn analyses show both regions are highly conserved in other *Thermus* species such as *T*. *scotoductus*, *T*. *thermophilus*, *T*. *oshimai*, and *Thermus* sp. CCB_US3_UF1, with 50% to 80% coverage and 75% to 85% identity. No significant (>2% coverage) hits to non-*Thermus* sequences were found, supporting the hypothesis that these genes were not acquired from organisms outside the genus.

### Insights from the *Thermus aquaticus* Y51MC23 genome

#### Transport functions

Y51MC23 possesses genes encoding 35 predicted membrane transporter systems, significantly more than the 22 predicted transporter systems reported in *T*. *scotoductus* SA-01 [10]. Like *T*. *scotoductus*, Y51MC23 appears to rely primarily on thirty-one ABC (ATP-binding cassette protein) transporter systems (Table 4) that couple ATP hydrolysis to transport of nutrients from the medium. ABC transporters typically contain four components, two integral membrane proteins and two cytoplasmic proteins [47], though ABC transporters that utilize fewer and more varied components (ECF transporters) have also been described [48]. Of the thirty-one predicted ABC transporter systems, the highest number, thirteen, are predicted to transport peptides and amino acids, indicating Y51MC23 may utilize amino acids as its primary energy source. Y51MC23 possesses genes that could potentially encode three ABC transporter systems for sugars, two are predicted to transport maltose and maltodextrins, and one to transport inositol. Y51MC23 possesses genes that could potentially encode for five ABC transporter systems for essential metal ions, one each for molybdenum, heavy metals, and copper and two for iron. Other potential ABC transporter systems are annotated as transporters for amines such as spermidine, putrescine, and taurine; thiamine; heme; and glycerol phosphate. Most of the potential ABC transporter system clusters contain either three or four genes: two for membrane components and one or two for permease components. The annotated thiamine, cytochrome C biogenesis, and glycerol-phosphate transport genes may be part of ECF transporters. In addition to the potential ABC transporters, Y51MC23 possess two predicted tripartite ATP-independent periplasmic (TRAP) transporters that utilize an ion gradient to transport four carbon dicarboxylic acids, and a predicted PacL-type ATPase cation transporter that may transport potassium, calcium or heavy metals such as lead, mercury, cadmium, or zinc. An annotated calcium-sodium antiporter is also present. There are no genes that could potentially encode three-component phosphotransferase system (PTS) transporter systems that use phosphoenolpyruvate to transport sugars into the cell and phosphorylate them.

**Table 4:** Predicted Transporter systems present in the *T*. *aquaticus* Y51MC23 genome.

Genes	Transporter Type	Annotated Substrate
36–38	ABC	Maltose-maltodextrin
104–106	ABC	Unknown
240–242	ABC	Molybdenum
311–312	ABC	Thiamine
504–505	ABC	Cytochrome C biogenesis
571–573	ABC	Oligopeptide
574–578	ABC	Branch chain amino acids
589–591	TRAP	C4-dicarboxylic acids
634–637	ABC	Branch chain amino acids /leucine
704	ATPase	Cations
706	Antiporter	Calcium-sodium
804–807	ABC	Inositol
816–818	ABC	Dipeptides
850–852	ABC	Heme
877–878	ABC	Branch chain amino acids
880–882	ABC	Branch chain amino acids
902–906	ABC	Branch chain amino acids
913–915	ABC	Glycerol phosphate
925–928, 930	ABC	Branch chain amino acids
965–968	ABC	Branch chain amino acids
1024–1026	ABC	Taurine
1120	ABC	Glycerol phosphate
1231–1234	ABC	Iron citrate
1258–1262	ABC	Amino acids-lysine, arginine, ornithine
1297–1299	ABC	Unknown
1397–1400	ABC	Spermidine, putrescine
1503–1505	ABC	Copper
1808–1812	ABC	Oligopeptides
1843	ABC	Amino acids-methionine
2031–2033	ABC	Maltose-maltodextrin
2042–2043	ABC	Branch chain amino acids
2079	ABC	Ferric iron
2108–2110	TRAP	C4-dicarboxylic acids
2178–2179	ABC	Glycerol phosphate
2213	ABC	Iron

#### Sugar Metabolism and Biosynthesis

Based on the SEED metabolic reconstruction [27], Y51MC23 is predicted to interconvert and utilize glucose, fructose, galactose and mannose. However, based on the annotation, it is unclear if transport systems for galactose, mannose and fructose exist, and the lack of these transport systems may limit the organism’s ability to utilize galactose, mannose and fructose. Of the hexose oligosaccharides and polysaccharides, Y51MC23 is predicted to be able to only utilize maltose, maltodextrins, and oligosaccharides derived from starch. The organism possesses three genes predicted to encode glycoside hydrolase family 13 proteins (GH13) and one GH57 amylopullulanase [49]. The GH 57 amylopullulanase gene (2070) codes for a predicted signal peptide[30], suggesting the protein is secreted into the medium where it may generate soluble malto-oligosaccharides from starch. These malto-oligosaccharides may then be transported by two predicted ABC transporters coded by genes 36, 37 and 38, and 2031, 2032 and 2033. The three predicted GH13 genes (388, 408, and 2034) do not code for signal peptides and these three amylases appear to act only on intracellular substrates, either malto-oligosaccharides or storage polysaccharides, converting them to glucose for utilization. There are no annotated genes coding for secreted galactosidases, invertases, cellulases, or mannanases, indicating these substrates cannot be utilized by Y51MC23. The SEED metabolic reconstruction also indicates that Y51MC23 lacks the genes needed to utilize commonly occurring pentose sugars such as xylose, xylulose, or arabinose. A lack of genes coding for the necessary pathway enzymes also prevents utilization of inositol, inositol phosphate, sugar alcohols, glucuronate and other sugar acids. The genes predicted to code for a complete pentose phosphate pathway are present, predicting the ability to produce ribose from fructose.

#### Energy Generation

Y51MC23 is predicted to possess a complete Embden-Meyerhof-Parnas (glycolysis) pathway for the utilization of glucose, fructose and related sugars. Based on the SEED metabolic reconstruction, pyruvate formed in glycolysis may be converted to acetate, lactate and ethanol under anaerobic conditions. While Y51MC23 does not possess genes coding for an annotated NADH-utilizing lactate dehydrogenases for conversion of pyruvate to lactate, pyruvate may be converted to lactate via a predicted cytochrome c-dependent D-lactate dehydrogenase (gene 317) and a predicted three-component L-lactate dehydrogenase coupled to an Fe-S oxidoreductase (genes 548, 549, 550). Ethanol may be produced from pyruvate by a predicted alcohol dehydrogenase (gene 1813).

Under aerobic conditions the pyruvate produced in glycolysis is predicted to be utilized via a complete citric acid (TCA) cycle, with ATP generated through oxidative phosphorylation. Y51MC23 possesses a predicted NADH ubiquinone oxidoreductase complex coded by genes 1536 through 1549 (chains A through N) and gene 1789 (NADH-quinone oxidoreductase chain 15). Genes 1497 through 1500 are predicted to code for components of a succinate dehydrogenase. Y51MC23 appears to have genes coding for two separate cytochrome C oxidase complexes (genes 713–714 and 2163–2164) as well as an ubiquinol cytochrome C oxidoreductase complex (genes 2181–2184). Unlike other *Thermus* species [19], Y51MC23 appears unable to utilize nitrate as a terminal electron acceptor, because the strain contains no annotated gene cluster for the reduction of nitrate to nitrous oxide. This was confirmed by fermentation studies, where addition of nitrate did not stimulate growth of Y51MC23 under anaerobic conditions.

ATP appears to be generated using a V-type ATP synthase V complex. V-type ATPases have been reported in *T*. *aquaticus* YT-1 as well as *T*. *thermophilus* HB27, while other *Thermus* species possess the more typical bacterial F-type [50]. The genes coding for the predicted ATP synthase are located downstream from the NADH ubiquinone oxidoreductase genes. The ATPase genes, 1552 through 1560, code for (in order) ATPase subunits D, B, F, A, C, E, K, I, G.

#### Amino Acid Metabolism and Biosynthesis

Y51MC23 may utilize a combination of secreted, membrane bound, and cytosolic proteases and peptidases for converting external proteins and peptides into free amino acids. In contrast to the predicted single secreted enzyme for oligosaccharide degradation (the GH 57 amylopullulanase), Y51MC23 possesses thirteen genes predicted to code for secreted peptidases/proteases. Among the thirteen secreted proteins, the annotations predict three subtilisin-like proteases, two chymotrypsin-like proteases, two metalloproteases, and a carboxypeptidase. The two predicted chymotrypsin-like proteases (genes 1448 and 1635) have annotated PDZ domains, that may increase the thermostability of these proteases [51]. The peptides and amino acids generated by these enzymes may be transported into the cytoplasm via the thirteen predicted peptide and amino acid ABC transporter systems described above. Once inside, seventeen predicted soluble peptidases and two predicted membrane-bound peptidases may hydrolyze the peptides to free amino acids for metabolism and reuse.

Y51MC23 recycles misfolded proteins into amino acids using two ATP-dependent pathways. The organism possesses genes predicted to code for a two-subunit 20S proteasome system (gene 769, HslV and gene 770 HslU) [52, 53] as well as three annotated Lon proteases (genes 332, 737, and 1458) [54]. There are no genes predicted to code for recycling transglutaminase/proteases annotated in the genome. Y51MC23 also possesses genes predicted to code for an ATP-dependent TldE/TldD proteolytic complex (genes 1447 and 1448) with an unknown function.

Based on the metabolic reconstruction, Y51MC23 appears to be able to metabolize sixteen of the twenty amino acids. Based on genes present, pathways for degradation of Glu, Gln, Asp, Asn, Ala, Gly, Ser, Thr, Met, Cys, Val, Leu, Ile, Arg, Pro, and His are predicted, while no predicted pathways exist for degradation of Lys, Trp, Tyr, Phe. Under aerobic conditions, the degradation products of these amino acids appears to funnel predominantly into the citric acid cycle and generate ATP via oxidative phosphorylation. While Y51MC23 is able to grow on amino acids under anaerobic conditions, it is unclear how ATP generation is coupled to amino acid degradation.

The metabolic reconstruction indicates that Y51MC23 possesses all genes necessary for the biosynthesis of all twenty amino acids via conventional microbial pathways. Among the amino acids with multiple known biosynthetic pathways, lysine is predicted to be synthesized via the N-2 acetyl-L-aminoadipate pathway and cysteine is predicted to be synthesized from acetyl serine and H_2_S. Genes within biosynthetic pathways are not organized into a single operon controlled by regulatory units. The thirteen Y51MC23 tryptophan biosynthesis genes are scattered throughout the genome (genes 520, 521, 522, 1058, 1353, 1354, 1447, 1733, 1734, 2080, 2187, 2188, and 2189), unlike the tryptophan operon in *E*. *coli* [55] or thirteen-gene operon (GY4MC1_1353 through GY4MC1_1364) for tryptophan biosynthesis present in *Geobacillus thermoglucosidasius* Y4.1MC1, a Gram-positive organism isolated from the same hot spring as Y51MC23 [41].

#### Biosynthesis of Vitamins, Cofactors, and Pigments

Y51MC23 possesses genes potentially encoding biosynthetic pathways for production of three porphyrin products, heme, siroheme, and corrinoids. In addition to the predicted heme biosynthetic pathway, Y51MC23 also possesses genes potentially encoding an ABC transporter cluster for uptake of heme (genes 850, 851, and 852). The predicted biosynthetic pathway for corrinoid biosynthesis is similar to that found in *T*. *thermophilus* HB8 megaplasmid pTT27. In Y51MC23, the predicted pathway is primarily coded by an eleven gene cluster (genes 976 through 986) that is syntenous to pTT27 genes TTHB051 through TTHB060. Remaining orthologs to the pTT27 pathway are scattered through the Y51MC23 chromosome. Y51MC23 encodes genes for the pathways involved in biosynthesis of riboflavin, nicotinate/NAD, and folate. Genes coding for pathways involved in biosynthesis of thiamine, B6, pantothenate, and biotin are absent, leading to the growth requirement for yeast extract.

Like most *Thermus* species, Y51MC23 cells are yellow, the result of carotenoid biosynthesis [56]. Y51MC23 possesses a chromosomal gene cluster for phytoene biosynthesis (genes 1210 through 1222) similar to the phytoene gene cluster in *T*. *thermophilus* HB8 megaplasmid pTT27 (genes TTHB098 through TTHB110) [57]. The two clusters show the same organization, with the pTT27 cluster containing an additional annotated plasmid stability protein (gene TTHB108) within the cluster. Light regulates carotenoid biosynthesis via a LitR-dependent regulatory protein in the Crp/Fnr family (gene 1211) similar to that observed in *T*. *thermophilus* [58], and the light is detected via a proteorhodopsin (gene 1599) [59].

#### Plasmids

Y51MC23 possesses four plasmids. The two smaller plasmids, pTA14 and pTA16, contain 21 and 20 genes respectively. The genes predominantly code for hypothetical proteins with no significant BLAST hits to proteins with known functions. Plasmid pTA78 contains 78 annotated genes. Among the genes of interest in this plasmid are clusters predicted to encode a heme transport and utilization system (pTA78-27 through 31), an aerobic-type, class 1a ribonucleotide reductase (pTA78-34 and pTA78-35), and chromosome partitioning proteins ParA and ParB (pTA78-74 and pTA78-75). The plasmid also contains two partial copies of the pilT gene (pTA78-41 and pTA78-53, 137 and 138 a.a. respectively), which may allow integration of the plasmid into the two chromosomal copies of pilT (genes 50 and 210). The entire heme transport gene cluster is also present in the chromosome of Thermus sp. CCB_US3_UF1 (accession CP003126, nt. 228390–224668), the megaplasmid pTT27 of *Thermus thermophilus* HB8 and HB27 (accession AP008227, nt. 223,442–227,102 and accession AE017222, nt. 176,277–179,767, respectively) and the megaplasmid pTHEOS01 of *Thermus oshimai* (accession CP003250, nt. 156812–160493).

The second largest plasmid, pTA69, contains 92 annotated genes, and appears to code for a *Thermus* conjugation system. The conjugation system consists of relaxasome [60] and transferosome components. The transferosome is made of genes predicted to encode a type IV secretion system (T4SS) [61]. Because of the diverse structures of the protein components of relaxasomes and transferosomes, the function of each plasmid protein cannot be definitively ascertained. Based on structural and sequence homologies, the plasmid encodes predicted orthologs of relaxosome proteins including primase (pTA69-32), relaxase (pTA69-45), ssDNA binding protein (pTA69-70) and ds break repair protein (pTA69-62). The plasmid encodes identifiable orthologs of T4SS components including virB1 (pTA69-13), virB2 (pTA69-22), virB3 (pTA69-27), virB4 (pTA69-20), virB5 (pTA69-22), virB6 (pTA69-18), virB7 (pTA69-26), virB9 (pTA69-30), virB10 (pTA69-31), virB11 (pTA69-34), VirD4 protein (pTA69-16), and conjugal transfer protein TraD (pTA69-19) as well as additional annotated membrane proteins that may be components of the secretion system [62]. Orthologs of these genes are present in the *Thermus thermophilus* JL-18 plasmid pTTJL1802 (accession CP003254) and SGO,5JP17-16 plasmid pTHTHE1601 (accession CP002778). The pTA-69 plasmid also contains genes for two predicted nucleases (pTA69-41and pTA69-77), a Type I restriction-modification system (pTA69-42), a serine protease (pTA69-46), peptidase (pTA69-3), chromosome partitioning proteins ParA and ParB (pTA69-43 and pTA69-44), and RNA polymerase sigma-70 factor (pTA69-91).

*Thermus thermophilus* strains HB8 and HB27 both possess “megaplasmids” that contain over 200 kb of DNA. The *T*. *thermophilus* HB8 plasmid pTT27 (NC_006462) possesses 228 RAST-annotated genes. Of these 228 genes, 133 have orthologs on the Y51MC23 chromosome, and 95 have no orthologs. Orthologs of the genes of the HB8 megaplasmid include the cobalamin synthesis cluster (TT_P001 through TT_P023), which is present on the Y51MC23 chromosome (0832 through 0839 and 0976 through 0985). The HB8 megaplasmid hemin transport cluster (TT_P175 through TT_P179) also has a Y51MC23 chromosomal ortholog (0849 through 0852), which differs from the heme transport/utilization cluster on plasmid pTA-78. Orthologs to the genes of the phytoene synthetic cluster on the HB8 megaplasmid (TT_P101 through TT_P111) are also found in the Y51MC23 chromosome (1213 through 1222). Of the 95 megaplasmid genes with no orthologs in the Y51MC23 chromosome, 58 code for hypothetical proteins. Other genes with no orthologs in the Y51MC23 chromosome are four annotated *beta*-galactosidases and *one* alpha-glucosidase. The ability to map orthologs of over half of the megaplasmid genes and clusters to the Y51MC23 chromosome (often with a high degree of synteny suggests that Y51MC23 may have stably incorporated large sections of a megaplasmid into its chromosome. Further work is needed to establish if this chromosomal incorporation is a rare or common event in *Thermus* species.

#### Prophage

**Fig 5:**
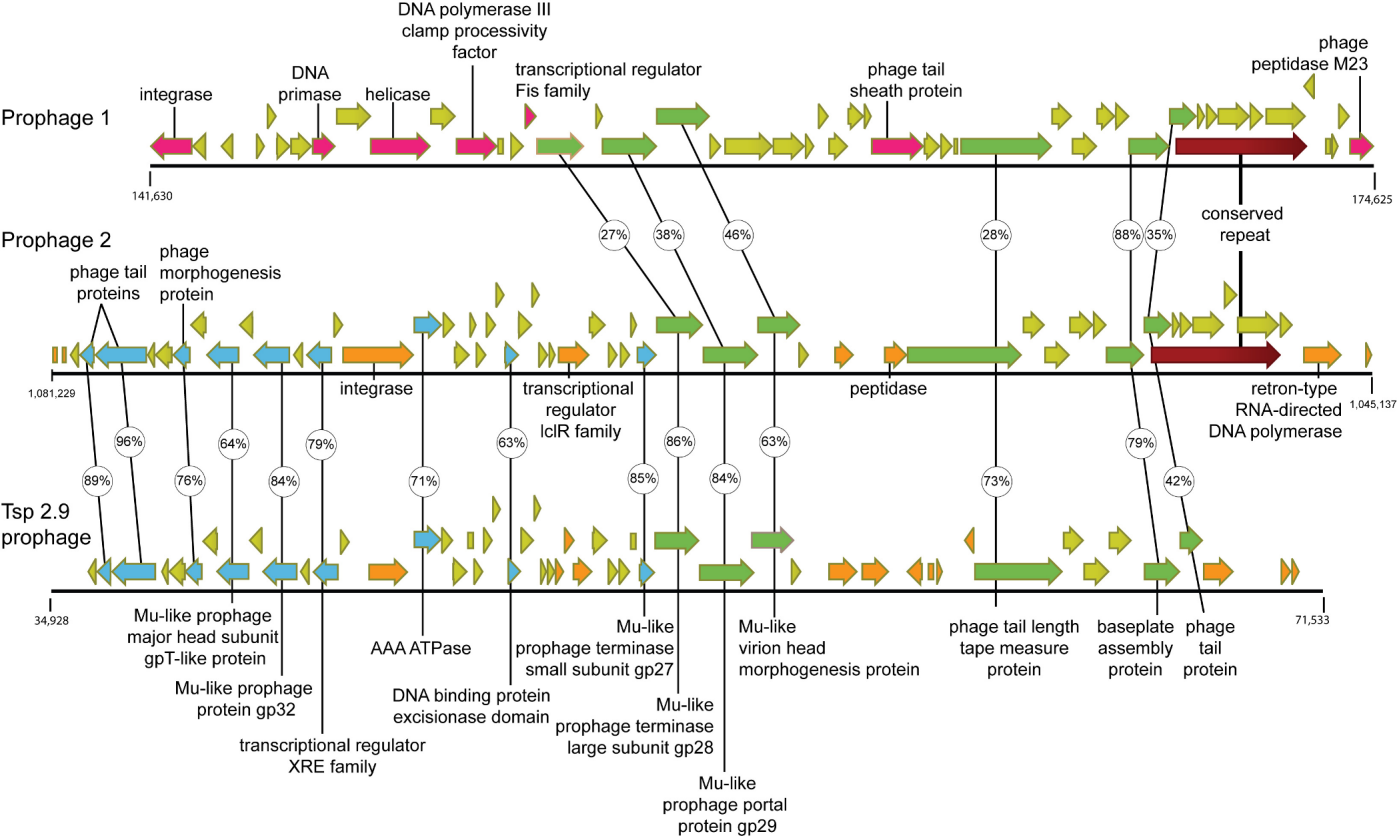
Diagram of Y51MC23 prophage 1 and 2 versus *Thermus* sp. 2.9 prophage synteny. Annotated ORFs are shown as block arrows for *Thermus aquaticus* prophage 1 (top), prophage 2 (center), and *Thermus sp*. *2*.*9* (bottom). Gold indicates hypothetical ORFs; Green indicates ORFs shared between all three prophage; magenta indicates ORFs specific to prophage 1; blue indicates ORFs shared between prophage 2 and TSP2.9 prophage; orange indicates regions that differ between prophage 2 and TSP2.9 prophage. The 3,652 bp region of nucleic acid identity between prophage 1 and 2 is indicated in brick-red color. Amino acid identity between pairs of encoded proteins are indicated in small circles as determined by blastp.

Numerous lytic phages that infect *Thermus* species have been described [63–71], but little is known about temperate bacteriophages and their putative prophage elements. Y51MC23 is unusual among the sequenced *Thermus* species in possessing two large prophages. *Thermus* sp. RL [20], *Thermus* sp. CCB_US3_UF1 [21], and *Thermus* sp. 2.9 [22] each have one prophage, while none of the other *Thermus* genomes appear to have any. Y51MC23 prophage 1 is 32,996 bp (positions 141,630–174,625) and prophage 2 is 36,093 bp (positions 1,045,137–1,081,229). Prophage 1 contains 48 annotated genes (23 hypothetical) whereas prophage 2 contains 55 (28 hypothetical) (Fig 5). The architecture of both elements is similar to many known phage where structural and replication associated genes are arranged together in modules. Both prophage contained multiple structural genes for predicted head/capsid proteins (174, 1175), tail (177, 180, 185, 190, 194, 1138, 1180, 1181), baseplate (118, 189, 1139), and virion processing genes for terminase packaging (168, 1150, 1151), tape measure (184, 1144), baseplate assembly (188, 189), lysozyme (188, 1140), and portal/morphogenesis proteins (168, 171, 1148, 1149, 1177). Many of these are annotated as Mu-like; bacteriophage Mu is a dsDNA temperate phage that uses DNA-based transposition in its lysogenic cycle. Predicted replication and repair genes were more distinct between the two prophage elements. Prophage 1 contains annotated genes for a predicted DNA primase (160), helicase (162), DNA polymerase III beta clamp processivity factor (164), and Holliday junction resolvase (166), whereas prophage 2 only contains an annotated gene for a predicted RNA-directed DNA polymerase (1131).

The two Y51MC23 prophages are unique across most of their length, but they do share 99.6% identity (3528/3562 identical) across 3,562 bp (prophage 1 positions 169,281–172,822; prophage 2 positions 1,047,643–1,051,184) (Fig 5). The region of 99.6% homology is 1803 or 2506 bp from the boundaries of the prophage elements. A novel RNA-directed DNA polymerase (1131) near the terminal boundary for prophage 2 could explain the apparent duplication of five genes in two separate prophage separated by 874,821 bp in Y51MC23. The RNA-directed DNA polymerase at locus 1131 is a special type of reverse transcriptase classified as a “diversity generating retroelement” (DGR) [72]. DGRs encode a reverse transcriptase (RT) as well as a template repeat (TR) and a variable repeat (VT) [72], all three of which were identified in Taq Y51MC23. TR1 is located at 1,047,173–1,047,258 (GGAATGGCGGTAACGCGGGGCTCGCCGCGTTGAACCTGCTCAACCCGCGCGGCAACCGGAACTGGAGTGTCGGGGCCCGCCCCGCT), and VR1 is located at 1,047,693–1,047,778 (GGAATGGCGGTGTCGCGGGGCTCGCCGCGTTGTACCTGCTCAACCCGCGCGGCTCCCGGCGCTGGGGTGTCGGGGCCCGCCCCGCT), just upstream of the reverse transcriptase (1,045,973–1,047,016). VR1 is located in the carboxy-terminal end of the putative formylglycine-generating sulfatase enzyme (FGE sulfatase), which is also the case 27% of the time in the other 155 identified DGR elements [72]. The role that FGE sulfatase might play as a target protein for diversity generation is unknown [72].

There are seven *Thermus*-specific bacteriophages whose genome has been determined (phiYS40, TMA, phiOH2, P23-77, P23-45, p74-26, In93), none of which have homology to Y51MC23 prophage 1 or 2 genes. However, there is a significant degree of synteny and homology between Y51MC23 prophage 2 (55 annotated genes) and a prophage embedded in the *Thermus* species 2.9 [22] genome (55 annotated genes) (Fig 5). *Thermus aquaticus* Y51MC23 was isolated from Bath Hot Spring in Yellowstone National Park, whereas *Thermus* species 2.9 (Tsp 2.9) was isolated from a hot spring in Salta, Argentina [22]. Thirty eight genes share homology between the two prophages, ranging from 42–98% identity (Fig 5). The synteny between the two prophages is colinear except for three regions between Taq Y51MC23 1137–1138, 1144–1150, and 1155–1157 (Fig 5). The single gap at Taq Y51MC23 1168–1169 is most likely a sequence error in the Tsp 2.9 genome at QT17_04420, as this gene appears to have a frame shift mistake that would make it part of 4415. Both prophages have identical flanking bacterial genes on both sides of their respective insertion sites: cell division protein gene *mraZ* (gene locus 4350 for Tsp 2.9, 1185 for Taq Y51MC23) on one side, and 50S ribosomal protein L31 (gene locus 4640 for Tsp 2.9, 1129 for Taq Y51MC23) on the other side.

Two more putative prophage remnants can be found from gene locus 773–796 and 2148–2161, which includes a phage associated primase (784), phage recombination protein Bet (785), and a hypothetical protein homolog to DNA repair proteins (783) for the first one, and a conserved hypothetical protein with homology to primase (2151), a bifunctional DNA primase/polymerase family protein (2152) and a nuclease domain protein (2158) for the second one.

#### CRISPR Elements

CRISPR-Cas modules (Clustered regularly interspersed short palindromic repeats-CRISPR associated proteins) comprise the adaptive immune system in many bacteria and archaea. In contrast to the typical single CRISPR locus in most bacterial genomes, Y51MC23 contains 7 definite CRISPR loci (Table 5), similar to the hyperthermophile *Sulfolobus solfataricus*, which contains 6 CRISPR loci [73]. Similar numbers of CRISPR loci were found in organisms isolated from Yellowstone hot springs and sequenced by our group, including *Geobacillus thermoglucosidasius* Y41MC1 (Bath hot spring, 6 CRISPRs) [41], *Geobacillus thermocatenulatus* strains Y412MC52 and Y41MC61(Obsidian Pool, 6 CRISPRs each), and *Geobacillus thermoglucosidasius* C56-YS93 (Obsidian Pool, 6 CRISPRs). Features of the definite CRISPR loci and flanking ORFs (arrows indicate reading frame orientation) are listed below. CRISPR 1 is flanked by *cas2*, *cas1*, and CRISPR-associated *cas02710* (genes 92–94). CRISPR 5 is flanked by *cas2*, CRISPR-associated genes *csd2/csh2*, *csh1*, CRISPR repeat RNA endoribonulease gene *cas6*, CRISPR-associated RecB-family exonuclease gene, and *cas1* (genes 947–954). CRISPR 8 is flanked by CRISPR-associated genes *csm1* through *csm5*, CRISPR-associated *cas02710*, CRISPR repeat RNA endoribonuclease *cas6*, and Exonuclease *sbcC* and *sbcD* (genes 1341–1353). CRISPR 9 and 10 are located very near each other and are separated by CRISPR-associated gene *TM1812* (gene 1849) plus CRISPR-associated RAMPs *cmr1* through *cmr6* (genes 1850–1855).

**Table 5:** CRISPR elements found in *T*. *aquaticus* Y51MC23.

Name	Genomic location	# DRs	Left flank	Right flank
CRISPR 1	81689..82168	7	<-*cas2*	<-*cas1*
CRISPR 4	550600..550809	4	<-hypothetical	<-disulfide bond formation protein B gene
CRISPR 5	869567..871070	23	*cas1*->	Transcriptional regulator *tetR*->
CRISPR 7	1010468..1010734	4	Mobile element gene->	<-hypothetical
CRISPR 8	1232917..1233864	13	<-putative esterase gene	CRISPR-associated *csm1*->
CRISPR 9	1731966..1732521	8	<-3’-5’ oligoribonuclease A gene	CRISPR-associated *TM1812*->
CRISPR 10	1742839..1743245	6	hypothetical->	<-hypothetical

#### Other Repetitive Elements

Overall the genome contains 44 repetitive elements > 500 b in length, the largest of which are the prophage repeats (2 copies, 3544 bp) and rRNA operons (2 copies, 3361 bp). The remaining 40 repeats fall into 12 classes with lengths ranging from 757 bp to 1695 bp, and copy numbers ranging from 2 to 8. The average identity for the 44 repeats is 98.16%, with 16 of the 44 repeats inverted relative to the first copy. Altogether the 44 elements account for 41,282 bp, equal to 1.91% of the genome. The highest copy number elements are mobile element protein WP_003043735.1 (8 copies, 1690 bp) and an ORF with similarity to IS4 family transposase WP_003043670.1 (7 copies, 923 bp). These 44 repeat elements were the predominant cause of contig extension failure during *de novo* assembly from fragment sequencing reads, accounting for 84 of 164 contig termini (51%) in the fragment assembly.

#### Cell Envelope and Cell Shape

A 95 kd protein, SlpA, is primarily responsible for formation of the S-layer in *T*. *thermophilus* HB8 and HB27 [74, 75], and constitutes most of the protein recovered from the cell envelope. Y51MC23 has a SlpA ortholog (gene 2228) with 911 amino acids. Immediately downstream of the *slpA* gene is an annotated glucosamine-6-P synthase gene, an ortholog of the *glmSth* gene found downstream of the S-layer protein gene in *T*. *thermophilus* [76]. The product of the glucosamine-6-P synthase gene is also a component of the cell envelope. Based on SignalP analysis [30], Y51MC23 also possesses a number of genes coding for large, non-catalytic secreted proteins that may be involved in cell envelope structure or formation of multiple cellular forms observed in micrographs. These proteins include the product of gene 1363, coding for a predicted secreted protein of 2665 amino acids, and the products of genes 932, 933, 934 and 935, coding for predicted secreted proteins of 912, 882, 1793, and 1356 amino acids respectively. BLASTp analyses of the sequences of these proteins show they are conserved in members of the *Deinococcus*-*Thermus* phylogenetic group, but not in other organisms.

The Y51MC23 genome codes for a number of proteins that may be involved in generating the unusual morphologies seen in micrographs described below. Y51MC23 possesses two genes predicted to be *lytR_cpsA_psr* family members (genes 206 and 1577). The *lytR_cpsA_psr* family members are putative membrane-bound proteins that contain an additional predicted extracellular domain. The LytR_CpsA family of proteins has been implicated in a variety of functions in other organisms including cell capsule formation in *Streptococcus pneumonia* [77] and regulation of *Bacillus anthracis* cell length through S-layer assembly and attachment of secondary cell wall polysaccharide to peptidoglycan [78]. The two annotated *lytR_cpsA_psr* genes may regulate the formation of single cells versus chains of cells in Y51MC23 and the overall length of the chains. In addition, Y51MC23 possesses annotated *spoVR and spoVS* genes (genes 11651 and 1644) and a prkA gene (gen 1649). In *B*. *subtilis*, spoVR [79] and spoVS [80] genes are involved in the asymmetrical cell differentiation that ultimately leads to spore formation. The prkA gene codes for a serine protein kinase [81] that is also involved in spore formation in *B*. *subtilis* [82], where PrkA accelerated sporulation and the expression of σ^K^ by suppression of Hpr [83]. Y51MC23 possesses a gene coding for an annotated, secreted, stage II sporulation protein D ortholog (SpoIID, gene 406). In *B*. *subtilis*, SpoIID is a membrane-anchored enzyme that degrades peptidoglycan and is required for sporulation [84]. Two predicted cell wall endopeptidases (genes 214 and 1610) may be involved in shape determination. Finally, Y51MC23 possesses a gene coding for a secreted protein (gene 391) that contains two sporulation related domains that bind to peptidoglycan. These domains are found in proteins such as FtsN, DedD, and CwlM that are involved in cell division and spore formation. Taken together, the presence of these proteins suggests a more primitive system with similarities to sporulation in Gram-positive organisms is responsible for the formation of the unique, multiple morphologies seen in the micrographs below.

### Unique Cellular Morphologies

**Fig 6:**
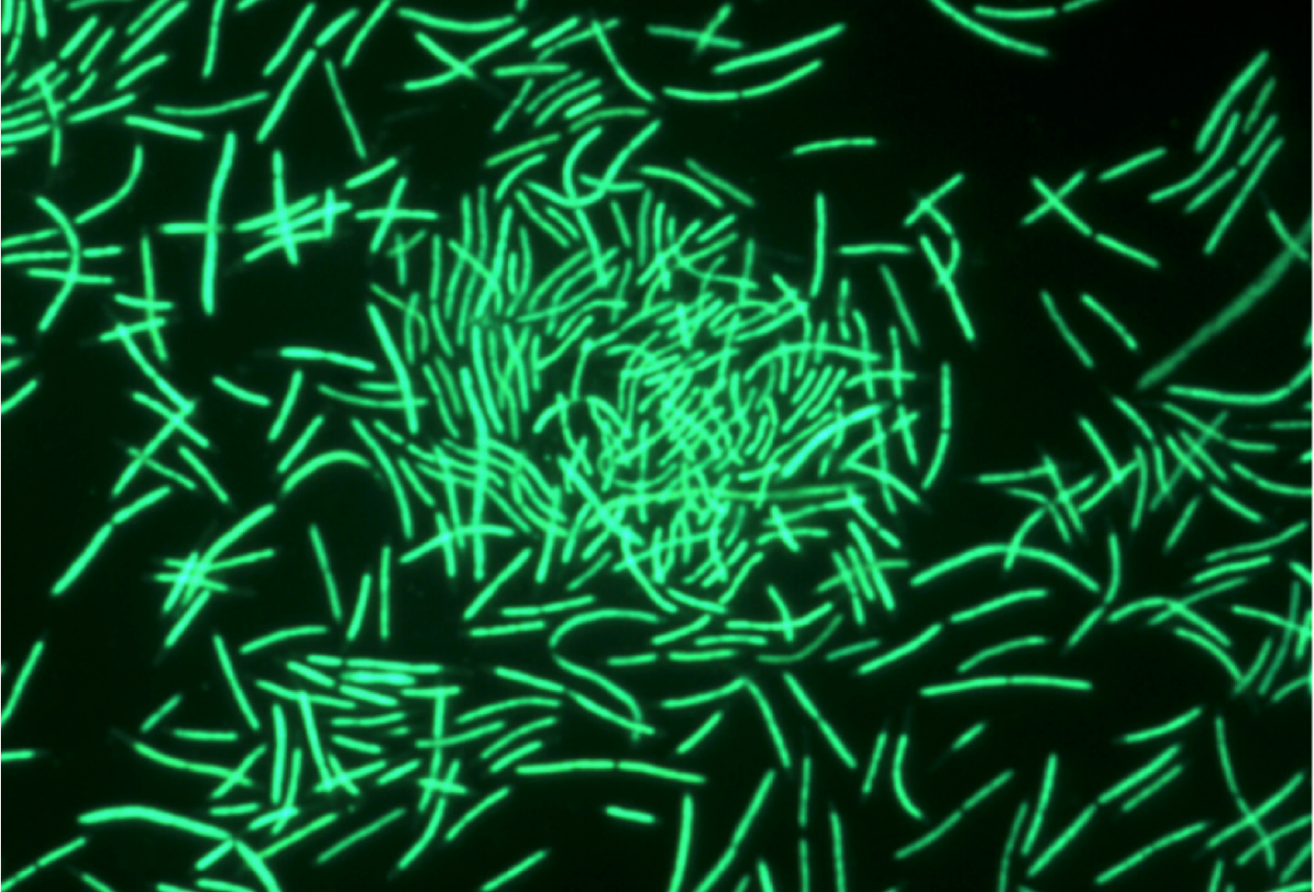
Micrograph of *Thermus aquaticus* Y51MC23 cells from aerobic cultures. Culture samples were stained with SYTO® 9 fluorescent stain in sterile water (Molecular Probes). Dark field fluorescence microscopy was performed using a Nikon Eclipse TE2000-S epifluorescence microscope at 2000× magnification and a high-pressure Hg light source.

Unlike *T*. *aquaticus* YT-1, Y51MC23 grows well under both aerobic and anaerobic conditions. Under aerobic conditions (50 ml media in 250 ml flask, 70°C, 200 rpm, silicone foam closure) the cells grew as single short rods and rosettes (Fig 6) similar to the growth observed by Brock for YT-1[1].

**Fig 7:**
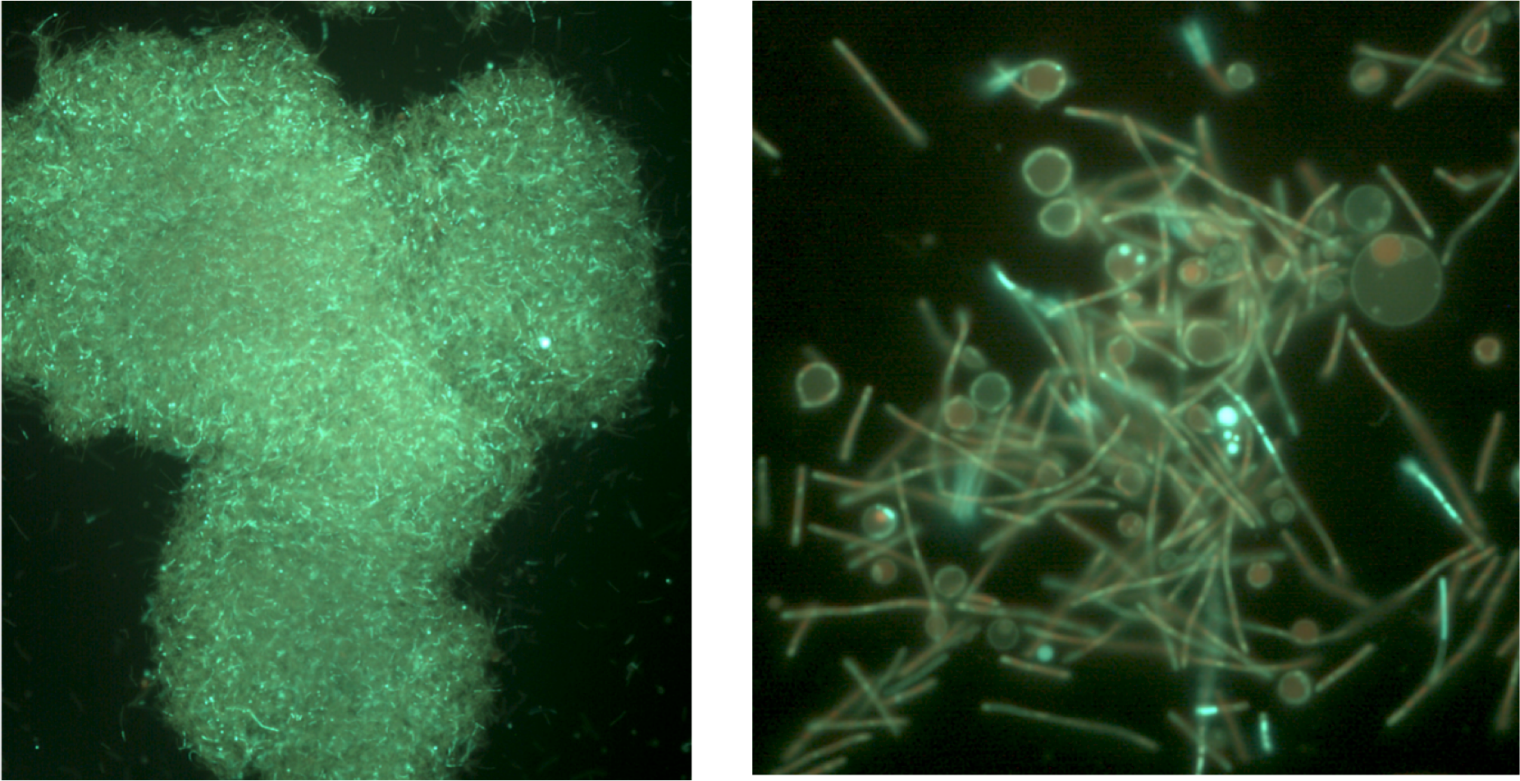
Micrograph of *Thermus aquaticus* Y51MC23 from anaerobic cultures. Culture sample was stained with SYTO® 9 fluorescent stain in sterile water (Molecular Probes). Dark field fluorescence microscopy was performed using a Nikon Eclipse TE2000-S epifluorescence microscope at 200X magnification (left) or 2000X magnification (right) and a high-pressure Hg light source (484 nm excitation and 500 nm emission filters).

**Fig 8:**
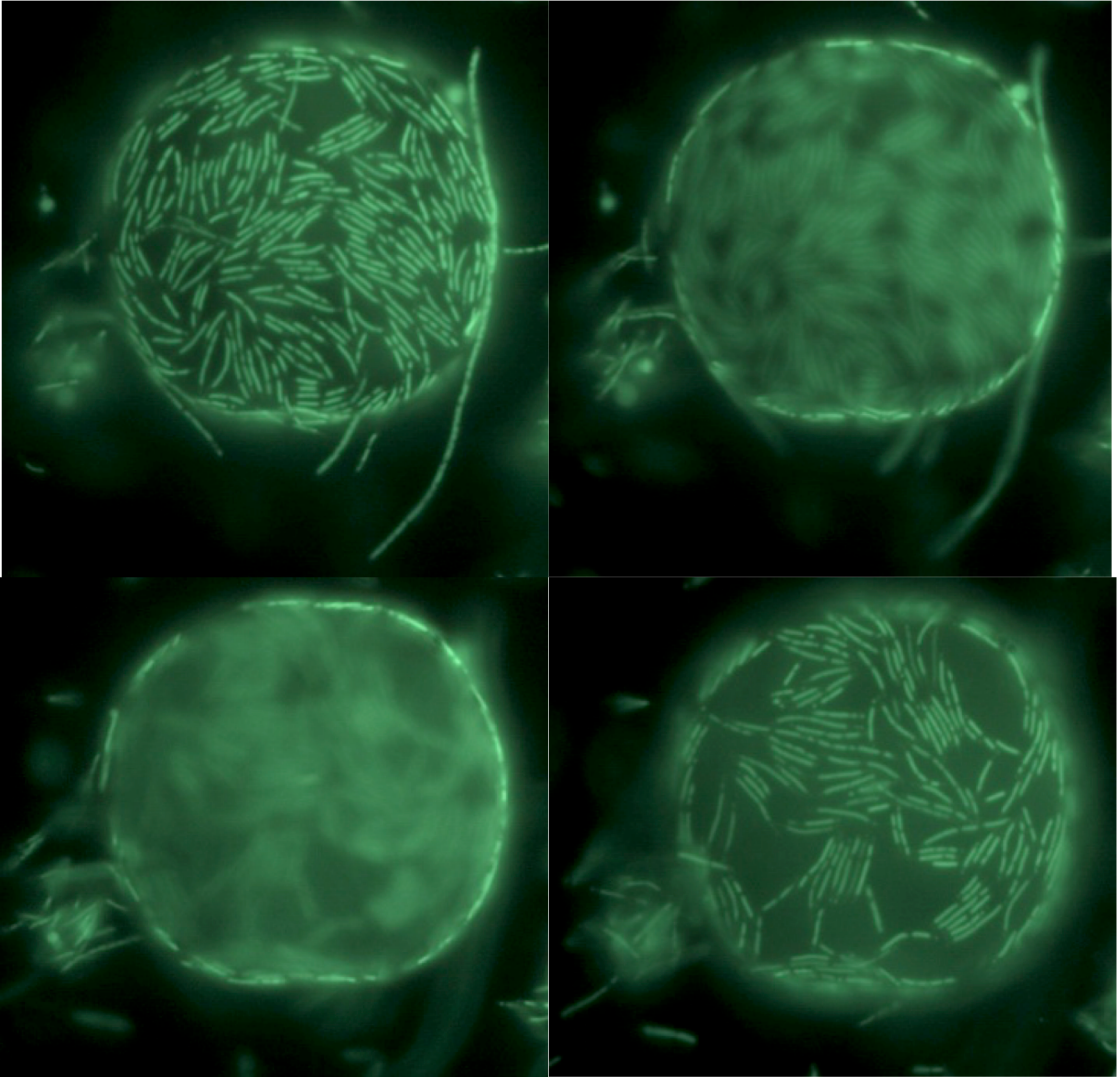
Micrograph of *Thermus aquaticus* Y51MC23 round body found in anaerobic cultures. Clockwise from left to right, views through a single large round body. Culture samples were stained with SYTO® 9 fluorescent stain in sterile water (Molecular Probes).

When grown under anaerobic conditions (50 ml media in 50 ml conical, screw cap tube or 1000 ml media in 1000 ml screw cap bottle, 70°C no agitation), the Y51MC23 grows in yellow flocculent clumps on the bottom of the container that are visible to the naked eye (Fig 7). Fluorescence microscopy of these clumps shows Y51MC23 produces a mixture of unique cell structures in high concentrations (Fig 8). The microscopic examination shows the presence of rods, but also large and small spherical bodies. Some of the spherical bodies appear strongly fluorescent, while others have a fluorescent outline and dark center. The spheres appear to have a random distribution of sizes within the layers, and some spheres appear to have smaller spheres within their walls. In addition to the numerous small spherical bodies, rod-shaped cells overlaying a tile-like layer of spherical cell bodies can be found (Fig 7), similar to but much larger than the rotund bodies described by Brock [12].

**Fig 9:**
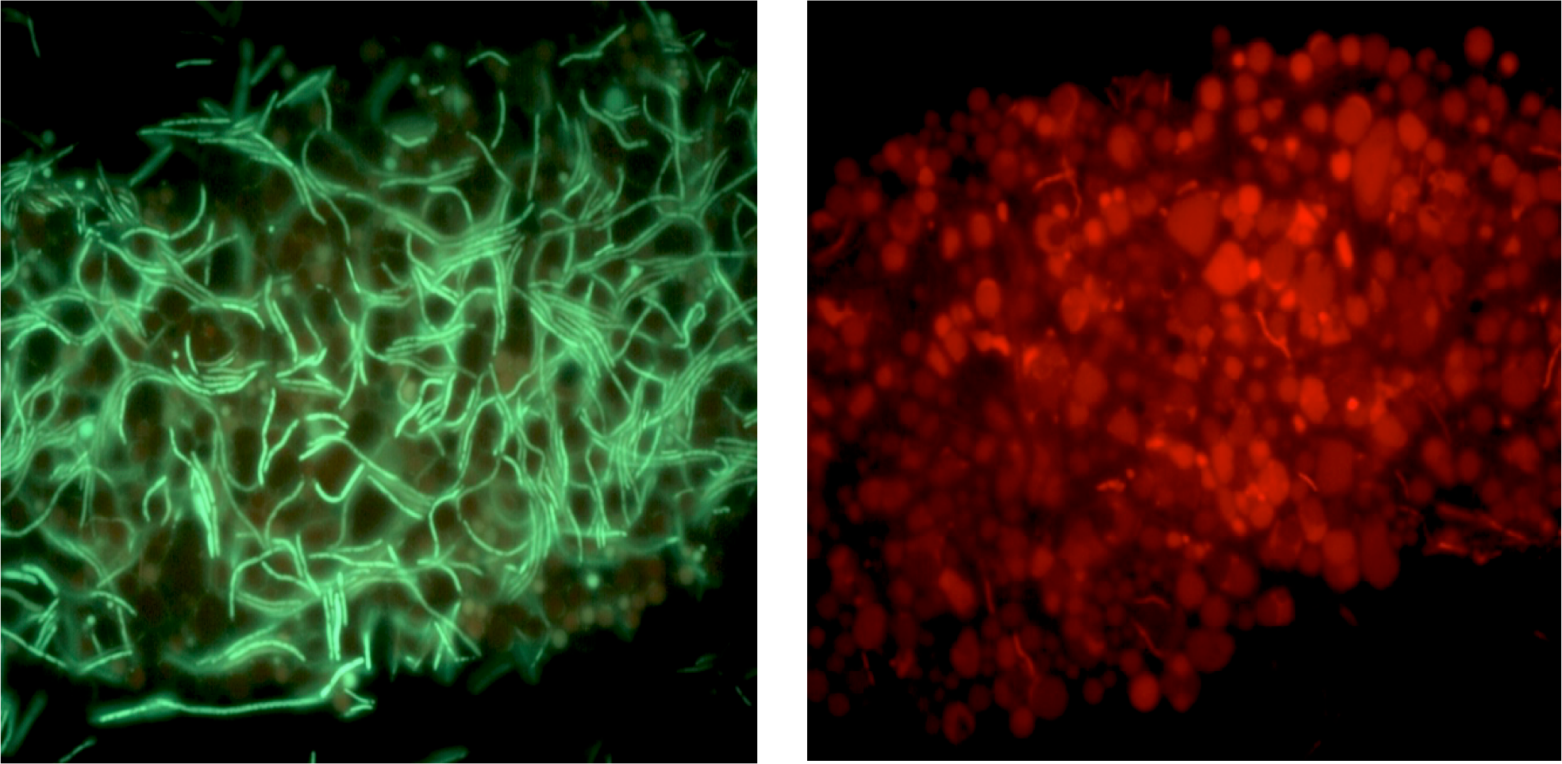
Micrograph of *Thermus aquaticus* Y51MC23 layered structure in anaerobic culture. Clumps of cells were re-suspended in sterile water and stained with SYTO® 9 (green fluroescence) using 484 nm excitation and 500 nm emission filters (left panel) or propidium iodide (red fluorescence) using 536 nm excitation and 617 nm emission filters (right panel). Dark field fluorescence microscopy was performed using a Nikon Eclipse TE2000-S epifluorescence microscope at 2000X magnification and a high-pressure Hg light source.

Clumps of cells were treated with Live-Dead® stain to improve visualization of the spherical bodies which often appear significantly less fluorescent than rod-shaped cells when stained with SYTO® 9. The multilayer structure is clearly seen as a layer of green rods on top of a matrix of red-staining spheres (Fig 9). This suggests that the multiple types of spheres seen in the micrographs may serve multiple discrete functions, including a nutrient reservoir (as in *Meiothermus ruber* [13, 14]), a biofilm-like structure to anchor cells to each other and solid surfaces, and as a source of persister cells or primitive spores [85, 86].

**Fig 10:**
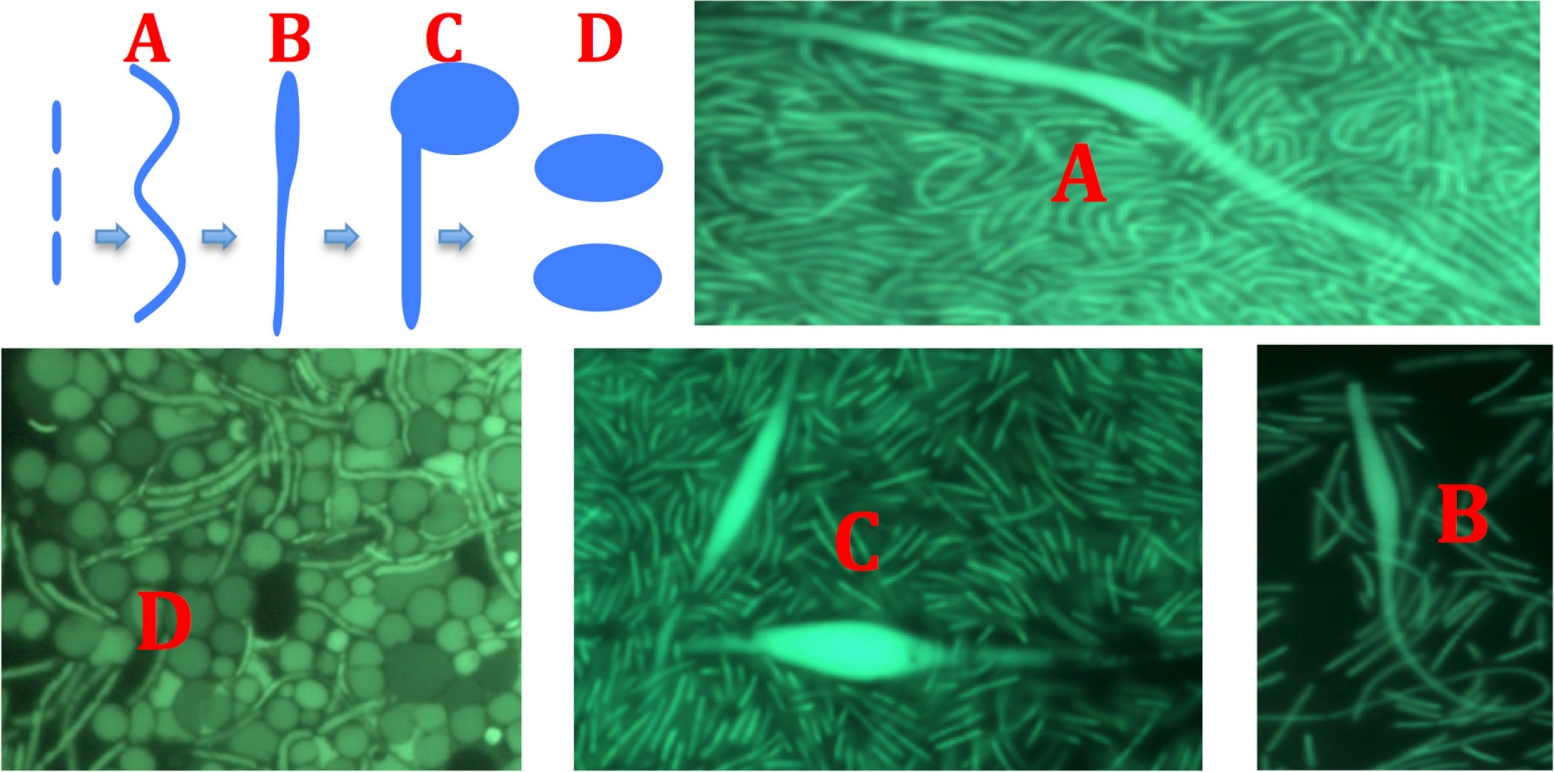
Formation of highly fluorescent spheres in Y51MC23 culture. Clockwise from top left. 1. Schematic of proposed mechanism of sphere formation. 2. Elongated cell (A) and swollen regions (B, C). 3. Highly fluorescent spheres (D). Culture samples were stained with SYTO® 9 fluorescent stain in sterile water (Molecular Probes).

Microscopy suggests that some of the highly fluorescent spherical bodies are formed by asymmetrical division of cells (Fig 10). In one scenario, an elongated cell forms in the culture (A). The elongated cell develops a swollen section, either terminal or subterminal (B, C). The swollen end separates as an irregular shaped-object (D) and appears to gradually change shape to spherical. The final shapes are spherical, opaque fluorescent spheres of various sizes.

**Fig 11:**
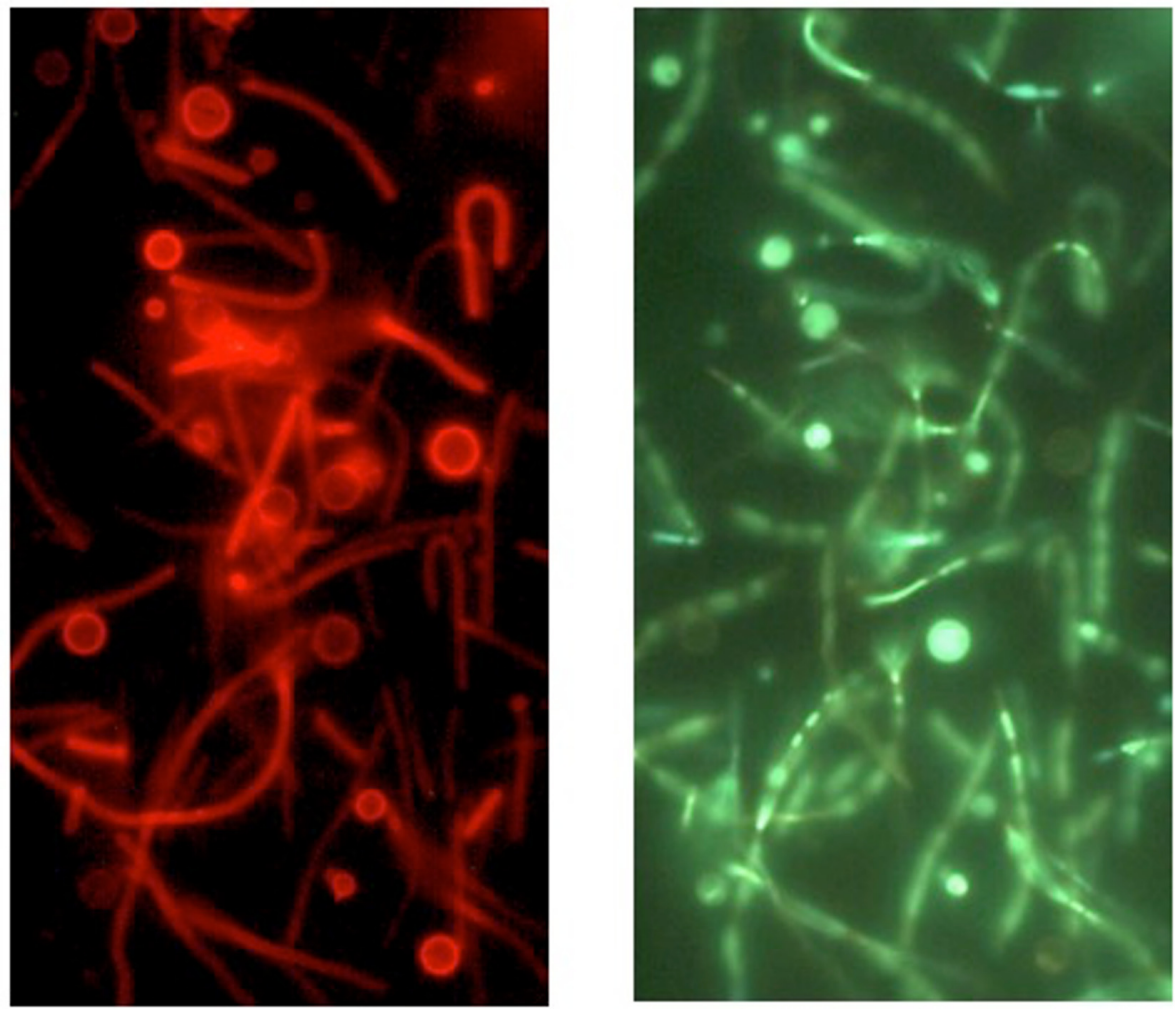
Peptidoglycan staining of Y51MC23 culture. Clumps of cells were re-suspended in sterile water and stained with SYTOX Green (green fluroescence) using 484 nm excitation and 500 nm emission filters (right panel) or Texas Red-X dye–labeled WGA (red fluorescence) using 536 nm excitation and 617 nm emission filters (left panel). Dark field fluorescence microscopy was performed using a Nikon Eclipse TE2000-S epifluorescence microscope at 2000X magnification and a high-pressure Hg light source.

Previous work using electron microscopy and visible light microscopy led to the theory that *Thermus* and *Meiothermus* spheres were formed by dissolution of the cell wall and retention of the outer envelope [12, 15]. The large spheres (rotund bodies) were formed by condensation of multiple outer envelopes. The presence of strongly-staining rings around the spheres seen in fluorescent micrographs (Fig 6) suggested that at least some of the spheres retained a thick outer structure, possibly the peptidoglycan wall. Cultures were stained with ViaGram Red^+^ Bacterial Gram Stain and Viability Kit, and examined for red fluorescence from binding of Texas Red-X dye–labeled wheat germ agglutinin (WGA) to exposed peptidoglycan. *T*. *aquaticus* vegetative cells, being Gram negative, would be expected to not bind the Texas Red-X dye–labeled WGA. The results (Fig 11) show Texas Red-X dye–labeled WGA binds strongly to the outside of many of the spheres. This indicates that spheres are formed by the loss of the outer membrane and cell envelope and remodeling of the peptidoglycan to the new shape. The presence of an intact, possibly thicker, peptidoglycan wall may also explain the stability of these spheres to osmotic and other stresses.

## Conclusions

*Thermus aquaticus*, the first thermophilic bacterium shown to grow at temperatures well over 55°C, started a revolution in thermal biology with its initial description in 1969 [1]. The ubiquitous presence of *Thermus* species [2] suggests the genus has remarkable survival adaptations not present in other organisms, resulting in extensive research to understand its role as a model organism for life at high temperatures [87].

We report here the isolation and complete genome sequence of *T*. *aquaticus* Y51MC23, a new strain isolated from the outflow of Bath Hot Spring in Yellowstone National Park. This genome was closed and finished utilizing two NGS libraries, a 350 bp fragment library, and an 8 kb mate pair library, which produced a single chromosome scaffold and four plasmids. The previous draft version could not distinguish the five separate elements and the ends of the numerous contigs often resulted in misassembled genes. A closed and finished genome simplifies comparative genomics and accurate gene annotation.

The genomes of *Thermus* species are significantly different from those of typical bacteria. *Thermus thermophilus* has been reported to possess multiple copies of the chromosome [88] as well as a 230 kb “megaplasmid” [17]. The role of the megaplasmids is believed to act as a storage site for genes related to thermophily[16]. The closed and finished genome sequence of *Thermus aquaticus* Y51MC23 shows similarities to other sequenced *Thermus* species in both the number and function of genes present in the genomes as well as short-range genomic organization. *T*. *aquaticus* Y51MC23 possesses four distinct plasmids, but no detectable megaplasmids. The generation of a finished, closed genome allowed the determination that many of the functions present on the *T*. *thermophilus* megaplasmids, such as cobalamin and phytoene biosynthetic clusters are present and located on the Y51MC23 chromosome. The organization of these clusters suggests they have arisen from stable integration of a megaplasmid into the Y51MC23 chromosome.

*T*. *aquaticus* Y51MC23 possesses significant metabolic differences from the Brock YT-1 strain, and most strains of *Thermus* in being capable of both aerobic and true fermentative growth. Most strains of *Thermus* are obligate aerobes that can also utilize nitrate as an alternative terminal electron acceptor [87, 89, 90]. The ability to utilize nitrate as an electron acceptor is conferred by a DNA fragment, dubbed the “nitrate respiratory conjugative element”, which encodes nitrate reductase and various proteins required for its activity [91]. *Thermus aquaticus* Y51MC23 possesses no orthologs of these nitrate reductase genes. Metabolic analysis suggests that Y51MC23 primarily scavenges protein from the environment, based on the high number of secreted and intracellular proteases and peptidases as well as transporter systems for amino acids and peptides. Under aerobic conditions, amino acids are most likely deaminated and the carbon backbone is oxidized using the citric acid cycle. It is unclear how ATP is generated and redox balance is maintained under anaerobic conditions.

The Y51MC23 genome shows the presence of two recognizable prophage inserts and an additional two putative prophage inserts as well as numerous CRISPR loci. The high homology and synteny between Y51MC23 prophage 2 and that of *Thermus sp*. 2.9 is ecologically interesting, given the 8,800 km separation of the two hot springs.

The anaerobic lifestyle of Y51MC23 is complex, with multiple morphologies present in cultures. The use of fluorescence microscopy reveals new details about these unusual morphological features not seen before, including the presence of multiple types of large and small spheres, often forming a confluent layer of spheres. Previous work showing rotund bodies was performed using electron microscopy, which may have destroyed many of the structural details demonstrated here using conventional light microscopy. Use of multiple dyes with different molecular specificities allowed multiple features on the same cells to be visualized for the first time. Many of the spheres seen in the micrographs appear to be formed not from cell envelope or outer membrane components [12, 15], but from a remodeled peptidoglycan cell wall. These morphological forms may serve multiple functions in the survival of the organism. Some of the spherical bodies may be a food storage system as suggested for *M*. *ruber* [13, 14]. The interlocking layer of spheres may represent an extremely resilient biofilm backbone, capable of anchoring cells to surfaces and resisting high temperatures and high liquid flow rates. Finally, some of the spheres may serve as a protective coating for genomic DNA, analogous to spores formed in Gram-positive organisms.

Numerous questions remain to be answered about Y51MC23. The source of ATP generation from amino acid fermentation remains to be discovered. How the individual cellular morphologies are generated and what molecular controls are involved in selecting morphologies remains to be elucidated. The function of the unusual high MW hypothetical proteins that are conserved in the *Deinococcus*-*Thermus* phylogenetic group also remains as a challenge to better understanding the ability to these organisms to grow and survive in high temperature, nutrient-poor, fast-flowing environments.

Thermophilic enzymes such as Taq DNA polymerase and others have proven useful for numerous industrial applications [92]. The presence of an annotated reverse transcriptase located in prophage 2 of *T*. *aquaticus* Y51MC23 (gene 1131) is of potential utility for molecular biology applications, but the protein is completely insoluble during preliminary expression experiments in our hands (data not shown). The presence of an annotated bifunctional primase/polymerase (gene 2152) also has potential utility for whole genome amplification depending on the appropriate biochemical characteristics.

## Supporting Information

S1 Fig PCR verification of contig order and orientation. (DOCX)

S1 Table PCR primers used to cross areas of uncertainty. (DOCX)

S2 Table Comparison of the finished chromosome and its four attendant plasmids and the draft assembly. (PDF)
