# Supplementary material for: Complete Genome Sequence of Thermus aquaticus Y51MC23
Source: PLoS One. 2015 Oct 14;10(10):e0138674. doi: 10.1371/journal.pone.0138674 (PMC4605624; doi:10.1371/journal.pone.0138674)
Supplement: S1 Fig — (DOCX) [file pone.0138674.s001.docx]

**Supplementary Figure 1.**


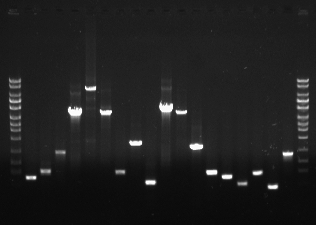


10,000

6,000

4,000

3,000

2,000

1,000

700

500

300

10,000

6,000

4,000

3,000

2,000

1,000

700

500

300

**Pair**

**1**

**2**

**3**

**4**

**5**

**6**

**7**

**8**

**9**

**10**

**11**

**12**

**13**

**14**

**15**

**16**

**17**

**18**

**Supplementary Figure 1. PCR verification of contig order and orientation in assembly of *T. aquaticus* genome.** A 1.7% agarose gel stained with ethidium bromide was electrophoresed at 130 V for 45 min. The DNA size ladder is Lucigen’s GelReady 1 kb. Lane labels in figure correspond directly to the Pair # column in Table 1, such that Pair 1 refers to the pair of Primers 2 and 3, Pair 2 refers to the pair of Primers 4 and 5, and so on.
