# Supplementary material for: Complete Genome Sequence of Thermus aquaticus Y51MC23
Source: PLoS One. 2015 Oct 14;10(10):e0138674. doi: 10.1371/journal.pone.0138674 (PMC4605624; doi:10.1371/journal.pone.0138674)
Supplement: S1 Table — (DOCX) [file pone.0138674.s002.docx]

**Supplementary Table 1. PCR primers used to verify *Thermus aquaticus* genome assembly.**
