# Supplementary material for: Complete Genome Sequence of Thermus aquaticus Y51MC23
Source: PLoS One. 2015 Oct 14;10(10):e0138674. doi: 10.1371/journal.pone.0138674 (PMC4605624; doi:10.1371/journal.pone.0138674)
Supplement: S2 Table — (PDF) [file pone.0138674.s003.pdf]

Gene	Length	Product	TaqDRAFT gene	TaqDRAFT Contig
1	442	Chromosomal replication initiator protein DnaA	4631	14
2	372	DNA polymerase III beta subunit (EC 2.7.7.7)	4630	14
3	423	Enolase (EC 4.2.1.11)	4629	14
4	475	Pyruvate kinase (EC 2.7.1.40)	4628	14
5	143	Cytochrome c-552 precursor	4627	14
6	261	Radical SAM domain heme biosynthesis protein	4626	14
7	205	Metal dependent hydrolase (EC 3.-.-.-)	4625	14
8	616	1-deoxy-D-xylulose 5-phosphate synthase (EC 2.2.1.7)		
9	210	FIG01181671: hypothetical protein	4622	14
10	95	hypothetical protein	4621	14
11	221	Phage shock protein A homolog	4620	14
12	246	FIG01181333: hypothetical protein	4619	14
13	213	FIG01181350: hypothetical protein	4618	14
14	304	Cysteine synthase (EC 2.5.1.47)	4617	14
15	156	FKBP-type peptidyl-prolyl cis-trans isomerase SlyD (EC 5.2.1.8)	4616	14
16	69	Cold shock protein CspA	4615	14
17	250	5-nucleotidase SurE (EC 3.1.3.5)	4614	14
18	227	Tll2177 protein	4613	14
19	543	Mobile element protein	4612	14
20	482	FIG01181649: hypothetical protein	4611	14
21	38	hypothetical protein	4610	14
22	195	Putative tetratricopeptide repeat family protein	4609	14
23	199	Protein of unknown function UPF0126	4608	14
24	153	2-C-methyl-D-erythritol 2,4-cyclodiphosphate synthase (EC 4.6.1.12)	4607	14
25	184	Hydrolase (HAD superfamily)	4606	14
26	107	FIG01181552: hypothetical protein	4605	14
27	187	5-FCL-like protein, but predicted not to be 5-formyltetrahydrofolate cyclo-ligase (5-FCL)	4604	14
28	80	FIG01181245: hypothetical protein	4603	14
29	1646	DNA polymerase III alpha subunit (EC 2.7.7.7) @ intein-containing	4602	14
30	544	Heat shock protein 60 family chaperone GroEL	4601	14
31	102	Heat shock protein 60 family co-chaperone GroES	4600	14
32	263	RNA methyltransferase, TrmH family	4599	14
33	743	6-carboxyhexanoate--CoA ligase	4598	14
34	57	FIG01181111: hypothetical protein	4597	14
35	135	hypothetical conserved protein	4596	14
36	430	Maltose/maltodextrin ABC transporter, substrate binding periplasmic protein MalE	4594	14
37	292	Maltose/maltodextrin ABC transporter, permease protein MalF	4593	14
38	281	Maltose/maltodextrin ABC transporter, permease protein MalG	4592	14
39	66	hypothetical protein		
40	59	hypothetical protein	4591	14
41	525	Phosphoglucomutase (EC 5.4.2.2)	4590	14
42	604	Peptidyl-prolyl cis-trans isomerse, PpiC family	4589	14
43	378	fosmidomycin resistance protein	4588	14
44	269	Universal stress protein UspA and related nucleotide-binding proteins	4587	14
45	144	Inosine-5'-monophosphate dehydrogenase (EC 1.1.1.205)	4586	14
46	116	FIG00788328: hypothetical protein	4585	14
47	210	phosphoglycerate mutase family protein	4648	6
48	334	Phosphoribosylformylglycinamidine cyclo-ligase (EC 6.3.3.1)	4649	6
49	470	Aspartyl-tRNA(Asn) amidotransferase subunit B (EC 6.3.5.6) @ Glutamyl-tRNA(Gln) amidotransferase	4650	6
50	363	Twitching motility protein PilT	4651	6
51	890	Phage adsorption protein / Type IV fimbrial assembly, ATPase PilB	4652	6
52	161	FIG001553: Hydrolase, HAD subfamily IIIA	4653	6
53	245	COG1496: Uncharacterized conserved protein	4654	6
54	135	FIG01181098: hypothetical protein	4655	6
55	158	6,7-dimethyl-8-ribityllumazine synthase (EC 2.5.1.78)	4656	6
56	128	CrcB protein	4657	6
57	111	FIG01181425: hypothetical protein	4658	6
58	203	sulfite dehydrogenase	4659	6
59	90	FIG01181249: hypothetical protein	4660	6
60	263	Histidinol-phosphatase (EC 3.1.3.15)	4661	6
61	199	FIG01180928: hypothetical protein	4662	6
62	197	Nicotinamidase (EC 3.5.1.19)	4663	6
63	305	5,10-methylenetetrahydrofolate reductase (EC 1.5.1.20)	4664	6
64	77	Metallo-beta-lactamase family protein, RNA-specific	4665	6
65	407	Translation elongation factor Tu	4666	6
66	55	LSU ribosomal protein L33p @ LSU ribosomal protein L33p, zinc-dependent	4667	6
67	61	Preprotein translocase subunit SecE (TC 3.A.5.1.1)	4668	6

68	185	Transcription antitermination protein NusG	4669	6
69	148	LSU ribosomal protein L11p (L12e)	4670	6
70	230	LSU ribosomal protein L1p (L10Ae)	4671	6
71	174	LSU ribosomal protein L10p (P0)	4672	6
72	126	LSU ribosomal protein L7/L12 (P1/P2)	4673	6
73	192	UbiX family decarboxylase associated with menaquinone via futalosine	4674	6
74	373	Butyryl-CoA dehydrogenase (EC 1.3.99.2)	4675	6
75	264	Undecaprenyl diphosphate synthase (EC 2.5.1.31)	4676	6
76	377	Homocitrate synthase (EC 2.3.3.14)	4677	6
77	69	FIG01180971: hypothetical protein	4678	6
78	76	FIG01181435: hypothetical protein	4679	6
79	418	Homoaconitase large subunit (EC 4.2.1.36)	4680	6
80	164	Homoaconitase small subunit (EC 4.2.1.36)	4681	6
81	107	OrfE	4682	6
82	55	Lysine biosynthesis protein LysW	4683	6
83	282	Lysine biosynthesis protein LysX	4684	6
84	345	N-acetyl-gamma-aminoadipyl-phosphate reductase (EC 1.2.1.-)	4685	6
85	270	Acetylaminoadipate kinase (EC 2.7.2.-)	4686	6
86	532	Glycogen branching enzyme, GH-57-type, archaeal (EC 2.4.1.18)	4687	6
87	292	Glutamine amidotransferase chain of NAD synthetase	4688	6
88	282	NAD synthetase (EC 6.3.1.5)	4689	6
89	141	Succinyl-CoA synthetase, alpha subunit-related enzymes	4690	6
90	334	A/G-specific adenine glycosylase (EC 3.2.2.-)	4691	6
91	133	Glucose-inhibited division protein A	4692	6
92	91	CRISPR-associated protein Cas2	4693	6
93	317	CRISPR-associated protein Cas1	4694	6
94	402	CRISPR-associated protein Cas02710	4695	6
95	205	Invasion associated protein p60	4696	6
96	307	oxygen-insesitive NAD(P)H nitroreductase/dihydropteridine reductase	4697	6
97	168	FIG01181105: hypothetical protein	4698	6
98	356	Leader peptidase (Prepilin peptidase) (EC 3.4.23.43) / N-methyltransferase (EC 2.1.1.-)	4699	6
99	512	Thermostable carboxypeptidase 1 (EC 3.4.17.19)	4700	6
100	108	alternate gene name: jojD	4701	6
101	344	Probable endoglucanase (EC 3.2.1.4)	4702	6
102	127	Methylglyoxal synthase (EC 4.2.3.3)	4703	6
103	495	Mg(2+) Chelatase family protein / ComM-related protein	4704	6
104	377	Major facilitator family transporter	4705	6
105	271	Permease	4706	6
106	295	Permease of the drug/metabolite transporter (DMT) superfamily	4707	6
107	376	NAD(P) transhydrogenase alpha subunit (EC 1.6.1.2)	4708	6
108	100	NAD(P) transhydrogenase alpha subunit (EC 1.6.1.2)	4709	6
109	451	NAD(P) transhydrogenase subunit beta (EC 1.6.1.2)	4710	6
110	102	SSU ribosomal protein S6p	4711	6
111	268	Single-stranded DNA-binding protein / Single-stranded DNA-binding protein	4712	6
112	90	SSU ribosomal protein S18p @ SSU ribosomal protein S18p, zinc-independent	4713	6
113	149	LSU ribosomal protein L9	4714	6
114	471	Lactate 2-monooxygenase (EC 1.13.12.4)	4715	6
115	276	Corticosteroid 11-beta-dehydrogenase	4716	6
116	220	Octanoate-[acyl-carrier-protein]-protein-N-octanoyltransferase	4717	6
117	324	Lipoate synthase	4718	6
118	116	hypothetical protein	4719	6
119	290	3-hydroxyisobutyrate dehydrogenase	4720	6
120	465	Dihydrolipoamide dehydrogenase of branched-chain alpha-keto acid dehydrogenase (EC 1.8.1.4)	4721	6
121	455	Dihydrolipoamide acyltransferase component of branched-chain alpha-keto acid dehydrogenase com	4722	6
122	66	hypothetical protein	4723	6
123	325	Branched-chain alpha-keto acid dehydrogenase, E1 component, beta subunit (EC 1.2.4.4)	4724	6
124	368	Branched-chain alpha-keto acid dehydrogenase, E1 component, alpha subunit (EC 1.2.4.4)	4725	6
125	136	LSU m3Psi1915 methyltransferase RlmH	4726	6
126	132	conserved hypothetical protein	4727	6
127	142	FIG01181319: hypothetical protein	4728	6
128	295	Biotin--protein ligase (EC 6.3.4.9, EC 6.3.4.10, EC 6.3.4.11, EC 6.3.4.15) / Biotin operon repressor	4729	6
129	349	Glucose-1-phosphate thymidylyltransferase (EC 2.7.7.24)	4730	6
130	268	Methyltransferase	4731	6
131	127	UPF0047 protein Bsu YugU	4732	6
132	727	GTP pyrophosphokinase (EC 2.7.6.5), (p)ppGpp synthetase II / Guanosine-3',5'-bis(diphosphate) 3'-py	4733	6
133	747	plectin 1 isoform 8	4734	6
134	370	Potassium efflux system KefA protein / Small-conductance mechanosensitive channel	4735	6
135	247	Hemoprotein HemQ, essential component of heme biosynthetic pathway in Gram-positive bacteria	4736	6

136	165	Chlorite dismutase precursor	4737	6
137	192	Holliday junction DNA helicase RuvA	4738	6
138	262	Enoyl-CoA hydratase (EC 4.2.1.17)	4739	6
139	895	2-oxoglutarate dehydrogenase E1 component (EC 1.2.4.2)	4740	6
140	395	Dihydrolipoamide succinyltransferase component (E2) of 2-oxoglutarate dehydrogenase complex (EC	4741	6
141	456	Dihydrolipoamide dehydrogenase of 2-oxoglutarate dehydrogenase (EC 1.8.1.4)	4742	6
142	550	Serine protease, subtilase family	4743	6
143	676	FIG01181470: hypothetical protein	4744	6
144	401	Argininosuccinate synthase (EC 6.3.4.5)	4745	6
145	463	Argininosuccinate lyase (EC 4.3.2.1)	4746	6
146	181	N-acetylglutamate synthase (EC 2.3.1.1)	4747	6
147	85	FIG01181457: hypothetical protein	4748	6
148	93	hypothetical protein	4749	6
149	389	Carbamoyl-phosphate synthase small chain (EC 6.3.5.5)	4750	6
150	544	Formate--tetrahydrofolate ligase (EC 6.3.4.3)	4752	6
151	311	Threonine dehydratase, catabolic (EC 4.3.1.19)	4753	6
152	76	hypothetical protein	4754	6
153	378	**Integrase**	4755	6
154	123	hypothetical protein	4756	6
155	111	Signal peptidase I (EC 3.4.21.89)	4757	6
156	78	hypothetical protein	4758	6
157	86	hypothetical protein	4759	6
158	126	Putative DnaJ chaperone	4760	6
159	192	**Hypothetical**	4761	6
160	209	**DNA primase**	4762	6
161	313	**Hypothetical**	4763	6
162	543	**UvrD/REP Helicase**	4764	6
163	226	**Hypothetical**	4765	6
164	**371**	**DNA polymerase III beta clamp processivity factor**	**4767**	6
165	**56**	**Hypothetical**	**4768**	6
166	**119**	**Holliday junction resolvase (endodeoxyribonuclease RusA)**	**4769**	6
167	**107**	**Transcriptional regulatory, Fis family**	**4770**	6
168	**427**	**Phage terminase packaging enzyme, large subunit**	**4771**	6
169	67	**Hypothetical**	4772	6
170	494	**MU like phage gp29 protein**	4773	6
171	481	**Putative phage head morphogenesis protein**	4774	6
172	104	**Hypothetical**	4775	6
173	433	**Mu-like phage gp32 protein**	4776	6
174	**286**	**Putative major capsid protein**	4777	6
175	70	**Putative DNA binding protein**	4778	6
176	95	**Mu-like phage gp36 protein**	4779	6
177	174	**Putative phage tail completion**	4780	6
178	145	**Hypothetical**	4781	6
179	69	**Hypothetical**	4782	6
180	472	**Phage tail sheath protein**	4783	6
181	146	**Hypothetical**	4784	6
182	108	**Hypothetical**	4785	6
183	40	**Hypothetical**	4786	6
184	822	**Phage tape measure protein**	4787	6
185	182	**Phage tail protein**	4788	6
186	224	**Hypothetical**	4789	6
187	166	**Hypothetical**	4790	6
188	126	**Baseplate assembly protein with lysozyme**	4791	6
189	367	**Baseplate protein**	4792	6
190	245	**Phage tail protein**	4793	6
191	71	**Hypothetical**	4794	6
192	104	**Hypothetical**	4795	6
193	288	**Hypothetical**	4796	6
194	121	**Hypothetical**	4797	6
195	359	Conserved hypothetical protein	4798	6
196	**101**	**Hypothetical**	4799	1
197	53	**Hypothetical**	4800	1
198	63	**Hypothetical**	4801	1
199	102	**Hypothetical**	4802	1
200	219	**Peptidase M23**	4803	1
201	89	LSU ribosomal protein L21p	4804	1
202	86	LSU ribosomal protein L27p	4805	1
203	418	GTP-binding protein Obg	4806	1

204	187	Nicotinate-nucleotide adenylyltransferase (EC 2.7.7.18)	4807	1
205	186	Hydrolase (HAD superfamily), YqeK	4808	1
206	366	Cell envelope-associated transcriptional attenuator LytR-CpsA-Psr, subfamily M (as in PMID1909955€	4809	1
207	114	Ribosomal silencing factor RsfA (former Iojap)	4810	1
208	94	FIG01181869: hypothetical protein	4811	1
209	277	Pantoate--beta-alanine ligase (EC 6.3.2.1)	4812	1
210	369	Twitching motility protein PilT	4813	1
211	306	Fructose-bisphosphate aldolase class II (EC 4.1.2.13)	4814	1
212	173	COG1355, Predicted dioxygenase	4815	1
213	424	Pyrimidine-nucleoside phosphorylase (EC 2.4.2.2)	4816	1
214	300	cell wall endopeptidase, family M23/M37	4817	1
215	121	Integral membrane protein CcmA involved in cell shape determination	4818	1
216	286	Acetyl-coenzyme A carboxyl transferase beta chain (EC 6.4.1.2)	4819	1
217	317	Acetyl-coenzyme A carboxyl transferase alpha chain (EC 6.4.1.2)	4820	1
218	535	S-layer protein	4821	1
219	206	FIG038982: hypothetical protein	4822	1
220	226	FIG01181265: hypothetical protein	4823	1
221	179	hypothetical conserved protein	4824	1
222	77	Ferredoxin	4825	1
223	167	Acetyltransferase	4826	1
224	74	Cold shock protein CspG	4827	1
225	348	Chaperone protein DnaJ	4828	1
226	354	Serine--pyruvate transaminase (EC 2.6.1.51)	4829	1
227	259	Undecaprenyl-diphosphatase (EC 3.6.1.27)	4830	1
228	217	2-C-methyl-D-erythritol 4-phosphate cytidylyltransferase (EC 2.7.7.60)	4831	1
229	273	4-diphosphocytidyl-2-C-methyl-D-erythritol kinase (EC 2.7.1.148)	4832	1
230	190	TenA2, thiaminase II (EC 3.5.99.2) homolog involved in salvage of thiamin pyrimidine moiety	4833	1
231	165	CarD-like transcriptional regulator	4834	1
232	187	Transcriptional regulator, TetR family	4835	1
233	238	Cyanophycinase (EC 3.4.15.6)	4836	1
234	167	FIG01181396: hypothetical protein	4837	1
235	162	Thio-disulfide isomerase/thioredoxin	4838	1
236	146	TsaE protein, required for threonylcarbamoyladenosine t(6)A37 formation in tRNA	4839	1
237	197	Molybdopterin oxidoreductase subunit, predicted; chaperone protein HtpG	4840	1
238	878	Molybdopterin oxidoreductase, iron-sulfur binding subunit (EC 1.2.7.-)	4842	1
239	450	Molybdopterin oxidoreductase (EC 1.2.7.-)	4843	1
240	173	ABC-type Fe3+ transport system protein; Molybdenum transport protein, putative	4844	1
241	170	ABC-type Fe3+ transport system protein; Molybdenum transport protein, putative	4845	1
242	334	Putative uncharacterized protein TTHA1760	4846	1
243	537	FIG01181677: hypothetical protein	4848	1
244	359	N-acetyl-lysine deacetylase (EC 3.5.1.-)	4849	1
245	397	N-acetyl-lysine aminotransferase (EC 2.6.1.-); Acetylornithine aminotransferase (EC 2.6.1.11)	4850	1
246	380	Glycosyl transferase group 1	4851	1
247	328	Mannosyltransferase WbkA	4852	1
248	337	putative glycosyltransferase	4853	1
249	337	Mannose-1-phosphate guanylyltransferase (GDP) (EC 2.7.7.22)	4854	1
250	160	2-amino-4-hydroxy-6-hydroxymethyldihydropteridine pyrophosphokinase (EC 2.7.6.3)	4855	1
251	126	Polyribonucleotide nucleotidyltransferase (EC 2.7.7.8)	4856	1
252	111	Thioredoxin	4857	1
253	327	Phosphate:acyl-ACP acyltransferase PlsX	4858	1
254	140	Archease	4859	1
255	224	LmbE-related protein	4860	1
256	110	hypothetical protein	4861	1
257	260	Orotidine 5'-phosphate decarboxylase (EC 4.1.1.23)	4862	1
258	184	Orotate phosphoribosyltransferase (EC 2.4.2.10)	4863	1
259	333	FIG01181067: hypothetical protein	4864	1
260	322	Glycerol-3-phosphate dehydrogenase [NAD(P)+] (EC 1.1.1.94)	4865	1
261	179	FIG01180873: hypothetical protein	4866	1
262	237	Beta-ketoadipate enol-lactone hydrolase (EC 3.1.1.24)	4867	1
263	69	FIG01181293: hypothetical protein	4868	1
264	139	Putative iron-sulfur cluster assembly scaffold protein for SUF system, SufE2	4869	1
265	405	Cysteine desulfurase (EC 2.8.1.7), SufS subfamily	4870	1
266	94	hypothetical protein	4871	1
267	792	Lead, cadmium, zinc and mercury transporting ATPase (EC 3.6.3.3) (EC 3.6.3.5); Copper-translocating	4872	1
269	95	hypothetical cytosolic protein	4875	1
270	67	Copper chaperone	4876	1
271	129	Peptidase, M23/M37 family	4877	1
272	553	FIG00677838: hypothetical protein	4878	1

273	101	Ferredoxin, 2Fe-2S	4879	1
274	432	Iron-sulfur cluster assembly protein SufD	4880	1
275	469	Iron-sulfur cluster assembly protein SufB	4881	1
276	251	Iron-sulfur cluster assembly ATPase protein SufC	4882	1
277	933	FIG01181380: hypothetical protein	4883	1
278	440	Isocitrate lyase (EC 4.1.3.1)	4884	1
279	444	FIG01181220: hypothetical protein	4885	1
280	48	hypothetical protein	4886	1
281	231	Methionine ABC transporter ATP-binding protein	4887	1
282	381	ABC transporter, permease protein	4888	1
283	88	FIG01181054: hypothetical protein	4890	1
284	882	Alanyl-tRNA synthetase (EC 6.1.1.7)	4891	1
285	229	FIG01180980: hypothetical protein	4892	1
286	129	Putative Holliday junction resolvase YqgF	4893	1
287	338	FIG004453: protein YceG like	4894	1
288	280	Zinc transport protein ZntB	4895	1
289	164	FIG01181242: hypothetical protein	4896	1
290	389	hypothetical protein	4897	1
291	239	hypothetical cytosolic protein	4898	1
292	363	Muconate cycloisomerase (EC 5.5.1.1)	4899	1
293	83	hypothetical protein	4900	1
294	264	hypothetical protein	4901	1
295	73	hypothetical protein	4902	1
296	210	Alr0728 protein	4903	1
297	343	Permeases of the major facilitator superfamily	4904	1
298	258	Folate-dependent protein for Fe/S cluster synthesis/repair in oxidative stress	4905	1
299	395	Molybdopterin binding motif, CinA N-terminal domain / C-terminal domain of CinA type S	4906	1
300	198	2'-5' RNA ligase	4907	1
301	341	RecA protein	4908	1
302	576	FIG002344: Hydrolase (HAD superfamily)	4909	1
303	344	Rod shape-determining protein MreB	4910	1
304	144	3-hydroxyacyl-[acyl-carrier-protein] dehydratase, FabZ form (EC 4.2.1.59)	4911	1
305	211	FIG001583: hypothetical protein, contains S4-like RNA binding domain	4912	1
306	1120	DNA-directed RNA polymerase beta subunit (EC 2.7.7.6)	4913	1
307	1525	DNA-directed RNA polymerase beta' subunit (EC 2.7.7.6)	4914	1
308	193	Ribulose-5-phosphate 4-epimerase and related epimerases and aldolases	4915	1
309	80	FIG01180871: hypothetical protein	4916	1
310	265	Proline iminopeptidase-related protein	4917	1
311	345	Thiamin ABC transporter, substrate-binding component	4918	1
312	503	Thiamin ABC transporter, transmembrane component	4919	1
313	265	Formamidopyrimidine-DNA glycosylase (EC 3.2.2.23)	4920	1
314	102	FIG01181016: hypothetical protein	4921	1
315	125	Acyl-CoA hydrolase (EC 3.1.2.20)	4922	1
316	81	Pterin-4-alpha-carbinolamine dehydratase (EC 4.2.1.96)	4923	1
317	459	D-Lactate dehydrogenase, cytochrome c-dependent (EC 1.1.2.4)	4924	1
318	378	hypothetical protein	4925	1
319	41	hypothetical protein		1
320	191	hypothetical protein	4926	1
321	543	Mobile element protein	4927	1
322	201	hypothetical protein	4928	1
323	399	Putative permease	4929	1
324	492	Radical SAM domain heme biosynthesis protein	4930	1
325	495	hypothetical protein	4931	1
326	199	Probable macrolide-efflux transmembrane protein	4932	1
327	174	hypothetical protein	4933	1
328	317	Oxidoreductase	4934	1
329	70	hypothetical conserved protein	4935	1
330	158	FIG01181295: hypothetical protein	4936	1
331	768	Penicillin acylase (EC 3.5.1.11)	4937	1
332	697	ATP-dependent protease La (EC 3.4.21.53) Type II	4938	1
333	415	Glucose-1-phosphate adenylyltransferase (EC 2.7.7.27)	4939	1
334	229	Glucose-1-phosphate adenylyltransferase(EC:2.7.7.27)	4940	1
335	225	Two-component response regulator	4941	1
336	125	FIG01181346: hypothetical protein	4942	1
337	441	Glycogen synthase, ADP-glucose transglucosylase (EC 2.4.1.21)	4943	1
338	194	Tetratricopeptide TPR_2 repeat protein	4944	1
339	343	tRNA dihydrouridine synthase A	4945	1
340	341	4-hydroxy-3-methylbut-2-enyl diphosphate reductase (EC 1.17.1.2)	4946	1

341	197	FIG01181466: hypothetical protein	4947	1
342	64	FIG01181862: hypothetical protein	4948	1
343	331	Octaprenyl diphosphate synthase (EC 2.5.1.90) / Dimethylallyltransferase (EC 2.5.1.1)	4949	1
344	277	Permease of the drug/metabolite transporter (DMT) superfamily	4950	1
345	335	Molybdenum cofactor biosynthesis protein MoaA	4951	1
346	59	Mobile element protein	4952	1
347	325	Mobile element protein	4953	1
348	124	hypothetical protein	4954	1
349	146	hypothetical protein	4955	1
350	372	Lipoprotein releasing system transmembrane protein LolC	4956	1
351	137	SSU ribosomal protein S12p (S23e)	4957	1
352	156	SSU ribosomal protein S7p (S5e)	4958	1
353	692	Translation elongation factor G	4959	1
354	407	Translation elongation factor Tu	4960	1
355	106	SSU ribosomal protein S10p (S20e)	4961	1
356	207	LSU ribosomal protein L3p (L3e)	4962	1
357	214	LSU ribosomal protein L4p (L1e)	4963	1
358	97	LSU ribosomal protein L23p (L23Ae)	4964	1
359	277	LSU ribosomal protein L2p (L8e)	4965	1
360	94	SSU ribosomal protein S19p (S15e)	4966	1
361	114	LSU ribosomal protein L22p (L17e)	4967	1
362	240	SSU ribosomal protein S3p (S3e)	4968	1
363	142	LSU ribosomal protein L16p (L10e)	4969	1
364	73	LSU ribosomal protein L29p (L35e)	4970	1
365	106	SSU ribosomal protein S17p (S11e)	4971	1
366	123	LSU ribosomal protein L14p (L23e)	4972	1
367	111	LSU ribosomal protein L24p (L26e)	4973	1
368	183	LSU ribosomal protein L5p (L11e)	4974	1
369	62	SSU ribosomal protein S14p (S29e) @ SSU ribosomal protein S14p (S29e), zinc-dependent	4975	1
370	139	SSU ribosomal protein S8p (S15Ae)	4976	1
371	181	LSU ribosomal protein L6p (L9e)	4977	1
372	113	LSU ribosomal protein L18p (L5e)	4978	1
373	158	SSU ribosomal protein S5p (S2e)	4979	1
374	61	LSU ribosomal protein L30p (L7e)	4980	1
375	151	LSU ribosomal protein L15p (L27Ae)	4981	1
376	439	Preprotein translocase secY subunit (TC 3.A.5.1.1)	4982	1
377	185	Adenylate kinase (EC 2.7.4.3)	4983	1
378	256	Methionine aminopeptidase (EC 3.4.11.18)	4984	1
379	73	Translation initiation factor 1	4985	1
380	38	LSU ribosomal protein L36p	4986	1
381	127	SSU ribosomal protein S13p (S18e)	4987	1
382	117	SSU ribosomal protein S11p (S14e)	4988	1
383	194	SSU ribosomal protein S4p (S9e)	4989	1
384	315	DNA-directed RNA polymerase alpha subunit (EC 2.7.7.6)	4990	1
385	119	LSU ribosomal protein L17p	4991	1
386	160	FIG01181730: hypothetical protein	4992	1
387	278	Zinc transport protein ZntB	4993	1
388	226	Pullulanase type II, GH13 family	4994	1
389	179	HNH endonuclease family protein	4995	1
390	448	Tetratricopeptide repeat family protein	4996	1
391	264	hypothetical protein	4997	1
392	212	Redox-sensitive transcriptional regulator (AT-rich DNA-binding protein)	4998	1
393	170	hypothetical protein	4999	1
394	820	Fibronectin type III domain protein	5000	1
395	316	Octaprenyl diphosphate synthase (EC 2.5.1.90)	5001	1
396	420	NAD-specific glutamate dehydrogenase (EC 1.4.1.2); NADP-specific glutamate dehydrogenase (EC 1.4	5002	1
397	425	NAD-specific glutamate dehydrogenase (EC 1.4.1.2); NADP-specific glutamate dehydrogenase (EC 1.4	5003	1
398	177	FIG01181331: hypothetical protein	5004	1
399	297	D-3-phosphoglycerate dehydrogenase (EC 1.1.1.95)	5005	1
400	74	FIG01181134: hypothetical protein	5006	1
401	341	Deoxyhypusine synthase (EC 2.5.1.46)	5007	1
402	946	FIG01181199: hypothetical protein	5008	1
403	273	Menaquinone via futalosine step 4	5009	1
404	205	Transcriptional regulator, Crp/Fnr family	5010	1
405	484	FIG01181108: hypothetical protein	5011	1
406	356	Stage II sporulation protein D	5012	1
407	148	Methylated-DNA--protein-cysteine methyltransferase (EC 2.1.1.63)	5013	1
408	495	Maltodextrin glucosidase (EC 3.2.1.20)	5014	1

409	249	Guanylate kinase (EC 2.7.4.8)	5015	1
410	100	DNA-directed RNA polymerase omega subunit (EC 2.7.7.6)	5016	1
411	397	Phosphopantothenoylcysteine decarboxylase (EC 4.1.1.36) / Phosphopantothenoylcysteine synthetas	5017	1
412	159	Arginine pathway regulatory protein ArgR, repressor of arg regulon	5018	1
413	203	Multiple antibiotic resistance protein marC	5019	1
414	61	hypothetical protein	5020	1
415	521	DNA repair protein RecN	5021	1
416	83	hypothetical protein	5022	1
417	135	hypothetical protein	5023	1
418	408	Serine hydroxymethyltransferase (EC 2.1.2.1)	5024	1
419	72	hypothetical protein	5025	1
420	464	Amidophosphoribosyltransferase (EC 2.4.2.14)	5026	1
421	726	Phosphoribosylformylglycinamidine synthase, synthetase subunit (EC 6.3.5.3)	5027	1
423	238	2-haloalkanoic acid dehalogenase-related protein	5029	1
424	228	Phosphoribosylformylglycinamidine synthase, glutamine amidotransferase subunit (EC 6.3.5.3)	5030	1
425	85	Phosphoribosylformylglycinamidine synthase, PurS subunit (EC 6.3.5.3)	5031	1
426	229	Phosphoribosylaminoimidazole-succinocarboxamide synthase (EC 6.3.2.6)	5032	1
427	122	FIG01198285: hypothetical protein	5033	1
428	76	Ssl5025 protein	5034	1
429	450	Adenylosuccinate lyase (EC 4.3.2.2)	5035	1
430	99	Putative nucleotidyltransferase	5036	1
431	105	protein of unknown function DUF86	5037	1
432	194	Putative dioxygenase	5038	1
433	183	Glr1707 protein	5039	1
434	220	FIG01181426: hypothetical protein	5040	1
435	122	4-carboxymuconolactone decarboxylase (EC 4.1.1.44)	5041	1
436	225	Uroporphyrinogen-III synthase (EC 4.2.1.75)	5042	1
437	393	Glutamyl-tRNA reductase (EC 1.2.1.70)	5043	1
438	251	FIG01181158: hypothetical protein	5044	1
439	66	hypothetical protein	5045	1
440	218	hypothetical protein	5046	1
441	124	conserved hypothetical protein	5047	1
442	89	hypothetical protein	5048	1
443	124	FIG01181034: hypothetical protein	5049	1
444	469	Sensor histidine kinase	5050	1
445	231	Two-component response regulator	5051	1
446	70	hypothetical protein	5052	1
447	95	Phosphoenolpyruvate synthase (EC 2.7.9.2)	5053	1
448	297	MoxR-like ATPase	5054	1
449	659	Translation elongation factor G-related protein	5055	1
450	125	FIG01181343: hypothetical protein	5056	1
451	160	Flavin reductase domain protein, FMN-binding	5057	1
452	264	Inositol-1-monophosphatase (EC 3.1.3.25)	5058	1
453	205	Phosphoribosylanthranilate isomerase (EC 5.3.1.24)	5059	1
454	1769	Ribonucleotide reductase of class II (coenzyme B12-dependent) (EC 1.17.4.1) @ intein-containing	5060	1
455	439	tRNA-t(6)A37 methylthiotransferase	5061	1
456	106	Purine nucleoside phosphoramidase YcfF/hinT protein	5062	1
457	143	FIG01165808: hypothetical protein	5063	1
458	174	Adenine phosphoribosyltransferase (EC 2.4.2.7)	5064	1
459	176	Adenine phosphoribosyltransferase (EC 2.4.2.7)	5065	1
460	453	hypothetical protein	5066	1
461	202	Hydrolase	5067	1
462	174	5-formyltetrahydrofolate cyclo-ligase (EC 6.3.3.2)	5068	1
463	85	FIG01180895: hypothetical protein	5069	1
464	158	FIG01181825: hypothetical protein	5070	1
465	179	FIG01181205: hypothetical protein	5071	1
466	202	Thymidylate kinase (EC 2.7.4.9)	5072	1
467	243	FIG137478: Hypothetical protein YbgI	5073	1
468	601	Acylamino-acid-releasing enzyme (EC 3.4.19.1)	5074	1
469	89	FIG01181597: hypothetical protein	5075	1
470	116	FIG01181262: hypothetical protein	5076	1
471	110	Putative uncharacterized protein TTHA1602	5077	1
472	125	FIG01180944: hypothetical protein	5078	1
473	195	Recombination protein RecR	5079	1
474	106	FIG000557: hypothetical protein co-occurring with RecR	5080	1
475	327	Porphobilinogen synthase (EC 4.2.1.24)	5081	1
476	398	putative permease	5082	1
477	571	FIG01181169: hypothetical protein	5084	1

478	252	Protein phosphatase 2C (EC 3.1.3.16)	5085	1
479	652	Serine/threonine protein kinase	5086	1
480	215	Probable two-component response regulator	5087	1
481	434	Sensor histidine kinase	5088	1
482	192	FIG01180896: hypothetical protein	5089	1
483	139	FIG01181488: hypothetical protein	5090	1
484	738	FIG01181361: hypothetical protein	5091	1
485	869	ATP-dependent DNA helicase UvrD/PcrA	5093	1
486	129	SSU ribosomal protein S9p (S16e)	5094	1
487	142	LSU ribosomal protein L13p (L13Ae)	5095	1
488	550	CTP synthase (EC 6.3.4.2)	5096	1
489	342	hypothetical protein	5097	1
490	204	Thiamin pyrophosphokinase (EC 2.7.6.2)	5098	1
491	317	Thiamin ABC transporter, ATPase component	5099	1
492	149	Cytochrome c-552 precursor	5100	1
493	139	Thioredoxin	5101	1
494	140	Sulfur oxidation protein SoxY	5102	1
495	109	Sulfur oxidation protein SoxZ	5103	1
496	190	Sulfur oxidation protein SoxX	5104	1
497	265	Sulfur oxidation protein SoxA	5105	1
498	144	Putative transferase/hydrolase	5106	1
499	284	FIG01181675: hypothetical protein	5107	1
500	428	Sulfide dehydrogenase [flavocytochrome C] flavoprotein chain precursor (EC 1.8.2.-)	5108	1
501	428	Sulfur oxidation molybdopterin C protein	5109	1
502	183	Sulfite dehydrogenase cytochrome subunit SoxD	5110	1
503	223	Cytochrome c-type biogenesis protein CcdA (DsbD analog)	5111	1
504	190	ABC transporter involved in cytochrome c biogenesis, ATPase component CcmA	5112	1
505	222	ABC transporter involved in cytochrome c biogenesis, CcmB subunit	5113	1
506	229	Cytochrome c-type biogenesis protein CcmC, putative heme lyase for CcmE	5114	1
507	143	Cytochrome c-type biogenesis protein CcmE, heme chaperone	5116	1
508	644	Cytochrome c heme lyase subunit CcmF	5117	1
509	179	Cytochrome c-type biogenesis protein CcmG/DsbE, thiol:disulfide oxidoreductase	5118	1
510	141	Cytochrome c heme lyase subunit CcmL	5119	1
511	327	Cytochrome c family protein	5120	1
512	185	Cytochrome complex Fe-S subunit, putative	5121	1
513	279	hypothetical protein	5122	1
514	434	Tyrosyl-tRNA synthetase (EC 6.1.1.1)	5123	1
515	126	FIG01181798: hypothetical protein	5124	1
516	98	SSU ribosomal protein S20p	5125	1
517	246	FIG01181033: hypothetical protein	5126	1
518	293	Homoserine kinase (EC 2.7.1.39)	5127	1
519	123	hypothetical protein	5129	1
520	481	NAD(P)HX epimerase / NAD(P)HX dehydratase	5130	1
521	245	NAD-dependent protein deacetylase of SIR2 family	5131	1
522	528	Transposase, IS605 family, OrfB	5132	1
523	264	hypothetical protein	5133	1
524	157	hypothetical protein	5134	1
525	433	GTP-binding protein EngA	5135	1
526	194	Acyltransferase family protein	5136	1
527	121	FIG01180845: hypothetical protein	5137	1
528	125	Holo-[acyl-carrier protein] synthase (EC 2.7.8.7)	5138	1
529	456	tRNA and rRNA cytosine-C5-methylases	5139	1
530	350	3-dehydroquinate synthase (EC 4.2.3.4)	5140	1
531	185	Shikimate kinase I (EC 2.7.1.71)	5141	1
532	396	Chorismate synthase (EC 4.2.3.5)	5142	1
533	757	Type IV pilus biogenesis protein PilQ / Competence protein PilQ	5143	1
534	310	Invasin	5144	1
535	200	Type IV pilus biogenesis protein PilO; Competence protein PilO	5145	1
536	208	Competence protein PilN	5146	1
537	378	Type IV pilus biogenesis protein PilM / Competence protein PilM	5147	1
538	335	Homoisocitrate dehydrogenase (EC 1.1.1.87)	5148	1
539	152	FIG01181708: hypothetical protein	5149	1
540	474	HD-GYP domain	5150	1
541	122	FIG01181436: hypothetical protein	5152	1
542	253	Pantothenate kinase type III, CoaX-like (EC 2.7.1.33)	5153	1
543	134	OsmC/Ohr family protein	5154	1
544	365	N-acetylmuramoyl-L-alanine amidase (EC 3.5.1.28)	5155	1
545	258	Protein kinase	5156	1

546	251	UPF0028 protein YchK	5157	1
547	269	Signal peptidase I (EC 3.4.21.89)	5158	1
548	188	Predicted L-lactate dehydrogenase, hypothetical protein subunit YkgG	5159	1
549	467	Predicted L-lactate dehydrogenase, Iron-sulfur cluster-binding subunit YkgF	5160	1
550	237	Predicted L-lactate dehydrogenase, Fe-S oxidoreductase subunit YkgE	5161	1
551	39	hypothetical protein		1
552	415	Sensory transduction histidine kinase	5162	1
553	226	Two-component response regulator	5163	1
554	120	FIG01181502: hypothetical protein	5164	1
555	216	Transporter	5165	1
556	203	Transcriptional regulator, Crp/Fnr family	5166	1
557	69	FIG01181107: hypothetical protein	5167	1
558	141	FIG01181393: hypothetical protein	5168	1
559	104	Periplasmic divalent cation tolerance protein cutA	5169	1
560	806	DNA gyrase subunit A (EC 5.99.1.3)	5170	1
561	150	3-dehydroquinate dehydratase II (EC 4.2.1.10)	5171	1
562	322	Peptidase M23B	5172	1
563	405	tRNA (uracil(54)-C5)-methyltransferase (EC 2.1.1.35)	5173	1
564	218	DNA-binding response regulator, LuxR family	5174	1
565	330	Putative peptidase	5175	1
566	277	FIG00789753: hypothetical protein	5176	1
567	580	ABC transporter, ATP-binding protein	5177	1
568	601	Lipid A export ATP-binding/permease protein MsbA	5178	1
569	395	Dihydrofolate synthase (EC 6.3.2.12) @ Folylpolyglutamate synthase (EC 6.3.2.17)	5179	1
570	348	FIG01181705: hypothetical protein	5180	1
571	340	Dipeptide transport system permease protein DppC (TC 3.A.1.5.2)	5181	1
572	307	Dipeptide transport system permease protein DppB (TC 3.A.1.5.2)	5182	1
573	502	Oligopeptide ABC transporter, periplasmic oligopeptide-binding protein OppA (TC 3.A.1.5.1)	5183	1
574	238	Branched-chain amino acid transport ATP-binding protein LivF (TC 3.A.1.4.1)	5184	1
575	260	Branched-chain amino acid transport ATP-binding protein LivG (TC 3.A.1.4.1)	5185	1
576	443	Branched-chain amino acid transport system permease protein LivM (TC 3.A.1.4.1)	5186	1
577	323	High-affinity branched-chain amino acid transport system permease protein LivH (TC 3.A.1.4.1)	5187	1
578	390	Branched-chain amino acid ABC transporter, amino acid-binding protein (TC 3.A.1.4.1)	5188	1
579	447	Glutamine synthetase type I (EC 6.3.1.2)	3771	9
580	551	5'-nucleotidase (EC 3.1.3.5)	3772	9
581	148	FIG01181496: hypothetical protein	3773	9
582	232	Cytochrome c-552 precursor	3774	9
583	408	probable sulfite reductase	3775	9
584	812	DNA mismatch repair protein MutS	3776	9
585	533	DNA mismatch repair protein MutL	3777	9
586	286	Formyltetrahydrofolate deformylase (EC 3.5.1.10)	3778	9
587	405	protease Do	3779	9
588	139	FIG01180967: hypothetical protein	3780	9
589	326	TRAP transporter solute receptor, TAXI family precursor	3781	9
590	715	TRAP-type uncharacterized transport system, fused permease component	3782	9
591	335	TRAP transporter solute receptor, TAXI family precursor	3783	9
592	371	Putative zinc metalloprotease MJ0392 (EC 3.4.24.-)	3784	9
593	399	Putative integral membrane efflux protein	3785	9
594	373	UDP-N-acetylglucosamine 2-epimerase (EC 5.1.3.14)	3786	9
595	186	COG1355, Predicted dioxygenase	3787	9
596	363	Undecaprenyl-phosphate N-acetylglucosaminyl 1-phosphate transferase (EC 2.7.8.-)	3788	9
597	209	Uracil phosphoribosyltransferase (EC 2.4.2.9)	3789	9
598	281	FIG01181440: hypothetical protein	3790	9
599	358	Hypothetical radical SAM family enzyme in heat shock gene cluster, similarity with CPO of BS HemN-t	3791	9
600	985	Putative type IIS restriction /modification enzyme, N-terminal half	3792	9
601	62	hypothetical conserved protein	3793	9
602	521	Malate synthase (EC 2.3.3.9)	3794	9
603	253	Transcriptional regulator, IclR family	3795	9
604	226	Probable transcriptional regulator, merR family	3796	9
605	437	Acetylornithine deacetylase/Succinyl-diaminopimelate desuccinylase and related deacylases	3797	9
606	248	Probable 2-phosphosulfolactate phosphatase (EC 3.1.3.71)	3798	9
607	216	cAMP-binding proteins - catabolite gene activator and regulatory subunit of cAMP-dependent protei	3799	9
608	209	Lipoprotein releasing system ATP-binding protein LolD	3800	9
609	236	Purine nucleoside phosphorylase (EC 2.4.2.1)	3801	9
610	272	Enoyl-CoA hydratase (EC 4.2.1.17)	3802	9
611	291	FIG01180926: hypothetical protein	3803	9
612	543	Mobile element protein	3804	9
613	54	hypothetical protein	3805	9

614	42	hypothetical protein	3806	9
615	318	FIG01181005: hypothetical protein	3807	9
616	298	Phosphoglucosamine mutase (EC 5.4.2.10)	3808	9
617	702	ATP-dependent DNA helicase UvrD/PcrA	3809	9
618	238	FIG01181124: hypothetical protein	3810	9
619	141	Disulfide bond formation protein B	3811	9
620	49	hypothetical protein	3812	9
621	305	Signal recognition particle receptor protein FtsY (alpha subunit) (TC 3.A.5.1.1)	3813	9
622	159	FIG01181749: hypothetical protein	3814	9
623	469	Glutamyl-tRNA synthetase (EC 6.1.1.17) @ Glutamyl-tRNA(Gln) synthetase (EC 6.1.1.24)	3815	9
624	243	Lipopolysaccharide ABC transporter, ATP-binding protein LptB	3816	9
625	463	Signal recognition particle receptor protein FtsY (alpha subunit) (TC 3.A.5.1.1)	3817	9
626	273	Modification methylase, hemK family	3818	9
627	196	RNA-binding protein Jag	3819	9
628	431	Inner membrane protein translocase component YidC, long form	3820	9
629	83	Protein YidD	3821	9
630	80	Ribonuclease P protein component (EC 3.1.26.5)	3822	9
631	49	LSU ribosomal protein L34p	3823	9
632	254	Branched-chain amino acid transport ATP-binding protein LivG (TC 3.A.1.4.1)	3824	9
633	645	Long-chain-fatty-acid--CoA ligase (EC 6.2.1.3)	3825	9
634	298	High-affinity branched-chain amino acid transport system permease protein LivH (TC 3.A.1.4.1)	3826	9
635	356	Branched-chain amino acid transport system permease protein LivM (TC 3.A.1.4.1)	3827	9
636	399	High-affinity leucine-specific transport system, periplasmic binding protein LivK (TC 3.A.1.4.1)	3829	9
637	278	Branched-chain amino acid transport ATP-binding protein LivF (TC 3.A.1.4.1)	3830	9
638	246	3-oxoacyl-[acyl-carrier protein] reductase (EC 1.1.1.100)	3831	9
639	306	Malonyl CoA-acyl carrier protein transacylase (EC 2.3.1.39)	3832	9
640	323	3-oxoacyl-[acyl-carrier-protein] synthase, KASIII (EC 2.3.1.180)	3833	9
641	61	LSU ribosomal protein L32p @ LSU ribosomal protein L32p, zinc-dependent	3834	9
642	174	COG1399 protein, clustered with ribosomal protein L32p	3835	9
643	289	Arginase (EC 3.5.3.1)	3836	9
644	543	Mobile element protein	3837	9
645	388	hypothetical protein	3838	9
646	326	hypothetical protein	3839	9
647	250	Carboxymethylenebutenolidase-related protein	3840	9
648	1102	Acriflavin resistance protein B	3841	9
649	393	FIG01181308: hypothetical protein	3842	9
650	321	Chromosome partition protein smc	3843	9
651	422	Outer membrane protein TolC, putative	3844	9
652	145	Transcriptional regulator, MarR family	3845	9
653	431	Magnesium and cobalt efflux protein CorC	3846	9
654	123	Cytidine deaminase (EC 3.5.4.5)	3847	9
655	220	FIG01181290: hypothetical protein	3848	9
656	284	Dihydrodipicolinate synthase	3849	9
657	637	FIG01180865: hypothetical protein	3850	9
658	221	L-serine dehydratase, beta subunit (EC 4.3.1.17)	3851	9
659	325	Sensory transduction protein kinase	3852	9
660	611	Translation elongation factor LepA	3853	9
661	737	Protein-export membrane protein SecD (TC 3.A.5.1.1) / Protein-export membrane protein SecF (TC 3	3854	9
662	97	FIG01181367: hypothetical protein	3855	9
663	572	Translation initiation factor 2	3856	9
664	92	COG2740: Predicted nucleic-acid-binding protein implicated in transcription termination	3857	9
665	386	Transcription termination protein NusA	3858	9
666	142	FIG000325: clustered with transcription termination protein NusA	3859	9
667	78	hypothetical protein	3860	9
668	522	Transposase, IS605 family, OrfB	3861	9
669	384	Diaminohydroxyphosphoribosylaminopyrimidine deaminase (EC 3.5.4.26) / 5-amino-6-(5-phosphorib	3862	9
670	195	Riboflavin synthase eubacterial/eukaryotic (EC 2.5.1.9)	3863	9
671	400	3,4-dihydroxy-2-butanone 4-phosphate synthase (EC 4.1.99.12) / GTP cyclohydrolase II (EC 3.5.4.25)	3864	9
672	707	FIG01181385: hypothetical protein	3865	9
673	118	hypothetical protein	3866	9
674	156	Thioesterase family protein	3867	9
675	143	B12 binding domain of Methylmalonyl-CoA mutase (EC 5.4.99.2)	3868	9
676	552	Methylmalonyl-CoA mutase (EC 5.4.99.2)	3869	9
677	289	Cobalt-zinc-cadmium resistance protein	3870	9
678	493	Lysyl-tRNA synthetase (class II) (EC 6.1.1.6)	3871	9
679	157	Anti-cleavage anti-GreA transcription factor Gfh1	3872	9
680	121	FIG01181664: hypothetical protein	3873	9
681	116	Diacylglycerol kinase (EC 2.7.1.107)	3874	9

682	139	Metal-dependent hydrolase YbeY, involved in rRNA and/or ribosome maturation and assembly	3875	9
683	678	Phosphate starvation-inducible protein PhoH, predicted ATPase	3876	9
684	131	FIG01181037: hypothetical protein	3877	9
685	326	hypothetical protein	3878	9
686	423	FIG01181387: hypothetical protein	3879	9
687	266	Shikimate 5-dehydrogenase I alpha (EC 1.1.1.25)	3880	9
688	833	DNA polymerase I (EC 2.7.7.7)	3881	9
689	396	FIG01180940: hypothetical protein	3401	10
690	437	Phosphoglucosamine mutase (EC 5.4.2.10)	3402	10
691	95	Ribosome-binding factor A	3403	10
692	133	4-hydroxybenzoyl-CoA thioesterase family active site	3404	10
693	415	Acetylornithine aminotransferase (EC 2.6.1.11)	3405	10
694	738	3'-to-5' exoribonuclease RNase R	3406	10
695	371	FIG01181349: hypothetical protein	3407	10
696	439	tRNA nucleotidyltransferase, CC-adding (EC 2.7.7.21)	3408	10
697	201	Dephospho-CoA kinase (EC 2.7.1.24)	3409	10
698	228	Short-chain dehydrogenase/reductase SDR	3410	10
699	136	Sulfur acceptor protein SufE for iron-sulfur cluster assembly	3411	10
700	180	Acyl-phosphate:glycerol-3-phosphate O-acyltransferase PlsY	3412	10
701	475	Argininosuccinate lyase (EC 4.3.2.1)	3413	10
702	311	UDP-glucose 4-epimerase (EC 5.1.3.2)	3415	10
703	189	Alkyl hydroperoxide reductase and/or thiol-specific antioxidant family (AhpC/TSA) protein	3416	10
704	805	Lead, cadmium, zinc and mercury transporting ATPase (EC 3.6.3.3) (EC 3.6.3.5); Copper-translocating	3417	10
705	151	UspA domain / CBS domain protein	3418	10
706	322	Cation antiporter	3419	10
707	149	Universal stress protein family	3420	10
708	576	Ribonuclease J2 (endoribonuclease in RNA processing)	3421	10
709	714	Polyribonucleotide nucleotidyltransferase (EC 2.7.7.8)	3422	10
710	106	SSU ribosomal protein S15p (S13e)	3423	10
711	386	Putative permease	3424	10
712	182	FIG01181217: hypothetical protein	3425	10
713	564	Cytochrome c oxidase (B(O/a)3-type) chain I (EC 1.9.3.1)	3426	10
714	171	Cytochrome c oxidase (B(O/a)3-type) chain II (EC 1.9.3.1)	3427	10
715	35	Ba3-type cytochrome c oxidase polypeptide IIA	3428	10
716	197	Gliding motility protein MglA	3429	10
717	164	Gliding motility protein MglB	3430	10
718	441	Acetylornithine deacetylase/Succinyl-diaminopimelate desuccinylase and related deacylases	3431	10
719	325	Agmatinase (EC 3.5.3.11)	3432	10
720	296	Dipeptidyl aminopeptidases/acylaminoacyl-peptidase	3433	10
721	55	hypothetical protein	3435	10
722	305	FIG01181702: hypothetical protein	3436	10
723	185	Translation elongation factor P	3437	10
724	166	Biotin carboxyl carrier protein of acetyl-CoA carboxylase	3438	10
725	446	Biotin carboxylase of acetyl-CoA carboxylase (EC 6.3.4.14)	3439	10
726	111	hypothetical protein	3440	10
727	152	Transcription termination protein NusB	3441	10
728	284	Methylenetetrahydrofolate dehydrogenase (NADP+) (EC 1.5.1.5) / Methenyltetrahydrofolate cyclohy	3442	10
729	152	FIG01181723: hypothetical protein	3443	10
730	67	hypothetical protein	3444	10
731	662	Fe-S oxidoreductase	3445	10
732	177	Methyltransferase	3446	10
733	313	Ferric enterobactin esterase-related protein	3447	10
734	48	L-lactate dehydrogenase (EC 1.1.1.27)	3448	10
735	564	Transposase, IS605 family, OrfB	3449	10
736	280	Hypothetical glycine rich membrane protein DUF1517	3450	10
737	805	ATP-dependent protease La (EC 3.4.21.53) Type I	3451	10
738	369	hypothetical conserved membrane spanning protein	3452	10
739	210	FIG01180847: hypothetical protein	3453	10
740	491	FIG01181372: hypothetical protein	3454	10
741	136	hypothetical protein	3455	10
742	538	FIG01181397: hypothetical protein	3456	10
743	102	FIG01181159: hypothetical protein	3457	10
744	423	Mobile element protein	3458	10
745	53	hypothetical protein	3459	10
746	40	hypothetical protein	3460	10
747	288	Arginine/ornithine antiporter ArcD	3461	10
748	340	UDP-glucose dehydrogenase (EC 1.1.1.22)	3462	10
749	382	Membrane protein involved in the export of O-antigen and teichoic acid-like	3463	10

750	507	Glycosyl transferase, group 1 family protein	3465	10
751	450	Rxyl_1940 O-antigen polymerase	3466	10
752	397	Glycosyltransferase	3467	10
753	256	N-acetylmannosaminyltransferase (EC 2.4.1.187)	3468	10
754	350	GDP-mannose 4,6-dehydratase (EC 4.2.1.47)	3469	10
755	318	GDP-L-fucose synthetase (EC 1.1.1.271)	3470	10
756	99	hypothetical protein	3471	10
757	299	Mannose-6 phosphate isomerase	3472	10
758	467	Phosphoglucosamine mutase (EC 5.4.2.10)	3473	10
759	325	Mannose-1-phosphate guanylyltransferase (GDP) (EC 2.7.7.22)	3474	10
760	77	UDP-glucose 6-dehydrogenase (EC 1.1.1.22)	3475	10
761	495	Undecaprenyl-phosphate galactosephosphotransferase (EC 2.7.8.6)	3476	10
762	819	Exonuclease SbcC	3478	10
763	369	Glycosyltransferase (EC 2.4.1.-)	3479	10
764	106	hypothetical protein	3480	10
765	408	FIG024850: short form Mg-chelase associated protein with vWA domain	3481	10
766	128	Probable nucleotidyltransferase	3482	10
767	101	Probable nucleotidyltransferase	3483	10
768	463	Magnesium chelatase, subunit ChlI (EC 6.6.1.1)	3484	10
769	195	ATP-dependent protease HslV (EC 3.4.25.-)	3485	10
770	417	ATP-dependent Hsl protease ATP-binding subunit HslU	3486	10
771	358	TPR repeat:TPR repeat:TPR repeat precursor	3487	10
772	55	Twin-arginine translocation protein TatA	3488	10
773	417	Transposase	3489	10
774	46	hypothetical protein	3490	10
775	82	hypothetical protein	3491	10
776	238	Adenine-specific methyltransferase (EC 2.1.1.72)	3492	10
777	143	DNA repair protein RecN	3493	10
778	157	hypothetical protein	3494	10
779	111	hypothetical protein	3495	10
780	109	hypothetical protein	3496	10
781	101	hypothetical protein	3497	10
782	185	hypothetical protein	3498	10
783	422	hypothetical protein	3499	10
784	360	DNA primase (EC 2.7.7.-), phage-associated	3500	10
785	309	Phage recombination protein Bet	3501	10
786	57	hypothetical protein	3502	10
787	86	hypothetical protein	3503	10
788	64	hypothetical protein	3504	10
789	54	hypothetical protein	3505	10
790	95	hypothetical protein	3237	12
791		Dgeo_2718 hypothetical protein	3238	12
792	97	hypothetical protein	3239	12
793	40	hypothetical protein		12
794	128	hypothetical protein	3240	12
795	394	DNA-binding protein	3241	12
796	47	hypothetical protein	3242	12
797	413	Butyryl-CoA dehydrogenase (EC 1.3.99.2)	3243	12
798	385	3-ketoacyl-CoA thiolase (EC 2.3.1.16) @ Acetyl-CoA acetyltransferase (EC 2.3.1.9)	3244	12
799	243	3-hydroxyacyl-CoA dehydrogenase [isoleucine degradation] (EC 1.1.1.35)	3245	12
800	278	Alpha-methylacyl-CoA racemase (EC 5.1.99.4)	3246	12
801	862	Assimilatory nitrate reductase large subunit (EC:1.7.99.4)	3247	12
802	161	Arsenite oxidase small subunit precursor (EC 1.20.98.1)	3248	12
803	118	Arsenical resistance operon repressor	3249	12
804	490	ABC transporter ATP-binding protein	3250	12
805	350	Inositol transport system permease protein	3251	12
806	278	Nucleoside ABC transporter, permease protein 2	3252	12
807	380	Nucleoside ABC transporter, periplasmic nucleoside-binding protein	3253	12
808	433	Gamma-aminobutyrate:alpha-ketoglutarate aminotransferase (EC 2.6.1.19)	3254	12
809	183	ADP-ribose pyrophosphatase (EC 3.6.1.13)	3255	12
810	128	hypothetical protein	3256	12
811	497	IMP cyclohydrolase (EC 3.5.4.10) / Phosphoribosylaminoimidazolecarboxamide formyltransferase (EC	3257	12
812	369	Omega-(3) fatty acid desaturase	3259	12
813	153	D-tyrosyl-tRNA(Tyr) deacylase (EC 3.6.1.n1)	3260	12
814	281	Hypothetical protein DUF194, DegV family	3261	12
815	281	Hypothetical protein DUF194, DegV family	3262	12
816	40	hypothetical protein		12
817	57	Dipeptide transport system permease protein DppC (TC 3.A.1.5.2)	3263	12

818	52	Dipeptide transport system permease protein DppC (TC 3.A.1.5.2)	3264	12
819	134	Diaminopimelate decarboxylase (EC 4.1.1.20)	3265	12
820	180	Diaminopimelate decarboxylase (EC 4.1.1.20)	3266	12
821	270	N-acetylglucosamine-6-phosphate deacetylase (EC 3.5.1.25)	3267	12
822	53	hypothetical protein	3268	12
823	418	Transposase	3269	12
824	254	Lactam utilization protein LamB	3270	12
825	861	Pyruvate, phosphate dikinase (EC 2.7.9.1)	3271	12
826	640	Cobyric acid synthase (EC 6.3.5.10) / Adenosylcobinamide-phosphate guanylyltransferase (EC 2.7.7.6	3272	12
827	86	hypothetical protein	3273	12
828	110	hypothetical protein		12
829	332	L-threonine 3-O-phosphate decarboxylase (EC 4.1.1.81)	3274	12
830	364	Fructose-1,6-bisphosphatase, type V, archaeal (EC 3.1.3.11)	3275	12
831	110	Nucleotidyltransferase	3276	12
832	72	HEPN domain-containing protein	3277	12
833	285	Adenosylcobinamide-phosphate synthase (EC 6.3.1.10)	3278	12
834	173	Alpha-ribazole-5'-phosphate phosphatase (EC 3.1.3.73)	3279	12
835	330	Nicotinate-nucleotide-dimethylbenzimidazole phosphoribosyltransferase (EC 2.4.2.21)	3280	12
836	113	PlcB, ORFX, ORFP, ORFB, ORFA, ldh gene	3282	12
837	231	Cobalamin synthase (EC 2.7.8.26)	3283	12
838	188	Cob(I)alamin adenosyltransferase (EC 2.5.1.17)	3284	12
839	200	Globin domain	3285	12
840	441	Cobyrinic acid A,C-diamide synthase	3286	12
841	351	Sulfate adenylyltransferase, dissimilatory-type (EC 2.7.7.4)	3287	12
842	239	Uroporphyrinogen-III methyltransferase (EC 2.1.1.107)	3288	12
843	247	Putative sulfate permease	3289	12
844	220	Phosphoadenylyl-sulfate reductase [thioredoxin] (EC 1.8.4.8) / Adenylyl-sulfate reductase [thioredoxi	3290	12
845	217	Siroheme synthase / Precorrin-2 oxidase (EC 1.3.1.76) / Sirohydrochlorin ferrochelatase (EC 4.99.1.4)	3291	12
846	254	Uroporphyrinogen-III synthase (EC 4.2.1.75)	3292	12
847	576	Ferredoxin-sulfite reductase (EC 1.8.7.1)	3293	12
848	40	FIG01181111: hypothetical protein	3294	12
849	135	hypothetical conserved protein	3295	12
850	251	Heme ABC transporter, ATPase component HmuV	3296	12
851	325	Hemin ABC transporter, permease protein	3297	12
852	310	Heme ABC transporter, cell surface heme and hemoprotein receptor HmuT	3298	12
853	207	Thiamin-phosphate pyrophosphorylase (EC 2.5.1.3)	3299	12
854	65	Sulfur carrier protein ThiS	3300	12
855	261	Thiazole biosynthesis protein ThiG	3301	12
856	310	Glycine oxidase ThiO (EC 1.4.3.19)	3302	12
857	433	Hydroxymethylpyrimidine phosphate synthase ThiC (EC 4.1.99.17)	3303	12
858	260	Hydroxymethylpyrimidine phosphate kinase ThiD (EC 2.7.4.7)	3304	12
859	595	Glycogen debranching enzyme	3305	12
860	455	N-Acetyl-D-glucosamine ABC transport system, permease protein 2	3306	12
861	368	N-Acetyl-D-glucosamine ABC transport system, permease protein 1	3307	12
862	414	Extracellular solute-binding protein, family 1	3308	12
863	299	Ribose operon repressor	3309	12
864	349	FIG01181576: hypothetical protein	3310	12
865	244	Permease of the drug/metabolite transporter (DMT) superfamily		12
866	88	Mannose-6-phosphate isomerase (EC 5.3.1.8)	3312	12
867	447	Phosphoglucosamine mutase (EC 5.4.2.10)	3313	12
868	70	Mannose-1-phosphate guanylyltransferase (GDP) (EC 2.7.7.22)		12
869	140	Transposase	3314	12
870	224	TRAP-type uncharacterized transport system, periplasmic component-like protein	3315	12
871	627	TRAP transporter, 4TM/12TM fusion protein, unknown substrate 2	3316	12
872	81	hypothetical protein	3317	12
873	387	Butyryl-CoA dehydrogenase (EC 1.3.99.2)	3318	12
874	326	hypothetical protein	3319	12
875	117	hypothetical protein	3320	12
876	215	Phosphoglycerate mutase family protein	3321	12
877	248	Branched-chain amino acid transport ATP-binding protein LivF (TC 3.A.1.4.1)	3322	12
878	255	Branched-chain amino acid transport ATP-binding protein LivG (TC 3.A.1.4.1)	3323	12
879	554	Long-chain-fatty-acid--CoA ligase (EC 6.2.1.3)	3324	12
880	357	Branched-chain amino acid transport system permease protein LivM (TC 3.A.1.4.1)	3326	12
881	288	High-affinity branched-chain amino acid transport system permease protein LivH	3327	12
882	429	Putative branched-chain amino acid ABC transporter (substrate-binding protein)	3328	12
883	264	hypothetical protein		
884	50	hypothetical protein		
885	110	hypothetical protein		

886	112	hypothetical protein		
887	78	Prevent host death protein, Phd antitoxin		
888	100	Death on curing protein, Doc toxin		
889	414	Beta-lactamase	5188	3
890	263	3-oxoacyl-[acyl-carrier protein] reductase (EC 1.1.1.100)	5189	3
891	131	Phenylacetic acid degradation-related protein	5190	3
892	537	Medium-chain-fatty-acid--CoA ligase (EC 6.2.1.-)	5191	3
893	254	Short-chain dehydrogenase/reductase SDR	5192	3
894	136	FIG01180875: hypothetical protein	5193	3
895	543	Mobile element protein	5194	3
896	739	FIG01180875: hypothetical protein	5195	3
897	336	3-oxoacyl-[ACP] synthase III in alkane synthesis cluster	5196	3
898	495	O-succinylbenzoic acid--CoA ligase (EC 6.2.1.26)	5197	3
899	257	N-formylglutamate deformylase (EC 3.5.1.68)	5198	3
900	244	FIG01181305: hypothetical protein	5199	3
901	136	Phosphate acetyltransferase (EC 2.3.1.8)	5200	3
902	399	Leucine-, isoleucine-, valine-, threonine-, and alanine-binding protein	5202	3
903	286	High-affinity branched-chain amino acid transport system permease protein LivH (TC 3.A.1.4.1)	5203	3
904	299	Branched amino acid transport system permease	5204	3
905	242	Branched-chain amino acid transport ATP-binding protein LivG (TC 3.A.1.4.1)	5205	3
906	235	Branched-chain amino acid transport ATP-binding protein LivF (TC 3.A.1.4.1)	5206	3
907	252	D-beta-hydroxybutyrate dehydrogenase (EC 1.1.1.30)	5207	3
908	167	Phosphohistidine phosphatase SixA	5208	3
909	234	Adenylate cyclase (EC 4.6.1.1)	5209	3
910	44	Pyruvate kinase family protein	5210	3
911	605	hypothetical protein	5211	3
912	46	hypothetical protein	5212	3
913	434	Glycerol-3-phosphate ABC transporter, periplasmic glycerol-3-phosphate-binding protein (TC 3.A.1.1.	5213	3
914	271	Glycerol-3-phosphate ABC transporter, permease protein UgpE (TC 3.A.1.1.3)	5214	3
915	304	Glycerol-3-phosphate ABC transporter, permease protein UgpA (TC 3.A.1.1.3)	5215	3
916	246	Putative uncharacterized protein TTHB071	5216	3
917	244	5-nucleotidase SurE (EC 3.1.3.5)	5217	3
918	239	Probable glycosyl transferase	5218	3
919	502	Alkaline phosphatase (EC 3.1.3.1)	5219	3
920	212	Aquaporin Z	5220	3
921	136	hypothetical protein	5221	3
922	522	D-3-phosphoglycerate dehydrogenase (EC 1.1.1.95)	5222	3
923	43	hypothetical protein		3
924	143	hypothetical protein	5223	3
925	395	Branched-chain amino acid ABC transporter, amino acid-binding protein (TC 3.A.1.4.1)	5224	3
926	292	High-affinity branched-chain amino acid transport system permease protein LivH (TC 3.A.1.4.1)	5225	3
927	259	Branched-chain amino acid transport ATP-binding protein LivF (TC 3.A.1.4.1)	5226	3
928	248	Branched-chain amino acid transport ATP-binding protein LivG (TC 3.A.1.4.1)	5227	3
929	618	Long-chain-fatty-acid--CoA ligase (EC 6.2.1.3)	5228	3
930	356	Branched-chain amino acid transport system permease protein LivM (TC 3.A.1.4.1)	5229	3
931	172	FIG01181758: hypothetical protein	5230	3
932	913	hypothetical protein	5231	3
933	883	hypothetical protein	5232	3
934	1793	FIG00787995: hypothetical protein	5233	3
935	1357	Putative internalin	5234	3
936	71	hypothetical protein		3
937	192	Transcriptional regulator, TetR family	5235	3
938	379	Butyryl-CoA dehydrogenase (EC 1.3.99.2)	5236	3
939	151	MaoC-related acyl dehydratase	5237	3
940	247	3-oxoacyl-[acyl-carrier protein] reductase (EC 1.1.1.100)	5238	3
941	159	Bacterioferritin	5239	3
942	99	Transcriptional regulator, HxlR family	5240	3
943	309	DNA integration/recombination/inversion protein	5241	3
944	81	hypothetical protein	5242	3
945	63	hypothetical protein	5243	3
946	373	Fructokinase (EC 2.7.1.4)	5244	3
947	87	CRISPR-associated protein Cas2	5245	3
948	170	hypothetical protein	5246	3
949	319	CRISPR-associated protein, Csd2/Csh2 family	5247	3
950	619	CRISPR-associated protein, Csh1 family	5248	3
951	223	CRISPR repeat RNA endoribonuclease Cas6	5249	3
952	750	CRISPR-associated helicase Cas3	5250	3
953	174	CRISPR-associated RecB family exonuclease Cas4a	5251	3

954	328	CRISPR-associated protein Cas1	5252	3
955	186	Transcriptional regulator, TetR family	5253	3
956	324	Phenylacetate-CoA oxygenase, PaaG subunit	5254	3
957	174	Phenylacetate-CoA oxygenase, PaaH2 subunit	5255	3
958	253	Phenylacetate-CoA oxygenase, PaaI subunit	5256	3
959	152	Phenylacetate-CoA oxygenase, PaaJ subunit	5257	3
960	664	Phenylacetic acid degradation protein PaaN, ring-opening aldehyde dehydrogenase (EC 1.2.1.3)	5258	3
961	173	FIG01181068: hypothetical protein	5259	3
962	154	FIG01181318: hypothetical protein	5260	3
963	446	Phenylacetate--CoA ligase (EC 6.2.1.30)	5261	3
964	123	Phenylacetic acid degradation protein PaaD, thioesterase	5262	3
965	239	Branched-chain amino acid transport ATP-binding protein LivF (TC 3.A.1.4.1)	5263	3
966	583	Branched-chain amino acid transport system permease protein LivM (TC 3.A.1.4.1)	5264	3
967	326	High-affinity branched-chain amino acid transport system permease protein LivH (TC 3.A.1.4.1)	5265	3
968	391	Branched-chain amino acid ABC transporter, amino acid-binding protein (TC 3.A.1.4.1)	5266	3
969	320	3,4-dihydroxyphenylacetate 2,3-dioxygenase (EC 1.13.11.15)	5267	3
970	149	Nitrilotriacetate monooxygenase component B (EC 1.14.13.-)	5268	3
971	482	4-hydroxyphenylacetate 3-monooxygenase (EC 1.14.13.3)	5269	3
972	518	5-carboxymethyl-2-hydroxymuconate semialdehyde dehydrogenase (EC 1.2.1.60)	5270	3
973	247	5-carboxymethyl-2-oxo-hex-3- ene-1,7-dioate decarboxylase (EC 4.1.1.68) / 2-hydroxyhepta-2,4-dien	5271	3
974	313	4-hydroxy-tetrahydrodipicolinate synthase (EC 4.3.3.7)	5272	3
975	458	hypothetical conserved protein	5273	3
976	235	Uroporphyrinogen-III methyltransferase (EC 2.1.1.107)	5275	3
977	105	FIG056361: hypothetical protein implicated in coenzyme B12 biosynthesis	5276	3
978	368	Sirohydrochlorin cobaltochelatase (EC 4.99.1.3) / Putative 2Fe-2S ferredoxin CbiW involved in B12 bio	5277	3
979	360	Cobalamin biosynthesis protein CbiG	5278	3
980	288	Cobalt-precorrin-3b C17-methyltransferase	5279	3
981	248	Cobalt-precorrin-4 C11-methyltransferase (EC 2.1.1.133)	5280	3
982	226	Cobalt-precorrin-2 C20-methyltransferase (EC 2.1.1.130)	5281	3
983	405	Cobalt-precorrin-6y C5-methyltransferase (EC 2.1.1.-) / Cobalt-precorrin-6y C15-methyltransferase [d	5282	3
984	233	Cobalt-precorrin-8x methylmutase (EC 5.4.1.2)	5283	3
985	367	Cobalt-precorrin-6 synthase, anaerobic	5284	3
986	221	HoxN/HupN/NixA family cobalt transporter	5285	3
987	401	3-ketoacyl-CoA thiolase (EC 2.3.1.16) @ Acetyl-CoA acetyltransferase (EC 2.3.1.9)	5286	3
988	495	L-aspartate oxidase (EC 1.4.3.16)	5287	3
989	308	Quinolinate synthetase (EC 2.5.1.72)	5288	3
990	137	Sll1715 protein	5289	3
991	85	hypothetical protein	5290	3
992	280	Quinolinate phosphoribosyltransferase [decarboxylating] (EC 2.4.2.19)	5291	3
993	641	Thymidylate kinase (EC 2.7.4.9)	5292	3
994	495	Allophanate hydrolase 2 subunit 1 (EC 3.5.1.54) / Allophanate hydrolase 2 subunit 2 (EC 3.5.1.54)	5293	3
995	515	Acyl-CoA dehydrogenase (EC 1.3.8.7)	5294	3
996	571	Tungsten-containing aldehyde:ferredoxin oxidoreductase (EC 1.2.7.5)	5295	3
997	168	hypothetical protein	5296	3
998	137	FIG01181245: hypothetical protein	5297	3
999	555	Isopentenyl-diphosphate delta-isomerase (EC 5.3.3.2)	5298	3
1000	439	Omega-amino acid--pyruvate aminotransferase (EC 2.6.1.18)	5299	3
1001	529	FIG01180880: hypothetical protein	5300	3
1002	235	FIG01181047: hypothetical protein	5301	3
1003	55	hypothetical conserved protein	5302	3
1004	285	Zinc ABC transporter, periplasmic-binding protein ZnuA	5303	3
1005	348	Galactokinase (EC 2.7.1.6)	5304	3
1006	42	NADH-ubiquinone oxidoreductase chain F (EC 1.6.5.3)	5305	3
1007	50	NADH-ubiquinone oxidoreductase chain F (EC 1.6.5.3)		3
1008	81	Formate dehydrogenase alpha subunit (EC 1.2.1.2)	5306	3
1009	359	TRAP transporter solute receptor, unknown substrate 6	5307	3
1010	107	Two-component response regulator	5308	3
1011	959	Sensory transduction histidine kinase (EC 2.7.3.-)	5309	3
1012	382	Butyryl-CoA dehydrogenase (EC 1.3.99.2)	5311	3
1013	248	Electron transfer flavoprotein, beta subunit	5312	3
1014	314	Electron transfer flavoprotein, alpha subunit	5313	3
1015	126	FIG01181392: hypothetical protein	5314	3
1016	525	Acetyl-coenzyme A carboxyl transferase alpha chain (EC 6.4.1.2) / Acetyl-coenzyme A carboxyl transfe	5315	3
1017	224	Uracil-DNA glycosylase, family 5	5316	3
1018	576	DNA polymerase X family	5317	3
1019	83	FIG01181174: hypothetical protein	5318	3
1020	47	hypothetical protein	5320	3
1021	409	Aminopeptidase S (Leu, Val, Phe, Tyr preference) (EC 3.4.11.24)	5321	3

1022	413	O-acetylhomoserine sulfhydrylase (EC 2.5.1.49) / O-succinylhomoserine sulfhydrylase (EC 2.5.1.48)	5322	3
1023	138	Universal stress protein family	5323	3
1024	283	ABC transporter substrate-binding protein (taurine)	5324	3
1025	249	ABC transporter ATP-binding protein (taurine)	5325	3
1026	250	Hydroxymethylpyrimidine ABC transporter, transmembrane component	5326	3
1027	342	Chaperonin (heat shock protein 33)	5327	3
1028	481	Cysteinyl-tRNA synthetase (EC 6.1.1.16)	5328	3
1029	578	Acyl-CoA dehydrogenase, short-chain specific (EC 1.3.99.2)	5329	3
1030	398	3-ketoacyl-CoA thiolase (EC 2.3.1.16) @ Acetyl-CoA acetyltransferase (EC 2.3.1.9)	5330	3
1031	764	Enoyl-CoA hydratase [isoleucine degradation] (EC 4.2.1.17) / 3-hydroxyacyl-CoA dehydrogenase (EC 1	5331	3
1032	64	hypothetical protein	5333	3
1033	979	Transcription-repair coupling factor	5334	3
1034	287	Hypothetical protein DUF194, DegV family	5335	3
1035	193	Pyrrolidone-carboxylate peptidase (EC 3.4.19.3)	5336	3
1036	271	hypothetical protein	5337	3
1037	399	Glycosyltransferase	5338	3
1038	236	Glycosyltransferase	5339	3
1039	391	Multidrug resistance protein-related protein	5340	3
1040	398	Putative oligosaccharide deacetylase	5341	3
1041	223	hypothetical protein	5342	3
1042	248	FIG01181001: hypothetical protein	5343	3
1043	90	Aspartyl-tRNA(Asn) amidotransferase subunit C (EC 6.3.5.6) @ Glutamyl-tRNA(Gln) amidotransferase	5344	3
1044	425	Seryl-tRNA synthetase (EC 6.1.1.11)	5345	3
1045	370	N-acylamino acid racemase	5346	3
1046	261	FIG01181548: hypothetical protein	5347	3
1047	370	S-adenosylhomocysteine deaminase (EC 3.5.4.28); Methylthioadenosine deaminase	5348	3
1048	454	Chloride channel protein	5349	3
1049	231	LmbE-related protein	5350	3
1050	123	Chorismate mutase II (EC 5.4.99.5)	5351	3
1051	60	hypothetical protein	5352	3
1052	531	Aldehyde dehydrogenase B (EC 1.2.1.22)	5353	3
1053	162	Phosphopantetheine adenylyltransferase (EC 2.7.7.3)	5354	3
1054	171	Ribosomal RNA small subunit methyltransferase D (EC 2.1.1.-)	5355	3
1055	492	Sodium-dependent phosphate transporter	5356	3
1056	376	Transposase	5357	3
1057	435	O-acetylhomoserine sulfhydrylase (EC 2.5.1.49) / O-succinylhomoserine sulfhydrylase (EC 2.5.1.48)	5358	3
1058	381	Homoserine O-acetyltransferase (EC 2.3.1.31)	5359	3
1059	433	GTPase and tRNA-U34 5-formylation enzyme TrmE	5360	3
1060	65	hypothetical protein	5361	3
1061	84	hypothetical protein	5362	3
1062	425	Glutamate-1-semialdehyde aminotransferase (EC 5.4.3.8)	5363	3
1063	332	Acetoin dehydrogenase E1 component beta-subunit (EC 1.2.4.-)	5364	3
1064	342	Acetoin dehydrogenase E1 component alpha-subunit (EC 1.2.4.-)	5365	3
1065	373	Aspartate aminotransferase (EC 2.6.1.1)	5366	3
1066	248	Triosephosphate isomerase (EC 5.3.1.1)	5367	3
1067	535	BNR repeat domain protein	5368	3
1068	391	Phosphoglycerate kinase (EC 2.7.2.3)	5369	3
1069	332	NAD-dependent glyceraldehyde-3-phosphate dehydrogenase (EC 1.2.1.12)	5370	3
1070	203	Substrate-specific component BioY of biotin ECF transporter	5371	3
1071	264	hypothetical protein	5372	3
1072	112	FIG003879: Predicted amidohydrolase	5373	3
1073	145	tmRNA-binding protein SmpB	5374	3
1074	383	N-acetylmuramoyl-L-alanine amidase (EC 3.5.1.28)	5375	3
1075	165	FIG01180933: hypothetical protein	5376	3
1076	1008	Chromosome partition protein smc	5377	3
1077	109	FIG01181533: hypothetical protein	5378	3
1078	68	FIG01181575: hypothetical protein	5379	3
1079	228	Probable metal-dependent peptidase	5380	3
1080	43	hypothetical protein		3
1081	287	Thiosulfate sulfurtransferase, rhodanese (EC 2.8.1.1)	5381	3
1082	287	L-serine dehydratase, alpha subunit (EC 4.3.1.17)	5382	3
1083	193	DNA-binding response regulator	5383	3
1084	151	LSU ribosomal protein L19p	5384	3
1085	240	tRNA (Guanine37-N1) -methyltransferase (EC 2.1.1.31)	5385	3
1086	166	16S rRNA processing protein RimM	5386	3
1087	73	KH domain RNA binding protein YlqC	5387	3
1088	88	SSU ribosomal protein S16p	5388	3
1089	431	Signal recognition particle, subunit Ffh SRP54 (TC 3.A.5.1.1)	5389	3

1090	449	Mg/Co/Ni transporter MgtE / CBS domain	5390	3
1091	132	ADP-ribosylglycohydrolase	5391	3
1092	346	S-adenosylmethionine:tRNA ribosyltransferase-isomerase (EC 5.-.-.-)	5392	3
1093	180	FIG01181505: hypothetical protein	5393	3
1094	858	Phosphoenolpyruvate carboxylase (EC 4.1.1.31)	5394	3
1095	252	FIG006542: Phosphoesterase	5395	3
1096	318	Mobile element protein	5396	3
1097	80	FIG01181275: hypothetical protein	5397	3
1098	222	DNA recombination and repair protein RecO	5398	3
1099	157	Transcription elongation factor GreA	5399	3
1100	249	Aminodeoxychorismate lyase (EC 4.1.3.38)	5400	3
1101	617	Para-aminobenzoate synthase, aminase component (EC 2.6.1.85) / Para-aminobenzoate synthase, an	5401	3
1102	174	COG1355, Predicted dioxygenase	5402	3
1103	1186	5-methyltetrahydrofolate--homocysteine methyltransferase (EC 2.1.1.13)	5403	3
1104	508	Nicotinate phosphoribosyltransferase (EC 2.4.2.11)	5404	3
1105	397	ATP-dependent Clp protease ATP-binding subunit ClpX	5405	3
1106	195	ATP-dependent Clp protease proteolytic subunit (EC 3.4.21.92)	5406	3
1107	404	Cell division trigger factor (EC 5.2.1.8)	5407	3
1108	95	hypothetical conserved protein	5408	3
1109	1031	Carbamoyl-phosphate synthase large chain (EC 6.3.5.5)	5409	3
1110	110	Protein from nitrogen regulatory protein P-II (GLNB) family, ortholog YAAQ B. subtilis	5410	3
1111	286	FIG01181588: hypothetical protein	5411	3
1112	258	tRNA (Adenine-N(1)-) -methyltransferase (EC 2.1.1.36)	5412	3
1113	517	Fibronectin/fibrinogen-binding protein	5413	3
1114	117	Aspartate 1-decarboxylase (EC 4.1.1.11)	5414	3
1115	285	Menaquinone via futalosine polyprenyltransferase (MenA homolog)	5415	3
1116	193	Hypothetical protein ywlG	5416	3
1117	353	Soluble hydrogenase, small subunit	5417	3
1118	287	FIG01181853: hypothetical protein	5418	3
1119	125	Large-conductance mechanosensitive channel	5419	3
1120	377	Glycerol-3-phosphate ABC transporter, ATP-binding protein UgpC (TC 3.A.1.1.3)	5420	3
1121	567	FIG01181715: hypothetical protein	5422	3
1122	331	Pyruvyl-transferase	5423	3
1123	353	FIG01180954: hypothetical protein	5424	3
1124	248	DNA polymerase III alpha subunit (EC 2.7.7.7)	5425	3
1125	472	Aspartyl-tRNA(Asn) amidotransferase subunit A (EC 6.3.5.6) @ Glutamyl-tRNA(Gln) amidotransferase	5426	3
1126	303	tRNA dimethylallyltransferase (EC 2.5.1.75)	5427	3
1127	134	Probable small heat shock protein	5428	3
1128	193	Thymidine kinase (EC 2.7.1.21)	5429	3
1129	97	LSU ribosomal protein L31p @ LSU ribosomal protein L31p, zinc-dependent	5430	3
1130	**62**	**hypothetical protein**	5431	3
1131	**309**	**RNA-directed DNA polymerase (EC 2.7.7.9)**	**5432**	3
1132	**116**	**23S rRNA intervening sequence protein**	**5433**	3
1133	**382**	**Putative formylglycine-generating sulfatase enzyme**	**5434**	3
1134	**121**	**hypothetical protein**	**5435**	3
1135	**288**	**hypothetical protein**	**5437**	11
1136	**114**	**hypothetical protein**	**5438**	11
1137	**71**	**hypothetical protein**	**5439**	11
1138	**253**	**Phage tail protein**	**5440**	11
1139	**346**	**Baseplate J family protein**	**5441**	11
1140	**121**	**Putative Baseplate endolysin protein**	**5442**	11
1141	**215**	**Putative Phage protein**	**5443**	11
1142	**229**	**hypothetical protein**	**5444**	11
1143	**198**	**hypothetical protein**	**5445**	11
1144	**1044**	**Phage tail tape measure protein**	**5446**	11
1145	**209**	**Peptidase**	**5447**	11
1146	**174**	**hypothetical protein**	**5448**	11
1147	**94**	**hypothetical protein**	**5449**	11
1148	**390**	**Phage (Mu-like) head morphogenesis protein**	**5450**	11
1149	**503**	**Mu-like prophage portal protein gp29**	**5451**	11
1150	**426**	**Mu-like prophage terminase large subunit protein gp28**	**5452**	11
1151	**180**	**Mu-like prophage terminase small subunit protein gp27**	**5453**	11
1152	**61**	**hypothetical protein**	**5454**	11
1153	**92**	**hypothetical protein**	**5455**	11
1154	**86**	**hypothetical protein**	**5456**	11
1155	**151**	**hypothetical protein**	**5457**	11
1156	**285**	**transcriptional regulator, IclR family**	**5458**	11
1157	**63**	**hypothetical protein**	**5459**	11

1158	**88**	**hypothetical protein**	**5460**	11
1159	**75**	**hypothetical protein**	**5461**	11
1160	**127**	**hypothetical protein**	**5462**	11
1161	**133**	**DNA binding domain protein, excisionase family**	**5463**	11
1162	**81**	**hypothetical protein**	**5464**	11
1163	**92**	**hypothetical protein**	**5465**	11
1164	**95**	**hypothetical protein**	**5466**	11
1165	**63**	**hypothetical protein**	**5467**	11
1166	**141**	**hypothetical protein**	**5468**	11
1167	**204**	**hypothetical protein**	**5469**	11
1168	**257**	**AAA ATPase**	**5470**	11
1169	**650**	**Integrase catalytic region**	**5471**	11
1170	88	**Helix-turn-helix domain protein**	**5472**	11
1171	**234**	**Transcriptional regulator, XRE family**	**5473**	11
1172	**90**	**hypothetical protein**	**5474**	11
1173	**334**	**Mu-like prophage protein gp32**	**5475**	11
1174	**128**	**Phage protein**	**5476**	11
1175	**299**	**Mu-like prophage major head subunit gpT-like protein**	**5477**	11
1176	**146**	**Mu-like prophage protein gp36**	**5478**	11
1177	**162**	**Phage virion morphogenesis protein**	**5479**	11
1178	**158**	**hypothetical protein**	**5480**	11
1179	**70**	**hypothetical protein**	**5481**	11
1180	**474**	**Putative phage tail protein**	**5482**	11
1181	**138**	**Putative phage tail protein**	**5483**	11
1182	**83**	**hypothetical protein**		11
1183	**41**	**hypothetical protein**	**5484**	11
1184	**47**	**hypothetical protein**	**5485**	11
1185	145	Cell division protein MraZ	5486	11
1186	287	rRNA small subunit methyltransferase H	5487	11
1187	82	Cell division protein FtsL	5488	11
1188	440	Cell division protein FtsI [Peptidoglycan synthetase] (EC 2.4.1.129)	5489	11
1189	421	UDP-N-acetylmuramoylalanyl-D-glutamyl-2,6-diaminopimelate--D-alanyl-D-alanine ligase (EC 6.3.2.1(	5490	11
1190	267	FIG01181245: hypothetical protein	5491	11
1191	284	Phospho-N-acetylmuramoyl-pentapeptide-transferase (EC 2.7.8.13)	5492	11
1192	419	UDP-N-acetylmuramoylalanine--D-glutamate ligase (EC 6.3.2.9)	5493	11
1193	353	Cell division protein FtsW	5494	11
1194	340	UDP-N-acetylglucosamine--N-acetylmuramyl-(pentapeptide) pyrophosphoryl-undecaprenol N-acetylg	5495	11
1195	447	UDP-N-acetylmuramate--alanine ligase (EC 6.3.2.8)	5496	11
1196	261	UDP-N-acetylenolpyruvoylglucosamine reductase (EC 1.1.1.158)	5497	11
1197	193	Cell division protein FtsQ	5498	11
1198	399	Cell division protein FtsA	5499	11
1199	352	Cell division protein FtsZ (EC 3.4.24.-)	5500	11
1200	167	Crossover junction endodeoxyribonuclease RuvC (EC 3.1.22.4)	5501	11
1201	208	DNA-binding response regulator, LuxR family	5502	11
1202	560	Sensor histidine kinase	5503	11
1203	278	Phosphonate ABC transporter phosphate-binding periplasmic component (TC 3.A.1.9.1)	5504	11
1204	278	Molybdopterin oxidoreductase iron-sulfur binding subunit	5505	11
1205	1062	Tetrathionate reductase subunit A	5506	11
1206	146	hypothetical protein	5507	11
1207	148	hypothetical protein	5508	11
1208	185	hypothetical protein	5509	11
1209	402	hypothetical protein	5510	11
1210	288	NADH-ubiquinone oxidoreductase 39 kDa subunit related protein	5511	11
1211	196	Regulatory protein	5512	11
1212	284	Transcriptional regulator, MerR family	5513	11
1213	284	Phytoene synthase (EC 2.5.1.32)	5514	11
1214	418	Deoxyribodipyrimidine photolyase (EC 4.1.99.3)	5515	11
1215	60	hypothetical protein	5516	11
1216	391	Cytochrome P450	5517	11
1217	233	Geranylgeranyl-diphosphate geranylgeranyl-transferase	5518	11
1218	221	FIG01181141: hypothetical protein	5519	11
1219	335	Glycosyl transferase, family 2 precursor	5521	11
1220	523	Phytoene desaturase, neurosporene or lycopene producing (EC 1.3.-.-)	5522	11
1221	336	Isopentenyl-diphosphate delta-isomerase, FMN-dependent (EC 5.3.3.2)	5523	11
1222	321	COG1683: Uncharacterized conserved protein / FIG143828: Hypothetical protein YbgA	5524	11
1223	203	hypothetical protein	5525	11
1224	256	hypothetical protein	5526	11
1225	199	hypothetical protein	5527	11

1226	815	hypothetical protein	5528	11
1227	294	FIG003003: hypothetical protein	5529	11
1228	526	Gamma-glutamyltranspeptidase (EC 2.3.2.2)	5530	11
1229	220	Mn-dependent transcriptional regulator MntR	5531	11
1230	264	hypothetical protein	5532	11
1231	392	Predicted beta-glucoside-regulated ABC transport system, sugar-binding protein	5533	11
1232	338	Vitamin B12 ABC transporter, B12-binding component BtuF	5534	11
1233	343	Iron(III) dicitrate transport system permease protein FecD (TC 3.A.1.14.1)	5535	11
1234	251	Iron(III) dicitrate transport ATP-binding protein FecE (TC 3.A.1.14.1)	5536	11
1235	110	FIG01181091: hypothetical protein	5537	11
1236	69	hypothetical protein	5538	11
1237	389	Beta-lactamase class C and other penicillin binding proteins	5539	11
1238	291	Low-specificity L-threonine aldolase (EC 4.1.2.48)	5540	11
1239	264	hypothetical protein	5541	11
1240	63	Predicted beta-glucoside-regulated ABC transport system, permease component 2, COG0395	3507	8
1241	291	Quinone oxidoreductase (EC 1.6.5.5)	3508	8
1242	281	Ornithine cyclodeaminase (EC 4.3.1.12)	3509	8
1243	298	Zinc transport protein ZntB	3510	8
1244	129	FIG01181200: hypothetical protein	3511	8
1245	229	Ribosomal RNA small subunit methyltransferase E (EC 2.1.1.-)	3512	8
1246	253	Ribosomal protein L11 methyltransferase (EC 2.1.1.-)	3513	8
1247	199	Iron sulfur cluster regulator SufR	3514	8
1248	351	Scaffold protein for [4Fe-4S] cluster assembly, MRP-like, similar to chloroplast-targeted plant protein	3515	8
1249	201	FIG01181424: hypothetical protein	3516	8
1250	64	hypothetical protein	3517	8
1251	863	Valyl-tRNA synthetase (EC 6.1.1.9)	3518	8
1252	188	FIG01180872: hypothetical protein	3519	8
1253	666	Single-stranded-DNA-specific exonuclease RecJ (EC 3.1.-.-)	3520	8
1254	105	hypothetical protein	3521	8
1255	236	Phosphoribosylformimino-5-aminoimidazole carboxamide ribotide isomerase (EC 5.3.1.16)	3522	8
1256	255	Indole-3-glycerol phosphate synthase (EC 4.1.1.48)	3523	8
1257	284	UPF0028 protein YchK	3524	8
1258	245	Amino acid ABC transporter, ATP-binding protein	3526	8
1259	651	TRAP-type uncharacterized transport system, fused permease component	3527	8
1260	317	TRAP transporter solute receptor, TAXI family precursor	3528	8
1261	264	Amino acid ABC transporter, permease protein	3529	8
1262	262	Lysine-arginine-ornithine-binding periplasmic protein precursor (TC 3.A.1.3.1)	3530	8
1263	819	Glycogen phosphorylase (EC 2.4.1.1)	3531	8
1264	491	Trk system potassium uptake protein TrkG	3532	8
1265	446	Trk system potassium uptake protein TrkA	3533	8
1266	435	Ribosomal protein S12p Asp88 (E. coli) methylthiotransferase	3534	8
1267	288	FIG00788033: hypothetical protein	3535	8
1268	312	Indigoidine synthase A-like protein, uncharacterized enzyme involved in pigment biosynthesis	3536	8
1269	248	FIG01181281: hypothetical protein	3537	8
1270	257	Ribulosamine/erythrulosamine 3-kinase potentially involved in protein deglycation	3538	8
1271	165	Low molecular weight protein tyrosine phosphatase (EC 3.1.3.48)	3539	8
1272	516	Sensor histidine kinase	3540	8
1273	245	tRNA:Cm32/Um32 methyltransferase	3541	8
1274	743	Multimodular transpeptidase-transglycosylase (EC 2.4.1.129) (EC 3.4.-.-)	3542	8
1275	125	Response regulator	3543	8
1276	369	GTP-binding and nucleic acid-binding protein YchF	3544	8
1277	220	Deoxyribose-phosphate aldolase (EC 4.1.2.4)	3545	8
1278	380	FIG01181132: hypothetical protein	3546	8
1279	187	Septum formation protein Maf	3547	8
1280	263	Rod shape-determining protein MreC	3548	8
1281	149	Rod shape-determining protein MreD	3549	8
1282	577	Cell division protein FtsI [Peptidoglycan synthetase] (EC 2.4.1.129)	3550	8
1283	176	Septum site-determining protein MinC	3551	8
1284	146	FIG01181402: hypothetical protein	3552	8
1285	92	FIG01181522: hypothetical protein	3553	8
1286	190	FIG01181437: hypothetical protein	3554	8
1287	234	hypothetical protein	3555	8
1288	382	Glutamate N-acetyltransferase (EC 2.3.1.35) / N-acetylglutamate synthase (EC 2.3.1.1)	3557	8
1289	349	N-acetyl-gamma-glutamyl-phosphate reductase (EC 1.2.1.38)	3558	8
1290	174	COG1355, Predicted dioxygenase	3559	8
1291	302	Ornithine carbamoyltransferase (EC 2.1.3.3)	3560	8
1292	228	LMBE-related protein	3561	8
1293	237	Acyl-phosphate:glycerol-3-phosphate O-acyltransferase PlsY	3562	8

1294	52	hypothetical protein	3563	8
1295	111	hypothetical protein	3564	8
1296	106	hypothetical protein	3565	8
1297	624	hypothetical conserved protein	3566	8
1298	259	Conserved membrane protein, multidrug efflux associated	3567	8
1299	333	ABC transporter ATP-binding protein	3568	8
1300	259	Alr0191 protein	3569	8
1301	175	COG1355, Predicted dioxygenase	3570	8
1302	527	(R)-citramalate synthase (EC 2.3.1.182)	3571	8
1303	174	GCN5-related N-acetyltransferase	3572	8
1304	521	2-isopropylmalate synthase (EC 2.3.3.13)	3573	8
1305	338	Ketol-acid reductoisomerase (EC 1.1.1.86)	3574	8
1306	171	Acetolactate synthase small subunit (EC 2.2.1.6)	3575	8
1307	562	Acetolactate synthase large subunit (EC 2.2.1.6)	3576	8
1308	324	Thioredoxin reductase (EC 1.8.1.9)	3577	8
1309	157	Integral membrane protein TerC	3578	8
1310	467	Fumarate hydratase class II (EC 4.2.1.2)	3579	8
1311	205	Manganese superoxide dismutase (EC 1.15.1.1)	3580	8
1312	222	Menaquinone via futalosine step 2	3581	8
1313	553	hypothetical protein TTHA0555	3582	8
1314	157	Small multidrug export protein	3583	8
1315	119	LSU ribosomal protein L20p	3584	8
1316	65	LSU ribosomal protein L35p	3585	8
1317	158	Translation initiation factor 3	3586	8
1318	254	Enoyl-CoA hydratase (EC 4.2.1.17)	3587	8
1319	550	Glutaminyl-tRNA synthetase (EC 6.1.1.18)	3588	8
1320	143	FIG01181663: hypothetical protein	3589	8
1321	104	FIG01181526: hypothetical protein	3590	8
1322	236	RNA binding methyltransferase FtsJ like	3591	8
1323	330	Aspartate-semialdehyde dehydrogenase (EC 1.2.1.11)	3592	8
1324	83	FIG01181218: hypothetical protein	3593	8
1325	64	hypothetical protein		8
1326	507	Glycyl-tRNA synthetase (EC 6.1.1.14)	3594	8
1327	735	ATP-dependent Clp protease ATP-binding subunit ClpA	3595	8
1328	424	DNA repair protein RadA	3596	8
1329	333	Membrane-associated protein containing RNA-binding TRAM domain and ribonuclease PIN-domain,	3597	8
1330	185	hypothetical protein	3598	8
1331	379	Succinyl-CoA ligase [ADP-forming] beta chain (EC 6.2.1.5)	3599	8
1332	289	Succinyl-CoA ligase [ADP-forming] alpha chain (EC 6.2.1.5)	3600	8
1333	501	4-alpha-glucanotransferase (amylomaltase) (EC 2.4.1.25)	3601	8
1334	291	3-hydroxybutyryl-CoA dehydrogenase (EC 1.1.1.157)	3602	8
1335	286	Permease of the drug/metabolite transporter (DMT) superfamily	3603	8
1336	407	Zinc protease (EC 3.4.99.-)	3604	8
1337	392	Zinc protease(EC:3.4.99.-)	3605	8
1338	768	ATP-dependent DNA helicase RecG (EC 3.6.1.-)	3606	8
1339	251	FIG01181235: hypothetical protein	3607	8
1340	330	Putative esterase	3608	8
1341	806	CRISPR-associated protein, Csm1 family	3609	8
1342	135	CRISPR-associated protein, Csm2 family	3610	8
1343	244	CRISPR-associated RAMP Csm3	3611	8
1344	289	CRISPR-associated RAMP protein, Csm4 family	3612	8
1345	376	CRISPR-associated protein, Csm5 family	3613	8
1346	95	hypothetical protein	3614	8
1347	317	FIG01181042: hypothetical protein	3615	8
1348	238	hypothetical protein	3616	8
1349	88	hypothetical protein		8
1350	462	CRISPR-associated protein Cas02710	3617	8
1351	248	CRISPR repeat RNA endoribonuclease Cas6	3618	8
1352	963	Exonuclease SbcC	3619	8
1353	372	Exonuclease SbcD	3620	8
1354	337	Putative membrane protein	3621	8
1355	138	Peroxide stress regulator; Ferric uptake regulation protein; Fe2+/Zn2+ uptake regulation proteins	3622	8
1356	133	FIG01181280: hypothetical protein	3623	8
1357	882	FIG01181019: hypothetical protein	3624	8
1358	205	Competence protein F homolog, phosphoribosyltransferase domain; protein YhgH required for utiliza	3626	8
1359	331	Methylthioribose-1-phosphate isomerase (EC 5.3.1.23)	3627	8
1360	374	FIG01181495: hypothetical protein	3628	8
1361	189	hypothetical protein	3629	8

1362	344	L-threonine 3-dehydrogenase (EC 1.1.1.103)	4080	4
1363	2665	FIG01181221: hypothetical protein	4081	4
1364	89	Acylphosphate phosphohydrolase (EC 3.6.1.7), putative	4082	4
1365	549	GTP-binding protein HflX	4083	4
1366	212	hypothetical protein	4084	4
1367	150	Cell division initiation protein DivIVA	4085	4
1368	212	Hypothetical protein YggS, proline synthase co-transcribed bacterial homolog PROSC	4086	4
1369	277	Purine nucleoside phosphorylase (EC 2.4.2.1)	4087	4
1370	826	Outer membrane protein	4088	4
1371	103	YlxP-like protein	4089	4
1372	88	hypothetical protein	4090	4
1373	87	Putative ORF-2	4091	4
1374	404	3-ketoacyl-CoA thiolase (EC 2.3.1.16) @ Acetyl-CoA acetyltransferase (EC 2.3.1.9)	4092	4
1375	504	hypothetical protein	4093	4
1376	148	Pilin, type IV, putative	4094	4
1377	146	hypothetical protein	4095	4
1378	203	hypothetical protein	4096	4
1379	135	Pilin, type IV, putative	4097	4
1380	55	FIG01181482: hypothetical protein	4098	4
1381	64	hypothetical protein	4099	4
1382	543	Mobile element protein	4100	4
1383	181	FIG01181482: hypothetical protein	4101	4
1384	67	FIG01181605: hypothetical protein	4102	4
1385	186	FIG01180887: hypothetical protein	4103	4
1386	226	Arginine/ornithine antiporter ArcD	4104	4
1387	215	FIG01181234: hypothetical protein	4105	4
1388	338	Aminoacyl-tRNA synthetase class Ib; tryptophanyl-tRNA synthetase	4106	4
1389	471	3-isopropylmalate dehydratase large subunit (EC 4.2.1.33)	4107	4
1390	202	3-isopropylmalate dehydratase small subunit (EC 4.2.1.33)	4108	4
1391	346	3-isopropylmalate dehydrogenase (EC 1.1.1.85)	4109	4
1392	178	COG1355, Predicted dioxygenase	4110	4
1393	179	COG1355, Predicted dioxygenase	4111	4
1394	245	Putative dehydrogenase	4112	4
1395	551	Dihydroxy-acid dehydratase (EC 4.2.1.9)	4113	4
1396	127	Cytochrome c-552 precursor	4114	4
1397	346	ABC transporter, periplasmic spermidine putrescine-binding protein PotD (TC 3.A.1.11.1)	4115	4
1398	260	Spermidine Putrescine ABC transporter permease component PotC (TC_3.A.1.11.1)	4116	4
1399	287	Spermidine Putrescine ABC transporter permease component PotB (TC 3.A.1.11.1)	4117	4
1400	356	Putrescine transport ATP-binding protein PotA (TC 3.A.1.11.1)	4118	4
1401	360	Rod shape-determining protein RodA	4119	4
1402	74	Cell division topological specificity factor MinE	4120	4
1403	268	Septum site-determining protein MinD	4121	4
1404	102	Transcriptional regulator, HxlR family	4122	4
1405	44	hypothetical protein	4123	4
1406	515	Methylmalonyl-CoA mutase (EC 5.4.99.2)	4124	4
1407	127	Protein kinase-like protein	4125	4
1408	649	Acetyl-coenzyme A synthetase (EC 6.2.1.1)	4126	4
1409	845	Acetyl-coenzyme A synthetase (EC 6.2.1.1)	4127	4
1410	628	Acetyl-coenzyme A synthetase (EC 6.2.1.1)	4128	4
1411	86	FIG152265: Sodium:solute symporter associated protein	4129	4
1412	557	Acetate permease ActP (cation/acetate symporter)	4130	4
1413	120	FIG01180892: hypothetical protein	4131	4
1414	581	Predicted signal-transduction protein containing cAMP-binding and CBS domains	4132	4
1415	59	hypothetical protein	4133	4
1416	831	Serine phosphatase RsbU, regulator of sigma subunit	4134	4
1417	119	Response regulator receiver protein in cluster with DNA polymerase III epsilon subunit	4135	4
1418	628	DNA polymerase III epsilon subunit (EC 2.7.7.7)	4136	4
1419	418	Phosphoribosylamine--glycine ligase (EC 6.3.4.13)	4137	4
1420	297	Phosphoribosylglycinamide formyltransferase (EC 2.1.2.2)	4138	4
1421	299	Metallo-beta-lactamase protein	4139	4
1422	265	5-carboxymethyl-2-hydroxymuconate delta-isomerase (EC 5.3.3.10)	4140	4
1423	178	hypothetical membrane spanning protein	4141	4
1424	360	Transcriptional regulator	4142	4
1425	148	hypothetical membrane spanning protein	4143	4
1426	154	FIG01181288: hypothetical protein	4144	4
1427	373	Gene SCO4494, often clustered with other genes in menaquinone via futalosine pathway	4145	4
1428	271	Menaquinone via futalosine step 1	4146	4
1429	179	YceI like family protein	4147	4

1430	131	hypothetical protein	4148	4
1431	FLOn	Glyoxalase family protein	4149	4
1432	345	2-keto-3-deoxy-D-arabino-heptulosonate-7-phosphate synthase I beta (EC 2.5.1.54)	4150	4
1433	360	Prephenate and/or arogenate dehydrogenase (unknown specificity) (EC 1.3.1.12)(EC 1.3.1.43)	4151	4
1434	319	FIG01180970: hypothetical protein	4153	4
1435	448	Glycolate dehydrogenase (EC 1.1.99.14), subunit GlcD	4154	4
1436	162	FIG01181053: hypothetical protein	4155	4
1437	160	Fructokinase (EC 2.7.1.4)	4156	4
1438	121	hypothetical protein	4157	4
1439	163	FIG00788204: hypothetical protein	4158	4
1440	407	Type IV fimbrial assembly protein PilC	4159	4
1441	197	TsaC protein (YrdC domain) required for threonylcarbamoyladenosine t(6)A37 modification in tRNA	4160	4
1442	174	Chromosome segregation and condensation protein ScpB	4161	4
1443	358	FIG01181139: hypothetical protein	4162	4
1444	209	N-acetylglutamate synthase (EC 2.3.1.1)	4163	4
1445	386	Glutaryl-CoA dehydrogenase (EC 1.3.99.7)	4164	4
1446	300	DNA polymerase III delta subunit (EC 2.7.7.7)	4165	4
1447	465	TldD protein, part of TldE/TldD proteolytic complex	4166	4
1448	437	TldE protein, part of TldE/TldD proteolytic complex	4167	4
1449	313	Glyoxylate reductase (EC 1.1.1.79) / Glyoxylate reductase (EC 1.1.1.26) / Hydroxypyruvate reductase	4168	4
1450	247	Putative membrane protein, putative	4169	4
1451	44	hypothetical protein	4170	4
1452	182	Uracil phosphoribosyltransferase (EC 2.4.2.9) / Pyrimidine operon regulatory protein PyrR	4171	4
1453	302	Aspartate carbamoyltransferase (EC 2.1.3.2)	4172	4
1454	426	Dihydroorotase (EC 3.5.2.3)	4173	4
1455	159	hypothetical conserved protein	4174	4
1456	346	Dihydroorotate dehydrogenase (EC 1.3.3.1)	4175	4
1457	143	FIG01181591: hypothetical protein	4176	4
1458	795	ATP-dependent protease La (EC 3.4.21.53) Type I	4177	4
1459	187	Molybdopterin-guanine dinucleotide biosynthesis protein MobA	4178	4
1460	293	FAD dependent oxidoreductase precursor	4179	4
1461	111	hypothetical protein	4180	4
1462	441	tRNA-i(6)A37 methylthiotransferase	4181	4
1463	94	hypothetical protein	4182	4
1464	371	FAD dependent oxidoreductase	4183	4
1465	497	Isocitrate dehydrogenase [NADP] (EC 1.1.1.42) @ Homoisocitrate dehydrogenase (EC 1.1.1.87)	4184	4
1466	599	Exoribonuclease II (EC 3.1.13.1)	4185	4
1467	237	hypothetical protein	4186	4
1468	571	Oligoendopeptidase F (EC 3.4.24.-)	4187	4
1469	349	tRNA pseudouridine 13 synthase (EC 4.2.1.-)	4188	4
1470	211	FIG01181629: hypothetical protein	4189	4
1471	135	comA operon protein 2	4190	4
1472	321	Membrane dipeptidase (EC 3.4.13.19)	4191	4
1473	596	Excinuclease ABC subunit C	4192	4
1474	346	BNR repeat domain protein	4193	4
1475	593	Arginyl-tRNA synthetase (EC 6.1.1.19)	4194	4
1476	350	probable serine protease	4195	4
1477	201	Deoxyadenosine kinase (EC 2.7.1.76) / Deoxyguanosine kinase (EC 2.7.1.113)	4196	4
1478	203	Deoxyadenosine kinase (EC 2.7.1.76) / Deoxyguanosine kinase (EC 2.7.1.113)	4197	4
1479	487	UDP-N-acetylmuramoylalanyl-D-glutamate--L-ornithine ligase	4198	4
1480	442	tRNA:m(5)U-54 MTase gid	4199	4
1481	177	COG1355, Predicted dioxygenase	4200	4
1482	253	Imidazole glycerol phosphate synthase cyclase subunit (EC 4.1.3.-)	4201	4
1483	210	Phosphoribosyl-AMP cyclohydrolase (EC 3.5.4.19) / Phosphoribosyl-ATP pyrophosphatase (EC 3.6.1.3	4202	4
1484	55	hypothetical protein	4203	4
1485	323	Fructose-1,6-bisphosphatase, GlpX type (EC 3.1.3.11)	4204	4
1486	50	hypothetical protein	4205	4
1487	225	FIG01181646: hypothetical protein	4206	4
1488	621	Tungsten-containing aldehyde ferredoxin oxidoreductase (EC 1.2.7.5)	4207	4
1489	182	TsaB protein, required for threonylcarbamoyladenosine (t(6)A) formation in tRNA	4208	4
1490	214	hypothetical conserved protein	4209	4
1491	329	AttH protein	4210	4
1492	251	Putative deoxyribonuclease YcfH	4211	4
1493	430	Cardiolipin synthetase (EC 2.7.8.-)	4212	4
1494	188	hypothetical protein	4213	4
1495	100	FIG01181225: hypothetical protein	4214	4
1496	423	Aspartyl-tRNA synthetase (EC 6.1.1.12) @ Aspartyl-tRNA(Asn) synthetase (EC 6.1.1.23)	4215	4
1497	233	Succinate dehydrogenase iron-sulfur protein (EC 1.3.99.1)	4216	4

1498	570	Succinate dehydrogenase flavoprotein subunit (EC 1.3.99.1)	4217	4
1499	132	Succinate dehydrogenase hydrophobic membrane anchor protein	4218	4
1500	122	Succinate dehydrogenase cytochrome b-556 subunit	4219	4
1501	145	S-adenosylmethionine decarboxylase proenzyme (EC 4.1.1.50), prokaryotic class 1B	4220	4
1502	382	FIG00673898: hypothetical protein	4221	4
1503	308	ABC transporter ATP-binding protein	4222	4
1504	319	ABC-type transport, permease protein	4223	4
1505	126	Cupin	4224	4
1506	209	Putative phosphoribosyl transferase	4225	4
1507	558	Long-chain-fatty-acid--CoA ligase (EC 6.2.1.3)	4226	4
1508	373	Cysteine desulfurase (EC 2.8.1.7)	4227	4
1509	149	Iron-sulfur cluster regulator IscR	4228	4
1510	269	Zinc ABC transporter, inner membrane permease protein ZnuB	4229	4
1511	247	Manganese transport system ATP-binding protein MntA	4230	4
1512	81	Acyl carrier protein	4231	4
1513	409	3-oxoacyl-[acyl-carrier-protein] synthase, KASII (EC 2.3.1.179)	4232	4
1514	376	Deoxyguanosinetriphosphate triphosphohydrolase (EC 3.1.5.1)	4234	4
1515	397	Transcriptional regulator, GntR family domain / Aspartate aminotransferase (EC 2.6.1.1)	4235	4
1516	379	Phenylacetate-coenzyme A ligase (EC 6.2.1.30)	4236	4
1517	307	Ribokinase (EC 2.7.1.15)	4237	4
1518	199	Imidazole glycerol phosphate synthase amidotransferase subunit (EC 2.4.2.-)	4238	4
1519	195	Imidazoleglycerol-phosphate dehydratase (EC 4.2.1.19)	4239	4
1520	348	Histidinol-phosphate aminotransferase (EC 2.6.1.9)	4240	4
1521	40	hypothetical protein		4
1522	518	Transposase, IS605 family, OrfB	4241	4
1523	148	DNA polymerase beta domain protein region	4242	4
1524	314	Thiamine-monophosphate kinase (EC 2.7.4.16)	4243	4
1525	239	FIG01181362: hypothetical protein	4244	4
1526	235	N-acetylmannosaminyltransferase (EC 2.4.1.187)	4245	4
1527	648	FIG00788636: hypothetical protein	4246	4
1528	828	hypothetical protein	4247	4
1529	531	Type III restriction-modification system methylation subunit (EC 2.1.1.72)	4248	4
1530	71	hypothetical protein	4249	4
1531	361	ADP-ribose 1"-phosphate phosphatase related protein	4250	4
1532	113	FIG01181472: hypothetical protein	4251	4
1533	213	Putative DNA-binding protein Erf	4252	4
1534	248	FIG01181710: hypothetical protein	4253	4
1535	273	SSU rRNA (adenine(1518)-N(6)/adenine(1519)-N(6))-dimethyltransferase (EC 2.1.1.182)	4254	4
1536	113	NADH ubiquinone oxidoreductase chain A (EC 1.6.5.3)	4255	4
1537	182	NADH-ubiquinone oxidoreductase chain B (EC 1.6.5.3)	4256	4
1538	207	NADH-ubiquinone oxidoreductase chain C (EC 1.6.5.3)	4257	4
1539	407	NADH-ubiquinone oxidoreductase chain D (EC 1.6.5.3)	4258	4
1540	182	NADH-ubiquinone oxidoreductase chain E (EC 1.6.5.3)	4259	4
1541	438	NADH-ubiquinone oxidoreductase chain F (EC 1.6.5.3)	4260	4
1542	784	NADH-ubiquinone oxidoreductase chain G (EC 1.6.5.3)	4261	4
1543	364	NADH-ubiquinone oxidoreductase chain H (EC 1.6.5.3)	4262	4
1544	183	NADH-ubiquinone oxidoreductase chain I (EC 1.6.5.3)	4263	4
1545	178	NADH-ubiquinone oxidoreductase chain J (EC 1.6.5.3)	4264	4
1546	96	NADH-ubiquinone oxidoreductase chain K (EC 1.6.5.3)	4265	4
1547	607	NADH-ubiquinone oxidoreductase chain L (EC 1.6.5.3)	4266	4
1548	470	NADH-ubiquinone oxidoreductase chain M (EC 1.6.5.3)	4267	4
1549	427	NADH-ubiquinone oxidoreductase chain N (EC 1.6.5.3)	4268	4
1550	543	Mobile element protein	4270	4
1551	910	Aconitate hydratase (EC 4.2.1.3)	4271	4
1552	222	V-type ATP synthase subunit D (EC 3.6.3.14)	4272	4
1553	479	V-type ATP synthase subunit B (EC 3.6.3.14)	4273	4
1554	579	V-type ATP synthase subunit A (EC 3.6.3.14)	4274	4
1555	107	V-type ATP synthase subunit F (EC 3.6.3.14)	4276	4
1556	324	V-type ATP synthase subunit C (EC 3.6.3.14)	4277	4
1557	189	V-type ATP synthase subunit E (EC 3.6.3.14)	4278	4
1558	100	V-type ATP synthase subunit K (EC 3.6.3.14)	4279	4
1559	650	V-type ATP synthase subunit I (EC 3.6.3.14)	4280	4
1560	105	V-type ATP synthase subunit G (EC 3.6.3.14)	4281	4
1561	289	Mobile element protein	4282	4
1562	180	hypothetical protein	4283	4
1563	145	hypothetical protein	4284	4
1564	507	hypothetical protein	4286	2
1565	101	hypothetical protein	4290	2

1566	445	DehaBAV1-1041 hypothetical protein	4291	2
1567	382	LSU m5C1962 methyltransferase RlmI	4292	2
1568	193	hypothetical protein	4293	2
1569	70	FIG01181583: hypothetical protein	4294	2
1570	122	FIG01181233: hypothetical protein	4295	2
1571	225	Metal-dependent hydrolase (EC 3.-.-.-)	4296	2
1572	275	Serine/threonine-protein kinase Pkn2 (EC 2.7.11.1)	4297	2
1573	631	Acetoacetyl-CoA synthetase (EC 6.2.1.16)	4298	2
1574	794	hypothetical protein	4299	2
1575	495	Virulence factor MviN	4300	2
1576	353	FIG01181048: hypothetical protein	4301	2
1577	384	Cell envelope-associated transcriptional attenuator LytR-CpsA-Psr, subfamily M (as in PMID1909955[illegible]	4202	2
1578	471	FIG01181194: hypothetical protein	4303	2
1579	266	Vitamin B12 ABC transporter, B12-binding component BtuF	4304	2
1580	413	Histidinol dehydrogenase (EC 1.1.1.23)	4305	2
1581	199	FIG01180900: hypothetical protein	4306	2
1582	354	FIG01181491: hypothetical protein	4307	2
1583	123	FIG01180938: hypothetical protein	4308	2
1584	205	Uracil-DNA glycosylase, family 4	4309	2
1585	290	Molybdenum ABC transporter, periplasmic molybdenum-binding protein ModA (TC 3.A.1.8.1)	4310	2
1586	225	Molybdenum transport system permease protein ModB (TC 3.A.1.8.1)	4311	2
1587	333	Molybdenum transport ATP-binding protein ModC (TC 3.A.1.8.1)	4312	2
1588	107	hypothetical protein	4314	2
1589	419	Histidyl-tRNA synthetase (EC 6.1.1.21)	4315	2
1590	582	Aspartyl-tRNA synthetase (EC 6.1.1.12)	4316	2
1591	292	Phenazine biosynthesis protein PhzF	4317	2
1592	276	Hydrolase (HAD superfamily)	4318	2
1593	439	Asparaginyl-tRNA synthetase (EC 6.1.1.22)	4319	2
1594	192	Pyridoxine biosynthesis glutamine amidotransferase, glutaminase subunit (EC 2.4.2.-)	4320	2
1595	287	probable UV endonuclease	4321	2
1596	172	FIG01181439: hypothetical protein	4322	2
1597	178	FIG01181323: hypothetical protein	4323	2
1598	208	Sepiapterin reductase (EC 1.1.1.153)	4324	2
1599	261	Proteorhodopsin	4325	2
1600	256	hypothetical protein	4326	2
1601	507	Nucleoside-diphosphate-sugar epimerase	4327	2
1602	468	Carotenoid cis-trans isomerase (EC 5.2.-.-)	4328	2
1603	422	hypothetical protein	4329	2
1604	238	Putative 3-demethylubiquinone-9 3-methyltransferase	4330	2
1605	222	FIG01181215: hypothetical protein	4331	2
1606	414	Na+/H+ antiporter NhaA type	4332	2
1607	673	Cadmium-transporting ATPase (EC 3.6.3.3)	4333	2
1608	124	Cadmium efflux system accessory protein	4334	2
1609	294	Pyridoxine biosynthesis glutamine amidotransferase, synthase subunit (EC 2.4.2.-)	4335	2
1610	395	Cell wall endopeptidase, family M23/M37	4336	2
1611	427	Transcription termination factor Rho	4337	2
1612	222	Transaldolase (EC 2.2.1.2)	4338	2
1613	1048	Isoleucyl-tRNA synthetase (EC 6.1.1.5)	4339	2
1614	185	hypothetical protein	4340	2
1615	346	Aminopeptidase YpdF (MP-, MA-, MS-, AP-, NP- specific)	4341	2
1616	164	Rare lipoprotein A precursor	4342	2
1617	277	Endonuclease IV (EC 3.1.21.2)	4343	2
1618	49	hypothetical protein	4344	2
1619	220	FIG004556: membrane metalloprotease	4345	2
1620	202	Membrane metalloprotease	4346	2
1621	834	tRNA nucleotidyltransferase, A-adding (EC 2.7.7.25)	4347	2
1622	273	Oxidoreductase family protein	4348	2
1623	97	hypothetical protein	4349	2
1624	57	hypothetical protein		2
1625	455	Mg/Co/Ni transporter MgtE / CBS domain	4350	2
1626	214	FIG01181701: hypothetical protein	4351	2
1627	50	DNA repair protein RecN	4352	2
1628	238	Adenine-specific methyltransferase (EC 2.1.1.72)	4353	2
1629	82	hypothetical protein	4354	2
1630	46	hypothetical protein	4355	2
1631	306	Transposase	4356	2
1632	90	Transposase	4357	2
1633	264	SSU ribosomal protein S2p (SAe)	4358	2

1634	194	Translation elongation factor Ts	4359	2
1635	251	Uridine monophosphate kinase (EC 2.7.4.22)	4360	2
1636	186	Ribosome recycling factor	4361	2
1637	275	Phosphatidate cytidylyltransferase (EC 2.7.7.41)	4362	2
1638	368	1-deoxy-D-xylulose 5-phosphate reductoisomerase (EC 1.1.1.267)	4363	2
1639	337	Membrane-associated zinc metalloprotease	4364	2
1640	209	Glycosyltransferase involved in cell wall biogenesis	4365	2
1641	175	FIG01181017: hypothetical protein	4366	2
1642	262	Pyrroline-5-carboxylate reductase (EC 1.5.1.2)	4367	2
1643	399	Ribosomal RNA small subunit methyltransferase B (EC 2.1.1.-)	4368	2
1644	91	SpoVS-related protein, type 4	4369	2
1645	146	hypothetical conserved protein	4370	2
1646	143	Regulatory protein RecX	4371	2
1647	228	Phosphohydrolase	4372	2
1648	183	CAAX amino terminal protease family protein	4373	2
1649	671	Serine protein kinase (PrkA protein), P-loop containing	4374	2
1650	348	Glycosyltransferase	4375	2
1651	458	FIG004684: SpoVR-like protein	4376	2
1652	264	Thioredoxin	4377	2
1653	125	Rhodanese-like domain protein	4378	2
1654	230	Glutaredoxin-like protein	4379	2
1655	42	hypothetical protein	4380	2
1656	479	Metallo-beta-lactamase family protein	4380	2
1657	220	putative transferase/hydrolase	4381	2
1658	248	FIG01181560: hypothetical protein	4382	2
1659	283	FIG01180920: hypothetical protein	4383	2
1660	43	hypothetical protein	4384	2
1661	388	Uroporphyrinogen III decarboxylase (EC 4.1.1.37)	4385	2
1662	320	Ferrochelatase, protoheme ferro-lyase (EC 4.99.1.1)	4386	2
1663	452	Protoporphyrinogen IX oxidase, aerobic, HemY (EC 1.3.3.4)	4387	2
1664	748	FIG01180894: hypothetical protein	4388	2
1665	119	hypothetical protein	4389	2
1666	858	Diguanylate cyclase/phosphodiesterase (GGDEF & EAL domains) with PAS/PAC sensor(s)	4390	2
1667	979	HD-hydrolase domain	4391	2
1668	336	Periplasmic serine protease, HtrA/DegQ/DegS family	4392	2
1669	507	Alkaline serine exoprotease A precursor (EC 3.4.21.-)	4393	2
1670	329	Putative hydrolase	4394	2
1671	354	FIG01181251: hypothetical protein	4395	2
1672	362	Sorbitol dehydrogenase (EC 1.1.1.14)	4396	2
1673	162	Bacterioferritin comigratory protein	4397	2
1674	285	Cell division protein FtsX	4398	2
1675	427	FIG01181574: hypothetical protein	4399	2
1676	187	Ribosomal subunit interface protein	4400	2
1677	243	Cell division transporter, ATP-binding protein FtsE (TC 3.A.5.1.1)	4401	2
1678	444	Carboxyl-terminal protease (EC 3.4.21.102)	4402	2
1679	290	hypothetical protein	4403	2
1680	620	Methionyl-tRNA synthetase (EC 6.1.1.10)	4404	2
1681	118	hypothetical protein	4405	2
1682	73	hypothetical protein	4406	2
1683	107	hypothetical protein	4407	2
1684	73	hypothetical protein	4408	2
1685	228	Ribose 5-phosphate isomerase A (EC 5.3.1.6)	4409	2
1686	155	Thiol peroxidase, Bcp-type (EC 1.11.1.15)	4410	2
1687	162	Probable transmembrane protein	4411	2
1688	379	Menaquinone via futalosine step 3	4412	2
1689	260	Tryptophan synthase alpha chain (EC 4.2.1.20)	4413	2
1690	406	Tryptophan synthase beta chain (EC 4.2.1.20)	4414	2
1691	268	Thymidylate synthase ThyX (EC 2.1.1.-)	4415	2
1692	675	DNA ligase (EC 6.5.1.2)	4416	2
1693	214	FIG01181701: hypothetical protein	4417	2
1694	201	Periplasmic thiol:disulfide interchange protein DsbA	4418	2
1695	315	Spermidine synthase (EC 2.5.1.16)	4419	2
1696	130	S-adenosylmethionine decarboxylase proenzyme (EC 4.1.1.50), prokaryotic class 1B	4420	2
1697	863	Cell division protein FtsK	4421	2
1698	164	Putative cationic outer membrane protein OmpH	4422	2
1699	147	FIG01181259: hypothetical protein	4423	2
1700	211	CBS domain protein	4424	2
1701	274	Prephenate dehydratase (EC 4.2.1.51)	4425	2

1702	250	Fructokinase (EC 2.7.1.4)	4426	2
1703	341	tRNA-dependent lipid II--glycine ligase @ tRNA-dependent lipid II-Gly--glycine ligase	4427	2
1704	189	FIG00788758: hypothetical protein	4428	2
1705	286	FIG01181039: hypothetical protein	4429	2
1706	306	hypothetical conserved protein	4430	2
1707	497	Sugar transport ATP-binding protein	4431	2
1708	375	Membrane lipoprotein	4432	2
1709	60	hypothetical protein	4433	2
1710	512	Glutathione-regulated potassium-efflux system protein KefC	4434	2
1711	245	FIG000859: hypothetical protein YebC	4435	2
1712	998	Preprotein translocase subunit SecA (TC 3.A.5.1.1)	4436	2
1713	323	TsaD/Kae1/Qri7 protein, required for threonylcarbamoyladenosine t(6)A37 formation in tRNA		2
1714	437	FIG01180861: hypothetical protein	4437	2
1715	142	Fe-S binding reductase, putative	4438	2
1716	70	FIG01181062: hypothetical protein	4439	2
1717	734	Probable ATP-dependent helicase lhr (EC 3.6.1.-)	4440	2
1718	137	FIG01180858: hypothetical protein	4441	2
1719	846	Adenylate cyclase (EC 4.6.1.1)	4442	2
1720	578	DNA primase (EC 2.7.7.-)	4443	2
1721	328	Malate dehydrogenase (EC 1.1.1.37)	4444	2
1722	406	Aspartokinase (EC 2.7.2.4)	4445	2
1723	27	hypothetical protein	4446	2
1724	375	Ribosomal RNA small subunit methyltransferase C (EC 2.1.1.52)	4447	2
1725	440	RNA polymerase sigma factor RpoD	4448	2
1726	165	FIG01181012: hypothetical protein	4449	2
1727	458	Exoenzymes regulatory protein AepA precursor	4450	2
1728	163	ADP-ribose pyrophosphatase (EC 3.6.1.13)	4451	2
1729	295	Riboflavin kinase (EC 2.7.1.26) / FMN adenylyltransferase (EC 2.7.7.2)	4452	2
1730	475	Glycine dehydrogenase [decarboxylating] (glycine cleavage system P2 protein) (EC 1.4.4.2)	4453	2
1731	438	Glycine dehydrogenase [decarboxylating] (glycine cleavage system P1 protein) (EC 1.4.4.2)	4454	2
1732	128	Glycine cleavage system H protein	4455	2
1733	350	Aminomethyltransferase (glycine cleavage system T protein) (EC 2.1.2.10)	4456	2
1734	575	Bipolar DNA helicase HerA	4457	2
1735	295	Single-stranded exonuclease associated with Rad50/Mre11 complex	4459	2
1736	577	NAD-dependent malic enzyme (EC 1.1.1.38)	4460	2
1737	117	PaaD-like protein (DUF59) involved in Fe-S cluster assembly	4461	2
1738	232	TerC-like integral membrane protein	4462	2
1739	101	hypothetical protein	4463	2
1740	319	Putative sodium-dependent bicarbonate transporter	4464	2
1741	157	Phosphoribosylaminoimidazole carboxylase catalytic subunit (EC 4.1.1.21)	4465	2
1742	369	Phosphoribosylaminoimidazole carboxylase ATPase subunit (EC 4.1.1.21)	4466	2
1743	385	hypothetical protein	4467	2
1744	308	Ribosomal large subunit pseudouridine synthase D (EC 4.2.1.70)	4468	2
1745	261	3-methyl-2-oxobutanoate hydroxymethyltransferase (EC 2.1.2.11)	4469	2
1746	324	Holliday junction DNA helicase RuvB	4470	2
1747	346	FIG01181164: hypothetical protein	4471	2
1748	452	FIG00789878: hypothetical protein	4472	2
1749	616	Cell division protein FtsH (EC 3.4.24.-)	4473	2
1750	511	Aerobic glycerol-3-phosphate dehydrogenase (EC 1.1.5.3)	4474	2
1751	496	Glycerol kinase (EC 2.7.1.30)	4475	2
1752	349	Galactose-1-phosphate uridylyltransferase (EC 2.7.7.10)	4476	2
1753	55	hypothetical protein	4477	2
1754	63	Beta-mannosidase Man2	4478	2
1755	322	D-alanine--D-alanine ligase (EC 6.3.2.4)	4479	2
1756	185	Peptidyl-tRNA hydrolase (EC 3.1.1.29)	4480	2
1757	206	LSU ribosomal protein L25p	4481	2
1758	308	Ribose-phosphate pyrophosphokinase (EC 2.7.6.1)	4482	2
1759	226	5'-methylthioadenosine nucleosidase (EC 3.2.2.16) @ S-adenosylhomocysteine nucleosidase (EC 3.2.:	4483	2
1760	153	S-ribosylhomocysteine lyase (EC 4.4.1.21) / Autoinducer-2 production protein LuxS	4484	2
1761	504	GMP synthase [glutamine-hydrolyzing] (EC 6.3.5.2)	4485	2
1762	190	FIG01181189: hypothetical protein	4486	2
1763	96	FIG01181117: hypothetical protein	4487	2
1764	126	Glyoxalase family protein	4488	2
1765	499	hypothetical conserved protein	4489	2
1766	51	hypothetical protein		2
1767	459	Arylsulfatase regulator	4490	2
1768	253	hypothetical conserved protein	4491	2
1769	301	ABC transporter ATP-binding protein	4492	2

1770	162	Ribonuclease HI (EC 3.1.26.4)	4493	2
1771	551	Methylcrotonyl-CoA carboxylase carboxyl transferase subunit (EC 6.4.1.4)	4494	2
1772	286	Hydroxymethylglutaryl-CoA lyase (EC 4.1.3.4)	4495	2
1773	132	FIG01181161: hypothetical protein	4496	2
1774	163	FIG01181458: hypothetical protein	4497	2
1775	59	hypothetical protein	4498	2
1776	202	hypothetical protein	4499	2
1777	118	FIG039061: hypothetical protein related to heme utilization	4500	2
1778	510	tRNA(ile)-lysidine synthetase	4501	2
1779	133	hypothetical protein	4502	2
1780	459	Glycolate dehydrogenase (EC 1.1.99.14), subunit GlcD	4503	2
1781	352	Glycolate dehydrogenase (EC 1.1.99.14), FAD-binding subunit GlcE	4504	2
1782	419	Glycolate dehydrogenase (EC 1.1.99.14), iron-sulfur subunit GlcF	4505	2
1783	65	hypothetical protein		2
1784	410	hypothetical protein	4506	2
1785	87	FIG01180936: hypothetical protein	4507	2
1786	131	FIG01181087: hypothetical protein	4508	2
1787	407	D-glycerate 2-kinase (EC 2.7.1.-)	4509	2
1788	369	Hypothetical radical SAM family enzyme in heat shock gene cluster, similarity with CPO of BS HemN-t	4510	2
1789	130	NADH-quinone oxidoreductase subunit 15 (EC 1.6.99.5)	4511	2
1790	476	Phosphoglycerate kinase (EC 2.7.2.3)	4512	2
1791	113	FIG01181465: hypothetical protein	4513	2
1792	99	FIG01181046: hypothetical protein	4514	2
1793	104	FIG01180870: hypothetical protein	4515	2
1794	352	Threonine synthase (EC 4.2.3.1)	4516	2
1795	188	hypothetical cytosolic protein	4517	2
1796	333	Homoserine dehydrogenase (EC 1.1.1.3)	4518	2
1797	409	4Fe-4S ferredoxin iron-sulfur binding domain protein	4519	2
1798	235	Diadenosine tetraphosphatase and related serine/threonine protein phosphatases	4520	2
1799	444	Apolipoprotein N-acyltransferase	4521	2
1800	248	Transcriptional regulator, ArsR family	4522	2
1801	213	Methyltransferase TTHA0482	4523	2
1802	529	Maltodextrin glucosidase (EC 3.2.1.20)	4524	2
1803	75	hypothetical protein	4525	2
1804	322	tRNA(U54)-2-thioribothymidine synthetase	4526	2
1805	66	TtuB	4527	2
1806	375	NAD-independent protein deacetylase AcuC	4528	2
1807	209	Inosine-5'-monophosphate dehydrogenase (EC 1.1.1.205)	4529	2
1808	328	Oligopeptide transport ATP-binding protein OppF (TC 3.A.1.5.1)	4530	2
1809	340	Oligopeptide transport ATP-binding protein OppD (TC 3.A.1.5.1)	4531	2
1810	451	Oligopeptide transport system permease protein OppC (TC 3.A.1.5.1)	4532	2
1811	328	Oligopeptide transport system permease protein OppB (TC 3.A.1.5.1)	4533	2
1812	583	Oligopeptide ABC transporter, periplasmic oligopeptide-binding protein OppA (TC 3.A.1.5.1)	4534	2
1813	344	Alcohol dehydrogenase (EC 1.1.1.1)	4535	2
1814	336	Thioredoxin reductase (EC 1.8.1.9)	4536	2
1815	181	FIG00788058: hypothetical protein	4537	2
1816	339	FIG01181536: hypothetical protein	4538	2
1817	175	Probable cysteinyl-tRNA synthetase (EC 6.1.1.16)	4539	2
1818	64	Probable transmembrane protein	4540	2
1819	110	Protein secretion chaperonin CsaA	4541	2
1820	344	Glucosamine-6-phosphate deaminase [isomerizing], alternative (EC 3.5.99.6)	4542	2
1821	78	hypothetical protein	4543	2
1822	133	Toxin 1, PIN domain	4544	2
1823	953	Excinuclease ABC subunit A	4545	2
1824	198	Transcriptional regulator, TetR family	4546	2
1825	165	FIG01181188: hypothetical protein	4547	2
1826	570	Putative membrane protein	4548	2
1827	285	2-nitropropane dioxygenase (EC 1.13.11.32)	4549	2
1828	48	hypothetical protein		2
1829	468	Glycosyl transferase, group 2 family protein	4550	2
1830	383	hypothetical protein	4551	2
1831	120	Phosphate regulon transcriptional regulatory protein PhoB (SphR)	4552	2
1832	723	GAF sensor signal transduction histidine kinase	4553	2
1833	106	Mannose-6-phosphate isomerase (EC 5.3.1.8)	4554	2
1834	223	Ribulose-phosphate 3-epimerase (EC 5.1.3.1)	4555	2
1835	145	FIG01180924: hypothetical protein	4556	2
1836	653	Transketolase (EC 2.2.1.1)	4557	2
1837	74	hypothetical protein	4558	2

1838	514	DEAD-box ATP-dependent RNA helicase CshA (EC 3.6.4.13)	4559	2
1839	405	Molybdopterin biosynthesis protein MoeA	4560	2
1840	378	Permease	4561	2
1841	218	Endonuclease III (EC 4.2.99.18)	4562	2
1842	124	FIG01181276: hypothetical protein	4563	2
1843	577	Methionine ABC transporter ATP-binding protein	4564	2
1844	73	hypothetical protein	4565	2
1845	478	Prolyl-tRNA synthetase (EC 6.1.1.15), archaeal/eukaryal type	4566	2
1846	407	2,3-bisphosphoglycerate-independent phosphoglycerate mutase, archaeal type (EC 5.4.2.1)	4567	2
1847	184	FIG01181008: hypothetical protein	4568	2
1848	326	FIG146085: 3'-to-5' oligoribonuclease A, Bacillus type	4569	2
1849	378	CRISPR-associated protein TM1812	4570	2
1850	355	CRISPR-associated RAMP Cmr6	4571	2
1851	115	CRISPR-associated RAMP Cmr5	4572	2
1852	287	CRISPR-associated RAMP Cmr4	4573	2
1853	399	CRISPR-associated RAMP Cmr1	4574	2
1854	369	CRISPR-associated RAMP Cmr3	4575	2
1855	585	CRISPR-associated RAMP Cmr2	4576	2
1856	628	FIG01180907: hypothetical protein	4577	2
1857	46	hypothetical protein		2
1858	70	hypothetical protein	4578	2
1859	80	Mobile element protein	4579	2
1860	60	Mobile element protein		2
1861	80	dnaJ/dnaK assembly factor dafA	4580	2
1862	277	DnaJ-class molecular chaperone CbpA	4581	2
1863	180	Heat shock protein GrpE	4582	2
1864	625	Chaperone protein DnaK	4583	2
1865	625	Cell division protein FtsH (EC 3.4.24.-)		2
1866	98	DNA-binding protein HBsu	3067	16
1867	210	1-acyl-sn-glycerol-3-phosphate acyltransferase (EC 2.3.1.51)	3068	16
1868	227	Endonuclease V (EC 3.1.21.7)	3069	16
1869	291	hypothetical protein	3070	16
1870	269	Mannose-6-phosphate isomerase (EC 5.3.1.8)	3071	16
1871	430	5-Enolpyruvylshikimate-3-phosphate synthase (EC 2.5.1.19)	3072	16
1872	209	Cytidylate kinase (EC 2.7.4.25)	3073	16
1873	261	FIG01181563: hypothetical protein	3074	16
1874	122	Dihydroneopterin triphosphate pyrophosphohydrolase	3075	16
1875	230	3-oxoacyl-[acyl-carrier protein] reductase (EC 1.1.1.100)	3076	16
1876	144	FIG01181053: hypothetical protein	3077	16
1877	185	Fructokinase (EC 2.7.1.4)	3078	16
1878	70	Transposase, IS4	3079	16
1879	80	Mobile element protein	3080	16
1880	60	Mobile element protein		16
1881	855	ClpB protein	3081	16
1882	130	Heat-stable protein	3082	16
1883	395	FIG01181210: hypothetical protein	3083	16
1884	138	Small heat shock protein	3084	16
1885	229	Heat-stable protein	3085	16
1886	140	Thioredoxin	3086	16
1887	138	Small heat shock protein	3087	16
1888	149	FIG01181375: hypothetical protein	3088	16
1889	140	FIG01181310: hypothetical protein	3089	16
1890	158	FIG01181662: hypothetical protein	3090	16
1891	378	Kef-type K(+) transport systems, membrane components	3091	16
1892	279	Alr3825 protein	3092	16
1893	211	GlpG protein (membrane protein of glp regulon)	3093	16
1894	145	Inosine-5'-monophosphate dehydrogenase related protein IX	3094	16
1895	385	Na(+)/H(+) antiporter (TC 2.A.37.2.4)	3095	16
1896	535	FIG01180955: hypothetical protein	3096	16
1897	445	Replicative DNA helicase (EC 3.6.1.-)	3097	16
1898	183	Tlr1989 protein	3098	16
1899	1493	Glutamate synthase [NADPH] large chain (EC 1.4.1.13)	3099	16
1900	154	FIG01181408: hypothetical protein	3100	16
1901	77	hypothetical protein	3101	16
1902	495	Inosine-5'-monophosphate dehydrogenase (EC 1.1.1.205)	3102	16
1903	224	Phosphate regulon transcriptional regulatory protein PhoB (SphR)	3103	16
1904	297	Phosphate regulon sensor protein PhoR (SphS) (EC 2.7.13.3)	3104	16
1905	221	Phosphate transport system regulatory protein PhoU	3105	16

1906	69	hypothetical protein	3106	16
1907	379	Peptide chain release factor 2; programmed frameshift-containing	3107	16
1908	149	FIG01181661: hypothetical protein	3110	16
1909	80	Mobile element protein	3111	16
1910	120	Mobile element protein	3112	16
1911	175	hypothetical protein	3113	16
1912	323	Beta lactamase	3114	16
1913	264	Transposase	3115	16
1914	433	UDP-N-acetylglucosamine 1-carboxyvinyltransferase (EC 2.5.1.7)	3116	16
1915	72	FIG00580021: hypothetical protein	3117	16
1916	606	Serine/threonine protein kinase	3118	16
1917	125	Bona fide RidA/YjgF/TdcF/RutC subgroup	3119	16
1918	314	Putative periplasmic protein kinase ArgK and related GTPases of G3E family	3120	16
1919	145	MutT/nudix family protein	3121	16
1920	159	Ribonucleotide reductase transcriptional regulator NrdR	3002	
1921	236	Sorbitol-6-phosphate 2-dehydrogenase (EC 1.1.1.140)	3003	
1922	160	ATPase associated with chromosome architecture/replication	3004	
1923	143	Aminoglycoside 6'-N-acetyltransferase	3045	
1924	278	FIG01181601: hypothetical protein	3046	
1925	181	Deoxycytidine triphosphate deaminase (EC 3.5.4.13)	3047	
1926	200	Peptidyl-prolyl cis-trans isomerase (EC 5.2.1.8)	3048	
1927	360	ATP phosphoribosyltransferase regulatory subunit (EC 2.4.2.17)	3049	17
1928	204	ATP phosphoribosyltransferase (EC 2.4.2.17)	3050	17
1929	195	tRNA (guanosine(18)-2'-O)-methyltransferase (EC 2.1.1.34)	3051	17
1930	80	hypothetical protein	3052	17
1931	58	FIG01181609: hypothetical protein	3053	17
1932	319	Branched-chain amino acid aminotransferase (EC 2.6.1.42)	3054	17
1933	303	Hydrogen peroxide-inducible genes activator	3055	17
1934	303	Manganese catalase (EC 1.11.1.6)	3056	17
1935	334	Competence protein DprA	3057	17
1936	302	GTP-binding protein Era	3058	17
1937	294	Galactoside O-acetyltransferase	3059	17
1938	270	Sulfur carrier protein adenylyltransferase ThiF	3060	17
1939	378	Citrate synthase (si) (EC 2.3.3.1)	3061	17
1940	382	LSU m5C1962 methyltransferase RlmI	3062	17
1941	312	Protease IV (Signal peptide peptidase) (EC 3.4.21.-)	3063	17
1942	135	FIG01181173: hypothetical protein	3064	17
1943	393	IS605 family transposase	3065	17
1944	91	Mobile element protein	3044	18
1945	522	FIG01181127: hypothetical protein	3043	18
1946	114	Alkaline shock protein	3042	18
1947	370	Alanine dehydrogenase (EC 1.4.1.1)	3041	18
1948	313	tRNA pseudouridine synthase B (EC 4.2.1.70)	3040	18
1949	263	Enoyl-CoA hydratase (EC 4.2.1.17)	3039	18
1950	236	Ribosomal large subunit pseudouridine synthase B (EC 4.2.1.70)	3038	18
1951	182	Hypoxanthine-guanine phosphoribosyltransferase (EC 2.4.2.8)	3037	18
1952	223	Putative preQ0 transporter	3036	18
1953	295	L-fuco-beta-pyranose dehydrogenase (EC 1.1.1.122)	3035	18
1954	391	Adenylosuccinate synthetase (EC 6.3.4.4)	3034	18
1955	184	hypothetical protein	3033	18
1956	330	Anthranilate phosphoribosyltransferase (EC 2.4.2.18)	3032	18
1957	194	Anthranilate synthase, amidotransferase component (EC 4.1.3.27)	3031	18
1958	463	Anthranilate synthase, aminase component (EC 4.1.3.27)	3030	18
1959	517	Delta-1-pyrroline-5-carboxylate dehydrogenase (EC 1.5.1.12)	3029	18
1960	308	Proline dehydrogenase (EC 1.5.99.8) (Proline oxidase)	3028	18
1961	220	Predicted regulator PutR for proline utilization, GntR family	3027	18
1962	169	Mobile element protein	3026	18
1963	297	Transcriptional regulator		
1964	394	2-amino-3-ketobutyrate coenzyme A ligase (EC 2.3.1.29)		
1965	422	Modification methylase TaqI (EC 2.1.1.72)		
1966	129	FIG01181172: hypothetical protein	3763	7
1967	642	DNA gyrase subunit B (EC 5.99.1.3)	3762	7
1968	754	ATP-dependent helicase HrpB	3761	7
1969	143	FIG01180852: hypothetical protein	3760	7
1970	131	FIG01181777: hypothetical protein	3759	7
1971	345	Dehydrogenase	3758	7
1972	138	FIG01181515: hypothetical protein	3757	7
1973	337	FIG01181013: hypothetical protein	3756	7

1974	348	FIG01181544: hypothetical protein	3756	7
1975	824	FIG01181618: hypothetical protein	3754	7
1976	325	hypothetical membrane spanning protein	3753	7
1977	228	Short-chain dehydrogenase/reductase SDR	3752	7
1978	119	FIG01180931: hypothetical protein	3751	7
1979	131	Probable methylmalonyl-coA epimerase	3750	7
1980	541	Fumarate/succinate/L-aspartate dehydrogenases	3749	7
1981	314	Ferredoxin	3748	7
1982	360	Chorismate mutase I (EC 5.4.99.5) / 2-keto-3-deoxy-D-arabino-heptulosonate-7-phosphate synthase	3747	7
1983	248	Twin-arginine translocation protein TatC	3746	7
1984	69	Twin-arginine translocation protein TatA	3745	7
1985	454	N-acetylglucosamine-1-phosphate uridyltransferase (EC 2.7.7.23) / Glucosamine-1-phosphate N-acet	3744	7
1986	281	Prolipoprotein diacylglyceryl transferase (EC 2.4.99.-)	3743	7
1987	80	FIG01181592: hypothetical protein	3742	7
1988	160	Probable membrane protein NMA1128	3741	7
1989	354	Alanine dehydrogenase (EC 1.4.1.1)	3740	7
1990	441	4-hydroxybutyrate coenzyme A transferase	3739	7
1991	834	DNA topoisomerase I (EC 5.99.1.2)	3738	7
1992	393	Vancomycin B-type resistance protein VanW	3737	7
1993	237	Ribosomal small subunit pseudouridine synthase A (EC 4.2.1.70)	3736	7
1994	110	hypothetical protein	3735	7
1995	56	hypothetical protein	3734	7
1996	376	Alanyl-tRNA synthetase family protein	3733	7
1997	257	Dehydrogenase	3732	7
1998	178	Thioredoxin reductase (EC 1.8.1.9)	3731	7
1999	439	Serine/threonine protein kinase	3730	7
2000	113	Endonuclease (EC 3.1.-.-)	3729	7
2001	596	UbiD family decarboxylase associated with menaquinone via futalosine	3728	7
2002	316	Two-component response regulator	3726	7
2003	184	FIG01181258: hypothetical protein	3725	7
2004	312	tRNA (guanine46-N7-)-methyltransferase (EC 2.1.1.33)	3724	7
2005	357	Aspartate aminotransferase (EC 2.6.1.1)	3723	7
2006	155	Small heat shock protein	3722	7
2007	231	Thiosulfate sulfurtransferase, rhodanese (EC 2.8.1.1)	3721	7
2008	208	Hydroxyacylglutathione hydrolase (EC 3.1.2.6)	3720	7
2009	244	Flagellar protein FliJ	3719	7
2010	143	Osmotically inducible protein C	3718	7
2011	259	Phosphoglyceromutase	3717	7
2012	332	Ferric iron ABC transporter, iron-binding protein	3716	7
2013	516	Ferric iron ABC transporter, permease protein	3715	7
2014	346	Ferric iron ABC transporter, ATP-binding protein	3714	7
2015	260	tRNA pseudouridine synthase A (EC 4.2.1.70)	3713	7
2016	397	Dipeptide transport system permease protein DppC (TC 3.A.1.5.2)	3712	7
2017	337	Dipeptide transport system permease protein DppB (TC 3.A.1.5.2)	3711	7
2018	616	Dipeptide-binding protein	3710	7
2019	127	Probable iron binding protein from the HesB_IscA_SufA family	3709	7
2020	377	Aminopeptidase YpdF (MP-, MA-, MS-, AP-, NP- specific)	3708	7
2021	311	Ribose-phosphate pyrophosphokinase (EC 2.7.6.1)	3707	7
2022	227	FIG01180848: hypothetical protein	3706	7
2023	196	FIG01181565: hypothetical protein	3705	7
2024	631	Biosynthetic arginine decarboxylase (EC 4.1.1.19)	3704	7
2025	795	hypothetical protein	3703	7
2026	393	S-adenosylmethionine synthetase (EC 2.5.1.6)	3702	7
2027	258	Glutamate racemase (EC 5.1.1.3)	3701	7
2028	98	FIG01181129: hypothetical protein	3700	7
2029	204	Nucleoside 5-triphosphatase RdgB (dHAPTP, dITP, XTP-specific) (EC 3.6.1.15)	3699	7
2030	344	hypothetical protein	3698	7
2031	399	Maltose/maltodextrin ABC transporter, substrate binding periplasmic protein MalE	3696	7
2032	443	Maltose/maltodextrin ABC transporter, permease protein MalF	3695	7
2033	440	Maltose/maltodextrin ABC transporter, permease protein MalG	3694	7
2034	570	Maltodextrin glucosidase (EC 3.2.1.20)	3693	7
2035	98	hypothetical protein	3692	7
2036	753	Recombination inhibitory protein MutS2	3691	7
2037	156	Arginine/ornithine antiporter ArcD	3690	7
2038	140	4-hydroxybenzoyl-CoA thioesterase family active site	3689	7
2039	565	Oligoendopeptidase F (EC 3.4.24.-)	3688	7
2040	151	Arsenate reductase (EC 1.20.4.1)	3687	7
2041	234	2-heptaprenyl-1,4-naphthoquinone methyltransferase MenG (EC 2.1.1.163)	3686	7

2042	610	ABC transporter, permease protein	3685	7
2043	304	Unspecified monosaccharide ABC transport system, permease component 2	3683	7
2044	87	FIG01181137: hypothetical protein	3682	7
2045	179	FIG01181614: hypothetical protein	3681	7
2046	256	Signal peptidase-like protein	3680	7
2047	270	DNA polymerase III delta prime subunit (EC 2.7.7.7)	3679	7
2048	241	Serine/threonine protein phosphatase (EC 3.1.3.16)	3678	7
2049	208	FIG01181442: hypothetical protein	3677	7
2050	67	FIG01181035: hypothetical protein	3676	7
2051	141	FIG01181415: hypothetical protein	3675	7
2052	320	FIG01181334: hypothetical protein	3674	7
2053	408	Permease of the major facilitator superfamily	3673	7
2054	58	hypothetical protein	3672	7
2055	44	hypothetical protein		
2056	88	hypothetical protein	3671	7
2057	53	hypothetical protein		
2058	52	hypothetical protein	3670	7
2059	327	Atypical L-asparaginase (EC 3.5.1.1), Rhizobium type	3669	7
2060	91	hypothetical conserved protein	3668	7
2061	221	Translation initiation factor IF-2	3667	7
2062	186	Molybdenum cofactor biosynthesis protein MoaD	3666	7
2063	608	Tungsten-containing aldehyde:ferredoxin oxidoreductase (EC 1.2.7.5)	3665	7
2064	301	Fructokinase (EC 2.7.1.4)	3664	7
2065	315	Quinone oxidoreductase (EC 1.6.5.5)	3663	7
2066	140	FIG01181767: hypothetical protein	3662	7
2067	184	hypothetical membrane spanning protein	3661	7
2068	97	Transcriptional regulator, marR/emrR family	3660	7
2069	350	NADH-dependent flavin oxidoreductase	3659	7
2070	978	Amylopullulanase	3658	7
2071	222	FIG01181025: hypothetical protein	3657	7
2072	157	Nudix family (d)NDPase DR0975	3656	7
2073	355	Peptide chain release factor 1	3655	7
2074	266	Metal transporter, ZIP family	3654	7
2075	109	hypothetical protein	3653	7
2076	201	Cytochrome oxidase biogenesis protein Sco1/SenC/PrrC, putative copper metallochaperone	3652	7
2077	140	Copper metallochaperone, bacterial analog of Cox17 protein	3651	7
2078	400	Nitrous oxide reductase maturation protein NosD	3650	7
2079	227	Ferric iron ABC transporter, ATP-binding protein	3649	7
2080	235	hypothetical protein	3648	7
2081	113	hypothetical protein	3647	7
2082	169	hypothetical protein	3646	7
2083	250	hypothetical protein	3645	7
2084	149	FIG01181023: hypothetical protein	3644	7
2085	198	Nucleoside triphosphate pyrophosphohydrolase MazG (EC 3.6.1.8)	3643	7
2086	157	Hypothetical nudix hydrolase YeaB	3642	7
2087	56	ThiJ/PfpI family protein	3641	7
2088	250	Acetylglutamate kinase (EC 2.7.2.8)	3640	7
2089	163	FIG01181081: hypothetical protein	3639	7
2090	544	DNA polymerase III subunits gamma and tau (EC 2.7.7.7)	3638	7
2091	94	FIG01181429: hypothetical protein	3637	7
2092	155	FIG01181616: hypothetical protein	3636	7
2093	617	2-oxoglutarate oxidoreductase, alpha subunit (EC 1.2.7.3)	3635	7
2094	305	2-oxoglutarate oxidoreductase, beta subunit (EC 1.2.7.3)	3634	7
2095	158	Molybdenum cofactor biosynthesis protein MoaC	3633	7
2096	113	hypothetical protein	3632	7
2097	318	FIG01181707: hypothetical protein	3631	7
2098	487	Exopolysaccharide biosynthesis protein related to N-acetylglucosamine-1-phosphodiester alpha-N-acetylglucosaminidase		
2099	477	RNA-2',3'-PO4:RNA-5'-OH ligase	4078	5
2100	309	Putative protein phosphatase	4077	5
2101	138	Nucleoside diphosphate kinase (EC 2.7.4.6)	4076	5
2102	182	FIG01181311: hypothetical protein	4075	5
2103	45	hypothetical protein		
2104	119	Dihydroneopterin aldolase (EC 4.1.2.25)	4074	5
2105	273	Dihydropteroate synthase (EC 2.5.1.15)	4073	5
2106	86	Universal stress protein family	4072	5
2107	403	Permease	4071	5
2108	180	TRAP dicarboxylate transporter, DctQ subunit, unknown substrate 6	4070	5
2109	498	TRAP dicarboxylate transporter, DctM subunit, unknown substrate 6	4069	5

2110	360	TRAP transporter solute receptor, unknown substrate 6	4068	5
2111	158	FIG01180918: hypothetical protein	4067	5
2112	201	Ribonuclease HII (EC 3.1.26.4)	4066	5
2113	496	Type B carboxylesterase	4065	5
2114	668	Molybdopterin oxidoreductase	4064	5
2115	137	FIG01180951: hypothetical protein	4063	5
2116	407	tRNA S(4)U 4-thiouridine synthase (former ThiI)	4062	5
2117	435	Asp-tRNAAsn/Glu-tRNAGln amidotransferase A subunit and related amidases	4061	5
2118	397	Arsenic efflux pump protein	4060	5
2119	203	Ribosomal-protein-alanine acetyltransferase (EC 2.3.1.128)	4059	5
2120	348	Putative aminopeptidase	4058	5
2121	165	Molybdenum cofactor biosynthesis protein MoaB	4057	5
2122	378	Aspartate aminotransferase (EC 2.6.1.1)	4056	5
2123	70	Adenine deaminase	4055	5
2124	282	hypothetical protein	4054	5
2125	543	Mobile element protein	4053	5
2126	435	Subtilisin precursor	4052	5
2127	560	TPR repeat	4051	5
2128	415	Dihydrofolate synthase (EC 6.3.2.12) @ Folylpolyglutamate synthase (EC 6.3.2.17)	4050	5
2129	119	Peroxide stress regulator PerR, FUR family	4049	5
2130	304	Porphobilinogen deaminase (EC 2.5.1.61)	4048	5
2131	444	Dihydrolipoamide acetyltransferase component of pyruvate dehydrogenase complex (EC 2.3.1.12)	4047	5
2132	906	Pyruvate dehydrogenase E1 component (EC 1.2.4.1)	4046	5
2133	286	Transcriptional regulatory protein, lysR family	4045	5
2134	86	FIG01181667: hypothetical protein	4044	5
2135	88	FIG01181049: hypothetical protein	4043	5
2136	148	Ferric uptake regulation protein FUR	4042	5
2137	452	Cytosol aminopeptidase PepA (EC 3.4.11.1)	4041	5
2138	273	FIG069887: hypothetical protein	4040	5
2139	372	tRNA-specific 2-thiouridylase MnmA	4039	5
2140	324	Phosphate ABC transporter, periplasmic phosphate-binding protein PstS (TC 3.A.1.7.1)	4038	5
2141	318	Phosphate transport system permease protein PstC (TC 3.A.1.7.1)	4037	5
2142	402	Phosphate transport system permease protein PstA (TC 3.A.1.7.1)	4036	5
2143	271	Phosphate transport ATP-binding protein PstB (TC 3.A.1.7.1)	4035	5
2144	391	Multidrug-efflux transporter	4034	5
2145	344	DNA recombination and repair protein RecF	4033	5
2146	253	Zn-ribbon-containing, possibly RNA-binding protein and truncated derivatives	4032	5
2147	65	YcfA protein	4031	5
2148	74	hypothetical protein	4030	5
2149	70	hypothetical protein		
2150	255	hypothetical protein	4029	5
2151	72	hypothetical protein	4028	5
2152	299	hypothetical conserved protein	4027	5
2153	574	DNA primase (EC 2.7.7.-)	4026	5
2154	204	hypothetical protein	4025	5
2155	86	hypothetical protein	4024	5
2156	148	hypothetical protein	4023	5
2157	100	hypothetical protein	4022	5
2158	230	ParB like nuclease domain containing protein	4021	5
2159	78	hypothetical protein	4020	5
2160	44	hypothetical protein		
2161	392	Transposase	4019	5
2162	230	FIG01181326: hypothetical protein	4018	5
2163	792	Cytochrome c oxidase polypeptide I (EC 1.9.3.1) / Cytochrome c oxidase polypeptide III (EC 1.9.3.1)	4017	5
2164	338	Cytochrome c oxidase polypeptide II (EC 1.9.3.1)	4016	5
2165	636	Heme A synthase, cytochrome oxidase biogenesis protein Cox15-CtaA	4015	5
2166	530	Phosphoenolpyruvate carboxykinase [ATP] (EC 4.1.1.49)	4014	5
2167	418	Glucose-6-phosphate isomerase (EC 5.3.1.9)	4013	5
2168	134	Putative uncharacterized protein TTHB069	4012	5
2169	396	hypothetical protein	4011	5
2170	291	Zinc ABC transporter, periplasmic-binding protein ZnuA	4010	5
2171	144	hypothetical protein	4009	5
2172	394	hypothetical protein	4008	5
2173	316	FIG01181701: hypothetical protein	4007	5
2174	135	hypothetical conserved protein	4005	5
2175	57	FIG01181111: hypothetical protein	4004	5
2176	661	Threonyl-tRNA synthetase (EC 6.1.1.3)	4003	5
2177	260	Phenylacetic acid degradation operon negative regulatory protein PaaX	4002	5

2178	396	Glycerol-3-phosphate ABC transporter, periplasmic glycerol-3-phosphate-binding protein (TC 3.A.1.1.	4001	5
2179	399	Glycerol-3-phosphate ABC transporter, periplasmic glycerol-3-phosphate-binding protein (TC 3.A.1.1.	3999	5
2180	254	Sugar phosphate isomerases/epimerases	3998	5
2181	241	Ubiquinol cytochrome C oxidoreductase, cytochrome C1 subunit	3997	5
2182	161	Ubiquinol cytochrome C oxidoreductase, cytochrome C1 subunit	3996	5
2183	210	Ubiquinol-cytochrome C reductase iron-sulfur subunit (EC 1.10.2.2)	3995	5
2184	421	Ubiquinol-cytochrome C reductase, cytochrome B subunit (EC 1.10.2.2)	3994	5
2185	531	SSU ribosomal protein S1p	3993	5
2186	372	Carboxypeptidase G2 (EC 3.4.17.11)	3992	5
2187	106	Uncharacterized protein SCO3165	3991	5
2188	92	FIG01180982: hypothetical protein	3990	5
2189	57	hypothetical protein	3989	5
2190	133	FIG01181273: hypothetical protein	3988	5
2191	452	Sodium/glycine symporter GlyP	3987	5
2192	267	FIG01181404: hypothetical protein	3986	5
2193	231	Carboxylesterase	3985	5
2194	249	Putative divalent heavy-metal cations transporter	3984	5
2195	221	Sugar/maltose fermentation stimulation protein homolog	3983	5
2196	297	hypothetical protein	3982	5
2197	339	Integral membrane protein	3981	5
2198	470	FIG01181014: hypothetical protein	3980	5
2199	351	COG1180: Radical SAM, Pyruvate-formate lyase-activating enzyme like	3979	5
2200	265	COG1355, Predicted dioxygenase	3978	5
2201	185	COG1355, Predicted dioxygenase	3977	5
2202	130	FIG01180884: hypothetical protein	3976	5
2203	250	Putative FMN hydrolase (EC 3.1.3.-); 5-Amino-6-(5'-phosphoribitylamino)uracil phosphatase	3975	5
2204	123	FIG01180908: hypothetical protein	3974	5
2205	172	FIG01181162: hypothetical protein	3973	5
2206	145	Uncharacterized protein MJ0531	3972	5
2207	239	23S rRNA (guanosine-2'-O-) -methyltransferase rlmB (EC 2.1.1.-)	3971	5
2208	357	Acetylornithine deacetylase (EC 3.5.1.16)	3970	5
2209	79	FIG01181024: hypothetical protein	3969	5
2210	303	Glucokinase (EC 2.7.1.2)	3968	5
2211	71	FIG01181320: hypothetical protein	3967	5
2212	205	GTP cyclohydrolase I (EC 3.5.4.16) type 1	3966	5
2213	230	Ferripyochelin binding protein	3965	5
2214	127	Bis(5'-nucleosyl)-tetraphosphatase (Asymmetrical) (EC 3.6.1.17)	3964	5
2215	288	hypothetical protein	3963	5
2216	588	GTP-binding protein TypA/BipA	3962	5
2217	228	FIG01180859: hypothetical protein	3961	5
2218	420	ATPase component BioM of energizing module of biotin ECF transporter	3960	5
2219	262	Maltose/maltodextrin ABC transporter, permease protein MalG	3959	5
2220	122	UPF0331 protein MJ0127	3958	5
2221	107	nucleotidyltransferase	3957	5
2222	403	L-carnitine dehydratase/bile acid-inducible protein F	3956	5
2223	293	ABC transporter, ATP-binding protein	3954	5
2224	257	FIG01181230: hypothetical protein	3953	5
2225	355	Aminopeptidase	3952	5
2226	334	Dihydrolipoamide acetyltransferase component of pyruvate dehydrogenase complex (EC 2.3.1.12)	3950	5
2227	666	Excinuclease ABC subunit B	3949	5
2228	911	S-layer protein	3948	5
2229	605	Glucosamine--fructose-6-phosphate aminotransferase [isomerizing] (EC 2.6.1.16)	3947	5
2230	523	HD-hydrolase domain	3946	5
2231	405	hypothetical membrane associated protein	3945	5
2232	326	Thioredoxin reductase (EC 1.8.1.9)	3944	5
2233	199	hypothetical protein	3943	5
2234	564	FIG01181065: hypothetical protein	3942	5
2235	305	Methionyl-tRNA formyltransferase (EC 2.1.2.9)	3941	5
2236	191	Peptide deformylase (EC 3.5.1.88)	3940	5
2237	271	Uncharacterized secreted protein associated with spyDAC	3939	5
2238	238	Diadenylate cyclase spyDAC; Bacterial checkpoint controller DisA with nucleotide-binding domain	3938	5
2239	382	Phosphopentomutase (EC 5.4.2.7)	3937	5
2240	86	FIG01181645: hypothetical protein	3936	5
2241	324	ATPase, AFG1 family	3935	5
2242	90	Glutaredoxin	3934	5
2243	162	FIG01181674: hypothetical protein	3933	5
2244	89	hypothetical protein	3932	5
2245	146	hypothetical protein	3931	5

2246	268	Enoyl-[acyl-carrier-protein] reductase [NADH] (EC 1.3.1.9)	3930	5
2247	138	Conserved protein	3929	5
2248	130	hypothetical conserved protein	3928	5
2249	306	Glyoxalase family protein	3927	5
2250	408	1-hydroxy-2-methyl-2-(E)-butenyl 4-diphosphate synthase (EC 1.17.7.1)	3926	5
2251	386	tRNA-guanine transglycosylase (EC 2.4.2.29)	3925	5
2252	412	Uracil permease	3924	5
2253	366	FIG01181160: hypothetical protein	3923	5
2254	232	Arginine/ornithine antiporter ArcD	3922	5
2255	99	LSU ribosomal protein L28p @ LSU ribosomal protein L28p, zinc-independent	3921	5
2256	147	Lipoprotein signal peptidase (EC 3.4.23.36)	3920	5
2257	374	Biosynthetic Aromatic amino acid aminotransferase alpha (EC 2.6.1.57) @ Aspartate aminotransferas	3919	5
2258	306	Diacylglycerol kinase-related protein	3918	5
2259	217	Phosphoglycerate mutase/fructose-2,6-bisphosphatase	3917	5
2260	285	Acetyltransferase	3916	5
2261	362	Deacetylase	3915	5
2262	159	tRNA (cytidine(34)-2'-O)-methyltransferase (EC 2.1.1.207)	3914	5
2263	513	ATP-dependent DNA helicase RecG-related protein	3913	5
2264	431	Ammonium transporter	3912	5
2265	117	Nitrogen regulatory protein P-II	3911	5
2266	301	Heat-inducible transcription repressor HrcA	3910	5
2267	224	Molybdenum cofactor biosynthesis protein MoaD / Molybdenum cofactor biosynthesis protein MoaE	3909	5
2268	75	hypothetical protein	3908	5
2269	180	hypothetical protein	3907	5
2270	350	Ribosomal RNA large subunit methyltransferase N (EC 2.1.1.-)	3906	5
2271	388	Butyryl-CoA dehydrogenase (EC 1.3.99.2)	3905	5
2272	233	FIG01181641: hypothetical protein	3904	5
2273	879	Leucyl-tRNA synthetase (EC 6.1.1.4)	3903	5
2274	261	FIG01181167: hypothetical protein	3902	5
2275	187	FIG01181093: hypothetical protein	3901	5
2276	414	Gamma-glutamyl phosphate reductase (EC 1.2.1.41)	3900	5
2277	370	Glutamate 5-kinase (EC 2.7.2.11) / RNA-binding C-terminal domain PUA	3899	5
2278	71	FIG01181328: hypothetical protein	3898	5
2279	188	ATP:Cob(I)alamin adenosyltransferase (EC 2.5.1.17)	3897	5
2280	92	FIG01181373: hypothetical protein	3896	5
2281	240	ABC transporter ATP-binding protein	3895	5
2282	393	Sodium ABC transporter, permease protein NatB	3894	5
2283	286	Membrane protease family protein BA0301	3893	5
2284	67	Conserved domain protein	3892	5
2285	161	hypothetical protein	3891	5
2286	259	CAAX amino terminal protease family protein	3890	5
2287	273	Hypothetical ATP-binding protein UPF0042, contains P-loop	3889	5
2288	406	Transporter	3888	5
2289	233	FIG01181316: hypothetical protein	3887	5
2290	107	FIG01181351: hypothetical protein	3886	5
2291	362	Alanine racemase (EC 5.1.1.1)	3885	5
2292	707	Helicase PriA essential for oriC/DnaA-independent DNA replication	3884	5
2293	350	Multidrug resistance protein, putative	3883	5
2294	220	Putative glycolate oxidase subunit E	3882	5
2295	353	Phenylalanyl-tRNA synthetase alpha chain (EC 6.1.1.20)	4646	14
2296	784	Phenylalanyl-tRNA synthetase beta chain (EC 6.1.1.20)	4645	14
2297	154	FIG01181148: hypothetical protein	4644	14
2298	323	6-phosphofructokinase (EC 2.7.1.11)	4643	14
2299	263	rRNA small subunit methyltransferase I	4642	14
2300	133	FIG01181036: hypothetical protein	4641	14
2301	176	Inorganic pyrophosphatase (EC 3.6.1.1)	4640	14
2302	210	Glycerophosphoryl diester phosphodiesterase (EC 3.1.4.46)	4639	14
2303	205	FIG01181553: hypothetical protein	4638	14
2304	103	Competence protein/ComEA-related protein	4637	14
2305	677	Late competence protein ComEC, DNA transport	4636	14
2306	269	Chromosome (plasmid) partitioning protein ParB	4635	14
2307	250	Chromosome (plasmid) partitioning protein ParA	4634	14
2308	243	rRNA small subunit 7-methylguanosine (m7G) methyltransferase GidB	4633	14
2309	597	tRNA uridine 5-carboxymethylaminomethyl modification enzyme GidA	4632	14

Plasmid Gene	Length	function	JGI ORF#	JGI Contig
pTA16-1	77	hypothetical protein	3124	19
pTA16-2	78	hypothetical protein	3125	19

pTA16-3	120	hypothetical protein - Mut7-C RNAse domain	3126	19
pTA16-4	241	hypothetical protein	3127	19
pTA16-5	202	peptidase C39 bacteriocin processing	3128	19
pTA16-6	374	mobile element protein - Transposase DDE domain	3129	19
pTA16-7	86	hypothetical protein - Transposase	3130	19
pTA16-8	82	hypothetical protein - Transposase	3131	19
pTA16-9	87	hypothetical protein - Transposase	3132	19
pTA16-10	354	mobile element protein - Integrase core domain	3133	19
pTA16-11	281	hypothetical protein - Transposase DDE domain	3134	19
pTA16-12	244	hypothetical protein - membrane protein	3135	19
pTA16-13	235	hypothetical protein - Plasmid replication region DNA-binding N-term	3136	19
			3137	19
pTA16-14	116	hypothetical protein		
pTA16-15	250	hypothetical protein	3138	19
pTA16-16	116	Transposase	3139	19
pTA16-17	233	hypothetical protein		
			3140	19
pTA16-18	410	hypothetical protein - replication protein	3141	19
pTA16-19	83	hypothetical protein	3142	19
pTA16-20	357	hypothetical protein	3143	19
pTA14-1	438	hypothetical protein - membrane protein	3145	20
pTA14-2	95	hypothetical protein	3146	20
pTA14-3	61	hypothetical protein		
pTA14-4	179	hypothetical protein - transcriptional regulator, LuxR family	3147	20
pTA14-5	301	hypothetical protein	3148	20
pTA14-6	280	peptide modification radical SAM enzyme, YydG family	3149	20
pTA14-7	116	hypothetical protein - SdpI/YhfL protein family	3150	20
pTA14-8	381	putative methyltransferase	3151	20
pTA14-9	388	putative beta-lactamase	3152	20
pTA14-10	77	hypothetical protein		
pTA14-11	144	hypothetical protein		
pTA14-12	66	hypothetical protein	3153	20
pTA14-13	108	hypothetical protein	3154	20
pTA14-14	90	hypothetical protein	3155	20
pTA14-15	91	hypothetical protein		20
pTA14-16	90	hypothetical protein	3156	20
pTA14-17	397	primase 1 - Replicase family	3157	20
pTA14-18	117	hypothetical protein	3158	20
pTA14-19	255	hypothetical protein	3159	20
pTA14-20	76	hypothetical protein		
pTA14-21	105	hypothetical protein		
pTA69-1	66	hypothetical protein		
pTA69-2	101	hypothetical protein	3329	15
pTA69-3	199	peptidase, membrane zinc metallopeptidase	3330	15
pTA69-4	185	hypothetical protein - membrane protein	3331	15
pTA69-5	232	hypothetical protein - helix_turn_helix, Arsenical Resistance Operon Repressor	3332	15
pTA69-6	88	hypothetical protein	3332	15
pTA69-7	353	Chromosome partition protein smc - Prefoldin, chaperonin cofactor	3334	15
pTA69-8	120	hypothetical protein - membrane protein	3335	15
pTA69-9	369	hypothetical protein - membrane protein	3336	15
			3337	15
pTA69-10	471	S-layer protein	3338	15
pTA69-11	319	Putative regulatory protein, contains AAA+ - stage III sporulation protein AA	3339	15
pTA69-12	103	hypothetical protein - membrane protein	3340	15
pTA69-13	152	Lytic transglycosylase, catalytic	3341	15
pTA69-14	78	hypothetical protein	3342	15
pTA69-15	96	hypothetical protein	3343	15
pTA69-16	814	Type IV secretory pathway putative VirD4 protein - membrane protein	3344	15
pTA69-17	99	hypothetical protein	3345	15
pTA69-18	633	hypothetical protein - membrane protein	3346	15
pTA69-19	519	hypothetical protein - conjugal transfer protein TraD	3347	15
pTA69-20	826	Conjugative transfer gene TrsE homolog, ATPase - Type IV secretory pathway, VirB4 component - Tra	3348	15
pTA69-21	105	hypothetical protein - membrane protein	3349	15
pTA69-22	128	hypothetical protein - membrane protein - TrbC/VIRB2 family; Conjugal transfer protein	3350	15
pTA69-23	554	hypothetical protein - membrane protein	3351	15
pTA69-24	166	hypothetical protein	3352	15

pTA69-25	419	mobile element protein -transposase	3353	15
pTA69-26	58	hypothetical protein	3354	15
pTA69-27	112	hypothetical protein - membrane protein	3355	15
pTA69-28	74	hypothetical protein - membrane protein	3356	15
pTA69-29	161	hypothetical protein	3357	15
pTA69-30	288	hypothetical protein - secreted	3358	15
pTA69-31	304	hypothetical protein - membrane protein	3359	15
pTA69-32	308	hypothetical protein	3360	15
pTA69-33	131	hypothetical protein	3361	15
pTA69-34	770	hypothetical protein ABC transporter ATPase	3362	15
pTA69-35	127	hypothetical protein - membrane protein	3363	15
pTA69-36	202	hypothetical protein - Ribonuclease H-like superfamily	3364	15
pTA69-37	366	hypothetical protein - Winged helix-turn-helix transcription repressor, HrcA DNA-binding	3365	15
pTA69-38	197	hypothetical protein	3366	15
pTA69-39	154	hypothetical protein	3367	15
pTA69-40	96	hypothetical protein	3368	15
pTA69-41	220	Micrococcal nuclease	3369	15
pTA69-42	1214	Modification methylase BstVI - Type I restriction-modification system methyltransferase subunit	3370	15
pTA69-43	292	Chromosome (plasmid) partitioning protein ParB	3371	15
pTA69-44	321	Chromosome (plasmid) partitioning protein ParA	3372	15
pTA69-45	318	hypothetical protein tyrosine recombinase XerD	3373	15
pTA69-46	624	subtilisin-like serine protease	3374	15
pTA69-47	124	hypothetical protein	3375	15
pTA69-48	130	hypothetical protein	3376	15
pTA69-49	65	hypothetical protein	3377	15
pTA69-50	253	hypothetical protein	3378	15
pTA69-51	619	hypothetical protein	3379	15
pTA69-52	618	hypothetical protein - membrane protein	3380	15
pTA69-53	396	hypothetical protein - secreted	3281	15
			3282	15
pTA69-54	98	hypothetical protein	3383	15
pTA69-55	125	hypothetical protein	3384	15
pTA69-56	205	hypothetical protein - membrane protein	3385	15
pTA69-57	81	hypothetical protein	3386	15
pTA69-58	79	hypothetical protein	3387	15
pTA69-59	318	hypothetical protein - homolog of pTA78-56	3388	15
pTA69-60	66	hypothetical protein	3389	15
pTA69-61	84	hypothetical protein	3390	15
pTA69-62	174	Rad52/22 family double-strand break repair protein	3391	15
pTA69-63	78	hypothetical protein	3392	15
pTA69-64	57	hypothetical protein	3393	15
pTA69-65	57	hypothetical protein	3394	15
pTA69-66	100	hypothetical protein	3395	15
pTA69-67	125	hypothetical protein	3396	15
pTA69-68	92	hypothetical protein	3397	15
pTA69-69	102	hypothetical protein	3398	15
pTA69-70	125	Single-stranded DNA-binding protein	3399	15
pTA69-71	150	hypothetical protein	3005	21
pTA69-72	83	hypothetical protein	3006	21
pTA69-73	106	hypothetical protein	3007	21
pTA69-74	60	hypothetical protein	3008	21
pTA69-75	680	Superfamily II DNA/RNA helicases, SNF2 family	3009	21
pTA69-76	157	hypothetical protein	3010	21
pTA69-77	245	hypothetical protein - Ribonuclease H-like superfamily	3011	21
pTA69-78	109	hypothetical protein	3012	21
pTA69-79	361	hypothetical protein - membrane protein	3013	21
pTA69-80	67	hypothetical protein	3014	21
pTA69-81	137	hypothetical protein	3015	21
pTA69-82	110	hypothetical protein	3016	21
pTA69-83	114	hypothetical protein	3017	21
pTA69-84	66	hypothetical protein	3018	21
pTA69-85	123	hypothetical protein	3019	21
pTA69-86	108	hypothetical protein	3020	21
pTA69-87	53	hypothetical protein	3021	21
pTA69-88	80	hypothetical protein	3022	21
pTA69-89	76	hypothetical protein		
pTA69-90	327	hypothetical protein	3023	21
pTA69-91	251	RNA polymerase sigma-70 factor	3024	21

pTA69-92	107	hypothetical protein		
pTA78-1	350	Helicase, C-terminal:Type III restriction enzyme DEAD	3163	13
pTA78-2	1024	Helicase, C-terminal:Type III restriction enzyme	3164	13
pTA78-3	112	hypothetical protein	3165	13
pTA78-4	154	hypothetical protein	3166	13
pTA78-5	194	hypothetical protein	3167	13
pTA78-6	361	hypothetical protein - Winged helix-turn-helix transcription repressor	3168	13
pTA78-7	73	hypothetical protein		
pTA78-8	213	hypothetical protein - Transposase IS4	3169	13
pTA78-9	416	Mobile element protein	3170	13
pTA78-10	86	hypothetical protein	3171	13
pTA78-11	89	hypothetical protein	3172	13
pTA78-12	82	hypothetical protein	3173	13
pTA78-13	219	Subtilisin-like serine proteases	3174	13
pTA78-14	87	hypothetical protein	3175	13
pTA78-15	138	plasmid stability protein stbB - PIN domain of ribonucleases (toxins), VapC and FitB, of prokaryotic to	3176	13
pTA78-16	49	mobile element protein		
pTA78-17	1684	ATP-dependent DNA helicase RecQ	3177	13
pTA78-18	323	hypothetical protein Chromosome partition protein _SMC	3178	13
pTA78-19	157	hypothetical protein Chromosome partition protein _SMC	3179	13
pTA78-20	502	hypothetical protein Transcription antiterminator BglG	3180	13
pTA78-21	231	hypothetical protein Transposase IS4 family protein	3181	13
pTA78-22	164	hypothetical protein CHRD domain containing protein	3182	13
pTA78-23	210	hypothetical protein	3183	13
pTA78-24	402	hypothetical protein	3184	13
pTA78-25	205	hypothetical protein	3185	13
pTA78-26	188	RNA polymerase sigma-70 factor	3186	13
pTA78-27	261	Heme ABC transporter, ATPase component HmuV	3187	13
pTA78-28	343	Heme ABC transporter, ATPase component HmuU	3188	13
			3189	13
pTA78-29	303	Heme ABC transporter, ATPase component HmuT	3190	13
pTA78-30	210	FIG078613: hypothetical protein in iron scavenging cluster	3191	13
pTA78-31	105	Heme-degrading oxygenase, IsdG-like	3192	13
pTA78-32	287	putative ABC transporter (substrate-binding protein)	3193	13
pTA78-33	135	Acyl-CoA hydrolase (EC 3.1.2.20	3194	13
pTA78-34	323	Ribonucleotide reductase of class Ia (aerobic) beta subunit	3195	13
pTA78-35	557	Ribonucleotide reductase of class Ia (aerobic) alpha subunit	3196	13
			3197	13
pTA78-36	955	ORF182 Superfamily I DNA and RNA helicases - ankyrin repeats	3198	13
pTA78-37	542	Mobile element protein - Transposase	3199	13
pTA78-38	459	hypothetical protein -Plasmid replication initiator protein	3200	13
pTA78-39	50	hypothetical protein		
pTA78-40	74	hypothetical protein - CopG family transcriptional regulator	3201	13
pTA78-41	137	hypothetical protein - twitching motility protein PilT	3202	13
pTA78-42	63	hypothetical protein	3203	13
pTA78-43	80	hypothetical protein	3204	13
pTA78-44	495	hypothetical protein - S-adenosylmethionine-dependent methyltransferase	3205	13
pTA78-45	99	Death on curing protein, Doc toxin	3206	13
pTA78-46	77	Prevent host death protein, Phd antitoxin	3207	13
pTA78-47	111	hypothetical protein	3208	13
pTA78-48	116	hypothetical protein	3209	13
pTA78-49	200	hypothetical protein		13
pTA78-50	351	hypothetical protein - Sel1 domain protein repeat-containing protein	3210	13
			3211	13
pTA78-51	600	hypothetical protein - HTTM domain protein	3212	13
pTA78-52	238	hypothetical protein	3213	13
pTA78-53	138	hypothetical protein - twitching motility protein PilT	3214	13
pTA78-54	83	hypothetical protein - SpoVT / AbrB like domain; This domain is found in AbrB from Bacillus subtilis.	3215	13
pTA78-55	106	hypothetical protein - Piwi_piwi-like_ProArk: PIWI domain, Piwi-like subfamily found in Archaea and	3216	13
pTA78-56	338	hypothetical protein	3217	13
pTA78-57	53	hypothetical protein	3218	13
pTA78-58	180	hypothetical protein - Predicted transcriptional regulators	3219	13
pTA78-59	84	hypothetical protein		
pTA78-60	44	hypothetical protein		
pTA78-61	171	hypothetical protein	3220	13
pTA78-62	176	hypothetical protein - Ribonuclease HI	3221	13
pTA78-63	271	hypothetical protein	3222	13

pTA78-64	366	hypothetical protein - AAA domain; This AAA domain is found in a wide variety of presumed DNA rep	3223	13
pTA78-65	286	hypothetical protein - Transposase IS4	3224	13
pTA78-66	542	Mobile element protein -Transposase	3225	13
pTA78-67	107	AbiD - Abortive infection bacteriophage resistance protein	3226	13
pTA78-68	37	hypothetical protein		
pTA78-69	103	hypothetical protein		
pTA78-70	61	hypothetical protein		
pTA78-71	69	hypothetical protein	3227	13
pTA78-72	318	hypothetical protein - site-specific recombinase XerD	3228	13
pTA78-73	303	hypothetical protein	3229	13
			3330	13
pTA78-74	323	soi protein - ATPases involved in chromosome partitioning, ParA	3231	13
pTA78-75	294	Chromosome (plasmid) partitioning protein ParB	3232	13
pTA78-76	918	Superfamily II DNA/RNA helicases, SNF2 family - DEAD-like helicases superfamily	3233	13
pTA78-77	1309	Possible restriction /modification enzyme -N-6 DNA Methylase	3234	13
pTA78-78	231	hypothetical protein	3235	13
